# The effectiveness and decay of public health policy actions on infection-control behaviour in the general public: Evidence from a low-COVID prevalence jurisdiction

**DOI:** 10.1371/journal.pone.0283711

**Published:** 2023-08-30

**Authors:** Kent Ross, Daniel J. Dutton

**Affiliations:** Community Health and Epidemiology, Dalhousie University, Saint John, New Brunswick, Canada; Queen Mary University of London, UNITED KINGDOM

## Abstract

**Background:**

Public health policies designed to influence individuals’ infection-control behaviour are a tool for governments to help prevent the spread of disease. Findings on the impacts of policies are mixed and there is limited information on the effects of removing restrictions and how policies impact behavioural trends.

**Methods:**

We use low-acuity emergency department visits from 12 hospitals in New Brunswick, Canada, (January 2017 –October 2021) as a proxy for infection-control behaviour and provide insight into the effects of the COVID-19 virus on a population with a low prevalence of cases. Quasi-experimental techniques (event studies) are applied to estimate the magnitude and persistence of effects of specific events (e.g., policy changes), to control for COVID-19 cases and vaccines, and to explore how the effectiveness of policy changes during the pandemic as more policies are introduced.

**Results:**

Initial tightening of restrictions on March 11, 2020 reduced low-acuity emergency department visits by around 60% and reached a minimum after 30 days. Relaxing policies on social gatherings and personal services gradually increased low-acuity emergency department visits by approximately 50% after 44 days. No effects were found from policies lifting all restrictions, and reinstating a state of emergency on July 31, 2021, and September 24, 2021.

**Conclusion:**

These results suggest that policy interventions are less likely to be effective at influencing infection control behaviour with time and more policies introduced, and that tracking and publicly reporting case numbers can influence infection control behaviour.

## 1. Background

Governments in the Western world have opted to balance incidence rates of COVID-19 with some semblance of normal societal function, achieved by alternating between tightening restrictions in response to high incidence and relaxing restrictions in response to low incidence. Actions designed to reduce transmission of the virus (e.g., policies and recommendations on mask wearing, hand washing, physical distancing, etc.) are associated with lower COVID-19 infection rates [1, 2].

Low uptake of government recommendations by the general population counteracts the effectiveness of those actions. The ability to measure changes in risk or behaviour (i.e., effectiveness) after the policy was enacted is useful since the incidence of COVID-19 can increase rapidly, causing strain on already overwhelmed the health care systems [3]. In the absence of one well-defined marker of successful infection-control behaviour change, studies have proposed multiple ways to measure this issue, with mixed results.

Internationally, several studies have identified factors associated with infection control behaviour during the COVID-19 pandemic. They show that responses vary depending on factors like socioeconomic status and prevailing culture. Individuals with higher socioeconomic status, and those from cultures that value avoiding uncertainty, reported a higher perceived risk of COVID-19 [4, 5]. Some evidence points to more democratic countries having fewer cases of COVID-19 [6]. Other population level studies find high vaccine rates to be associated with low death rates, more democratic countries, and high levels of education [7, 8]. From a policy perspective, the introduction of policies like masking, social distancing, or closing schools were found to be associated with lower infection rates among low-unemployment countries [9].

Using cellphone mobility data from the United States, no noteworthy effect on physical mobility from nonessential business closures, bans on large gatherings, school closure mandates, or limits on restaurants and bars was found [1]. Emergency declarations and the first death from COVID-19 increased time spent at home by 22% and 9% but no evidence was found for the association between increased time at home associated and the first confirmed case in the state, school closures, or stay-at-home orders [10].

The health care system is a potentially high-risk location for individuals to be exposed to COVID-19. Some studies show variation in use of health care in response to changes in policy. For instance, outpatient visits to general practitioners (i.e., “health check-ups”) decreased by around 15% during the first two weeks of state closure in the United States, followed by a slight rebound after five weeks [11]. Similarly, using Canadian emergency department (ED) utilization data, ED usage decreased following a state of emergency declaration by 24.3% for patients with high-acuity issues, 40.9% for standard issues, and 79.3% for low acuity issues [12], with the highest proportion of low-acuity ED visits coming disproportionately from neighbourhoods with the highest number of immigrants or visible minority residents [13].

The changes observed in low-acuity ED visits is a promising avenue for studying the effectiveness of COVID-19 related policy changes; low-acuity ED visits are likely at the discretion of the patient, do not constitute emergencies most of the time, and are a convenient substitute for visiting a general practitioner. In Canada, ED visits occur when a patient goes to an emergency facility and is attended to by a physician, there are no referrals to the ED and everyone with public health care coverage can access this service. EDs are stable and usually predictably open, meaning visits typically do not change based on the operation of the ED itself. We propose the adoption of low-acuity ED visits as a proxy for the behavioural change that policies try to inspire. Acuity levels are determined by Canadian Triage and Acuity Scale levels, which range from 1 to 5, and indicate the patient’s need: resuscitation (1), emergent, urgent, less-urgent, and non-urgent (5) [14]. In this study we call less-urgent and non-urgent triage levels “low-acuity ED visits”, which is in line with how those patients expect to wait longer for care. While some Canadians use the ED to access health care for non-emergency issues, during the COVID-19 pandemic visiting EDs increased a patient’s perceived risk of becoming infected with COVID-19. We propose using low-acuity ED visits as a proxy for infection behaviour because it represents a tradeoff between seeking health and avoiding infection, patients weigh the new risk of COVID-19 against the urgency of receiving care. In other words, changes to ED visiting behaviour should only be driven by changes to policy during the COVID-19 pandemic in a low-incidence jurisdiction, not driven by changes in emergency health needs. Consequently, low acuity visits are a potential indicator of how people view the risk of COVID-19 and the consequences of their adherence to government policy; if people change ED visit behaviour, and that correlates with changes in infection control policy, then we suggest those are connected through the resonance of the policy change and salience of COVID-19 risk.

Worldwide, ED visits have been shown to decrease in response to COVID-19’s spread [11, 15, 16]. Reductions in ED visits have been attributed to fear of infection, which in turn has raised concerns about inappropriate avoidance of EDs. However, some studies show the decrease in ED use coincides with relevant policy changes as opposed to the presence of COVID-19 in a region [11, 12]. Thus, ED visits for low-acuity problems could indicate whether individuals are taking other, less obvious, actions to change their risk of COVID-19 infection, and can be used to determine how long that behaviour typically lasts.

Using this reasoning, we expect to observe low-acuity ED visits change when COVID-19 policies are introduced. For example, if restrictions are introduced limiting the size of social gatherings, people may also be more wary of participating in other activities they associate with the transmission of COVID-19 including grocery shopping, going into the workplace/school, or visiting EDs. People are likely to be most effective at changing behaviour when people are most aware of them, therefore we expect the influence of policies on behaviour to decay with the awareness of the policies or decreasing tolerance of those policies (sometimes called “pandemic fatigue” [17].

The length of time a policy remains effective at changing behaviour through measuring changes in health care utilization has been largely ignored by the literature. In general, the size of a policy’s impact on the behaviours it targets (i.e., its effectiveness) is contingent on preceding policies and their effectiveness [18]. This could impact the public’s tolerance for future restrictions, and the consequent effectiveness of those future policies.

We aim to estimate how long it takes for a COVID-19 policy message’s effectiveness to decay. We use data from the Canadian province of New Brunswick (NB), which had uniquely low and consistent incidence of COVID-19 throughout much of 2020 and 2021 compared to the rest of the country [19], meaning behavioural changes observed are likely in reaction to policy changes rather than COVID-19 prevalence or health care system disruption.

The objectives of our study are: (1) To estimate the impact of different COVID-19 related policies implemented at different times on trends in health care consumption through low-acuity ED visits; and (2) measure effects of different types of policies, including when restrictions are tightened or loosened.

## 2. Methods

### 2.1 Policy changes, data, and data sources

The evolution of COVID-19 policies in NB is similar to the rest of Canada: an initial shutdown followed by increasing relaxation of business and social restrictions. A summary of these policy changes is presented in Table 1. On March 11, 2020, COVID-19 was first detected in NB, prompting the first set of restrictions, followed by the declaration of a state of emergency on March 19, 2020. These restrictions included the closure of schools, non-essential businesses, and similar limitations. Between March 19, 2020 and May 8, 2020, incidence in NB was lower than the rest of Canada, at approximately 92% fewer cases per 100,000 people [19]. This resulted in the loosening of restrictions into the summer of 2020 that can be expressed as two different types of policies: (i) the reopening of businesses and (ii) an increase in number of recommended social contacts. On May 22, 2020, restrictions on social gatherings were relaxed, low contact organized sports were reintroduced, and personal services were allowed to reopen [20, 21].

**Table 1:** Policy dates and characteristics.

Date	Key Characteristics
**March 11, 2020**	First COVID-19 case detected
	Travel restrictions
	State of emergency on March 19, 2020
**May 22, 2020**	Restrictions on social gatherings were relaxed
	Personal services were allowed to reopen
**January 5, 2021**	Reduced the number of social contacts
**July 31, 2021**	All restrictions lifted
**September 24, 2021**	State of emergency reinstated
	Social contacts limited to 20
	Vaccine requirements

Restrictions were reinstated on January 5, 2021 due to an increase in incidence, and were limited to reducing social contacts [22]. July 31, 2021 marked the first day when restrictions were completely removed. The most significant changes on that day included: no restrictions on gatherings or inter-province travel and an end to all mask requirements [23]. A state of emergency was reinstated on September 24, 2021 with another rise in cases [24]. Penalties for non-compliance with COVID-19 related policies include fines no less than $240 and no more than $10,200, plus a 20% *Victims Services Act* surcharge, and a $4.50 administrative fee [25–28].

To estimate the impact of states of emergency, business-centric policies, and social-centric policies, we use previous years’ ED use as control periods and assume they are typical.

Our data on ED use comes from Horizon Health Network, the provincial health care system for English-speaking regions of the province (61% of all ED visits in the province from 2015–16 to 2019–20 [29]). These data contain information on the age, sex, date of visit, facility, health region, and triage level for all ED visitors from 12 hospitals in four of the seven health zones in NB from January 2017 to October 2021. In total we have 1,579,341 visits of 366,331, 359,101, 349,401, 275,831, and 228,677 from the years 2017, 2018, 2019, 2020 and 2021. Ethical approval for this project was granted by the Horizon Health Networks Research Ethics Board (RS #: 2020–2918) on July 19, 2022. In accordance with the Canadian Tri-Council Policy Statement (TCPS 2)–Chapter 5, section D, Article 5.5B, participant consent was not sought for this secondary use, non-identifiable data [30]. Data on COVID-19 cases and vaccines comes from the Government of Canada [31]. R version 4.2.1 was the software used for statistical analysis and the generation of the figures for this study.

### 2.2 Statistical analysis

Our analysis of the impact of policy changes consists of two longitudinal models. The first model is a multiple linear regression model where our ultimate interest is the residuals. We use the natural logarithm of the number of visitors as our dependent variable.


$$log(y_{itj})=\theta F_{i}+\delta D_{t}+\mu M_{j}+\gamma H_{t}+\epsilon_{itj}$$ (1)

We include control variables for facility *F*_*i*_, day-of-week *D*_*t*_, month-of-year *M*_*j*_, and statutory holidays *H*_*t*_ for each facility *i*, day *t*, and month *j*. *ϵ*_*itj*_ is an error term. Model (1) is weighted by the average number of visits at the EDs to ensure our estimates are proportional to changes in the total number of visits.

We fit model (1) for the years 2017–2019 and are interested in how predicted low-acuity ED visits deviate from the usual pattern following pandemic policy changes assuming changes in low-acuity visits during the pandemic were COVID-19 related, in other words, how the residuals in our model behave from March 11, 2020 onwards. We assume a high R^2^ value and a lack of trends in the residuals before COVID-19 indicates our model is capturing the heterogeneity in utilization prior to COVID-19 and shows the link between COVID-19 and changes in low-acuity ED visits. Changing the dependent variable to the number of high-acuity visits allows us to investigate the effects of COVID-19 on ED visits that are less easily deferred.

Our second model is an event study, a quasi-experimental design that allows us to investigate the impact of specific events while controlling for factors including COVID-19 cases and vaccines administered. In model (2), we use the natural logarithm of the number of visitors *log(y*_*ijwt*_*)* at facility *i*, in year *j*, on day-of-week *w*, on day-of-year *t* as our dependent variable.


$$log(y_{ijwt})=\theta_{i}+\lambda_{j}+\delta_{w}+\mu_{t}+\alpha X_{ijwt}+\sum_{\kappa \in K}\beta_{\kappa }D_{ijwt}^{\kappa }+\epsilon_{ijwt}$$ (2)

The number of visitors is dependent on fixed effects for ED facility θ_*i*_, year *λ*_*j*_, day-of-week *δ*_*w*_ and day-of-year *μ*_*t*_, a vector of covariates *X*_*ijwt*_ containing the lag of COVID-19 cases and vaccines administered, a series of variables *D*^*κ*^_*ijwt*_ indicating the day *κ* relative to the COVID-19 outbreak, and an error term *ϵ*_*ijwt*_. Model (2) is weighted by the average number of visitors at each ED for the whole period of our data.

In model (2), our coefficient of interest is β*κ*. It represents the additional effect of the policy change on the number of visitors for the day. We run model (2) to evaluate the effect of the policy changes around March 11, 2020, May 22, 2020, January 5, 2021, July 31, 2021, and September 24, 2021. From this model we estimate the time it takes to return to 50% of previous ED visit levels, which we call the “half-life” of a policy.

## 3. Results

### 3.1 Summary statistics

Table 2 provides summary statistics for the number of visitors, their acuity level, sex, and age by pre and during COVID-19. The average daily visitors during COVID-19 decrease by 24% from the pre COVID-19. The percentage of low-acuity visits decreases by 5 percentage points after the start of COVID-19.

**Table 2:** Summary statistics for ED visits pre and post COVID-19, 2017–2020.

	Pre COVID	During COVID
**average daily visitors**	81.67	62.18
**median daily visitors**	74	53
**standard deviation**	45.15	40.29
**low acuity (%)**	56.43	51.24
**high acuity (%)**	42.98	48.39
**male (%)**	45.85	46.71
**female (%)**	54.15	53.29
**ages 14 and under (%)**	14.78	10.08
**ages 15 to 64 (%)**	62.62	64.57
**ages 65 and over (%)**	22.60	25.35

Age and sex are approximately the same across Table 2, with a slight drop in the proportion female and in the youngest age group during COVID-19.

### 3.2 Raw data time trends

**Fig 1:**
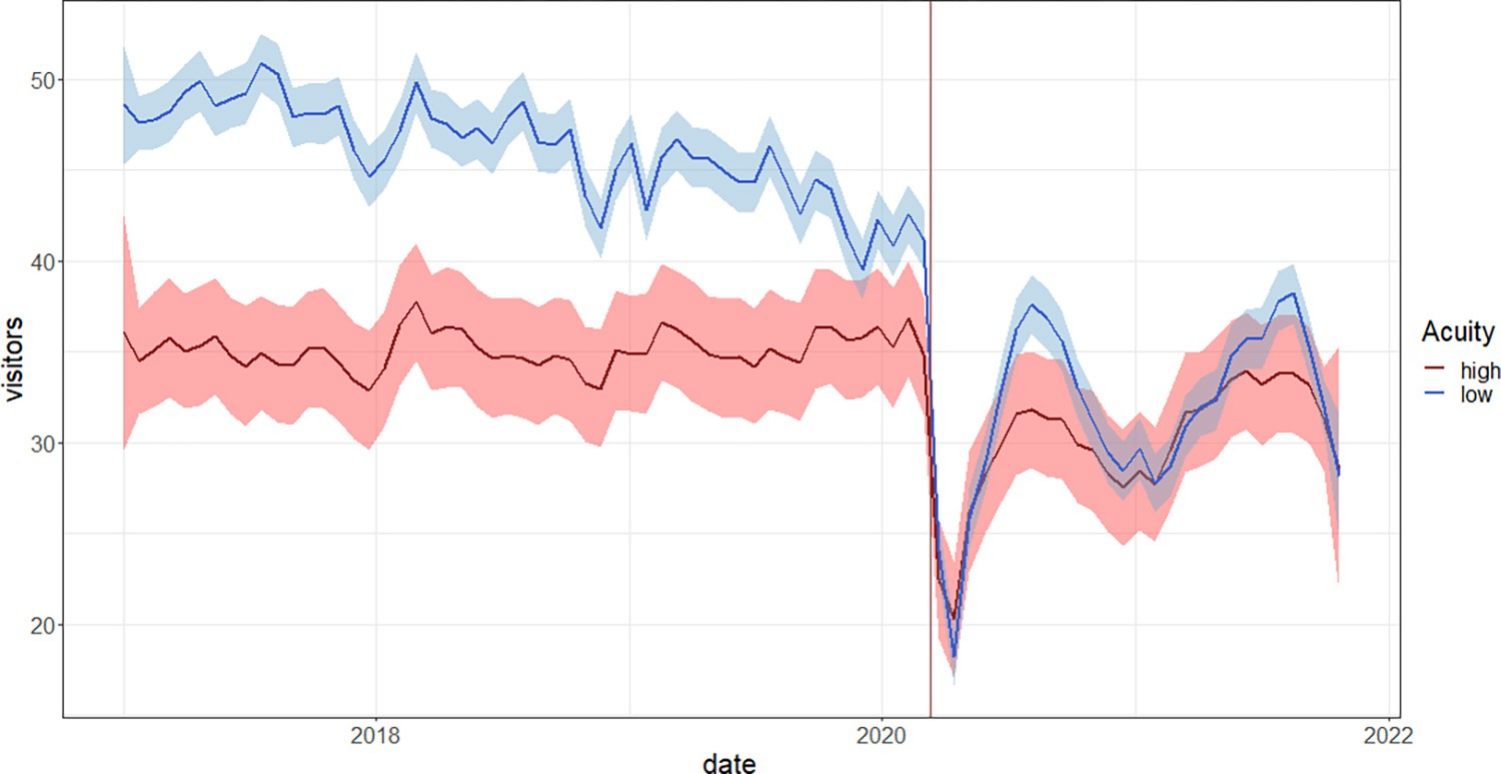
Visitors over time by acuity level.

Fig 1 shows the trend in ED visits per day by level of acuity. The number of low-acuity visitors declines by a larger magnitude than high-acuity visitors. Numbers of daily low-acuity visits are higher than high-acuity visits in almost every period of our sample except for a brief window after the first restrictions were introduced.

### 3.3 Residual model

**Fig 2:**
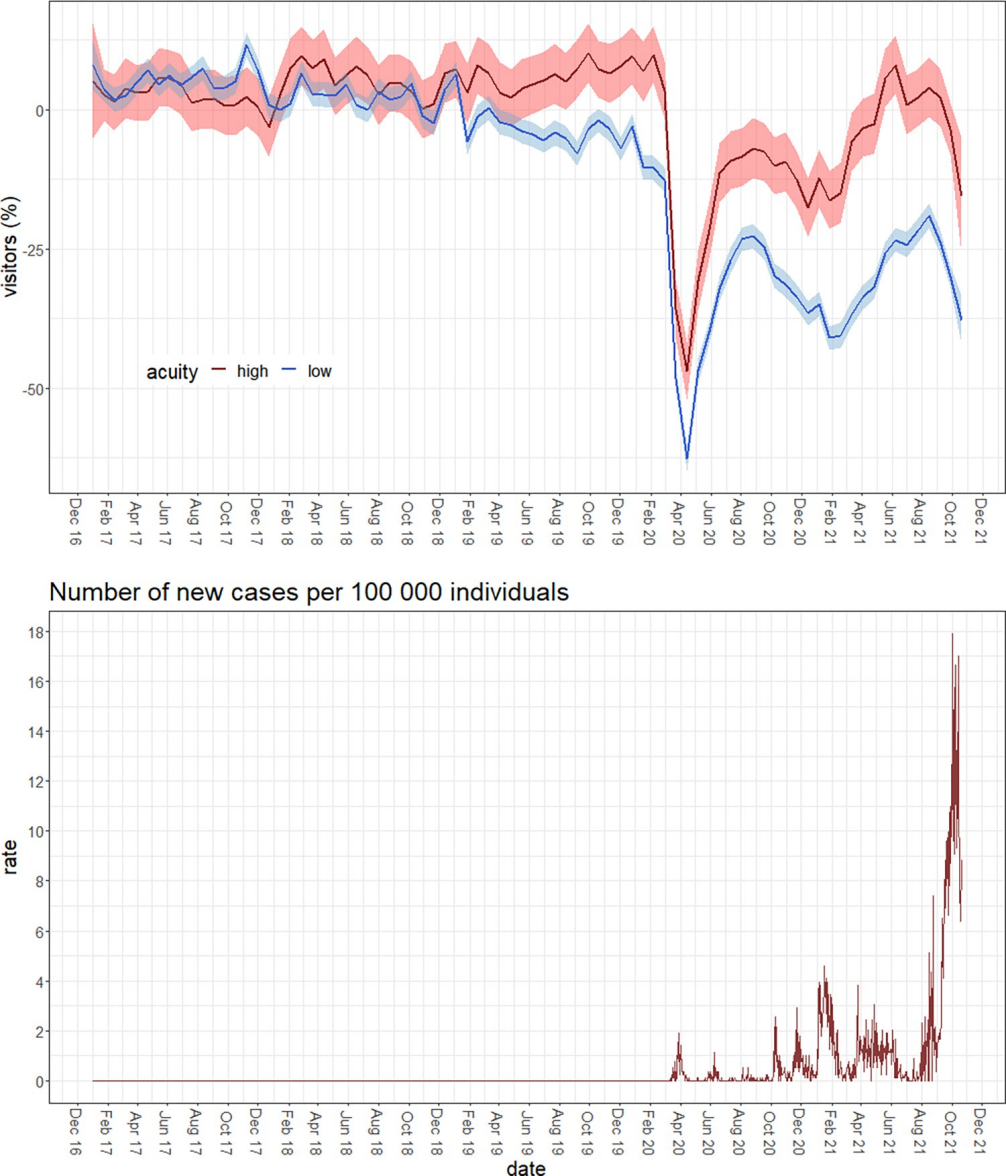
Residuals over time by acuity level.

We estimate model (1) for visitors by acuity level. The residuals from this regression represent the difference between the actual and predicted number of visitors for a given day. In Fig 2 we plot the residuals over time with a 95% confidence interval. Immediately following March 11, 2020, the residuals indicate a 60% drop in ED visits compared with regular levels for both levels of acuity before gradually recovering over the next 5 months. The number of low-acuity visitors recovers to around 15% below original rates, the high-acuity visitors increase to around 30% below original rates before dipping again in the winter. In 2021, the number of low-acuity visits increase to around 20% below original rates in August then decline to around 40% below original rates in December. High-acuity visitors increase to a peak in June before declining to roughly 20% below original rates in December. The R^2^ for this regression is 0.915, meaning that model (1) captures around 92% of the variation in ED visits.

In the second panel, the number of cases of COVID-19 per 100,000 individuals in NB is shown. Until October 2020, the number of cases per 100,000 individuals stays within the zero to two range. In August 2021, cases per 100,000 increases to a peak of around 16 followed by a decline between October 2021 and December 2021.

### 3.3 Event study

**Fig 3:**
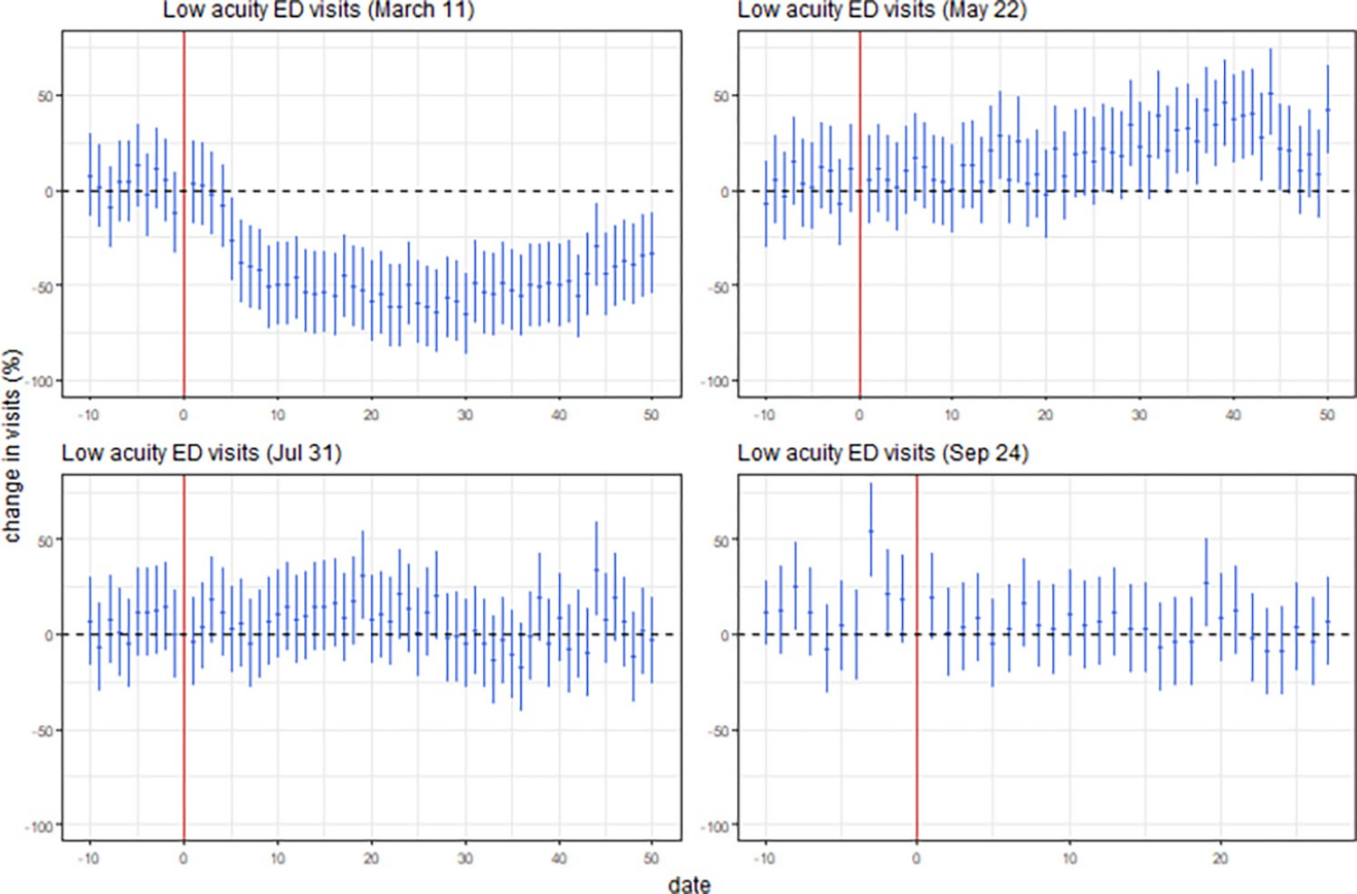
Low acuity ED visits (event study).

Regression results from model (2) are presented in Fig 3. Fig 3 shows trends in low-acuity ED visits from the 4 main events we focus on, state of emergency, travel restrictions, and initially detected case (March 11, 2020), relaxed social restrictions (May 22, 2020), all restrictions lifted (July 31, 2021), and state of emergency reinstated and tightened social restrictions (September 24, 2021). Plotting these regressions with a 95% confidence interval shows the effects of the policy changes on low-acuity ED utilization in percentage terms over time. We do not observe a pre-trend in any of our plots except for the event on January 5, 2021, indicating the validity of the effect of the treatment for most of our estimates. The pre-trend in the January 5, 2021, event suggests unobserved heterogeneity could be confounding our estimates so we do not present results for this event.

For the March 11, 2020 event, low-acuity ED visits decline to a minimum around 20 and 30 days after the event before increasing slightly for the remaining 20 days. Visitors with low scores are estimated to decrease by 66% and stayed low for around 30 days. The coefficient for the lag of COVID-19 cases (-0.18%) indicate a decrease in expected visits with each additional COVID-19 case.

The relaxation of restrictions on May 22, 2020 have mild effects. Reducing restrictions on May 22, 2020 is associated with a gradual increase in ED utilization for visits to approximately 50% for around 44 days. After this increase, visits return to pre-policy levels within a week. The changes are statistically insignificant in the short term. For the remaining 2 events (July 31, 2021, and September 24, 2021) there is no observed change in trends for ED visits.

Results from model (2) are summarized in Table 3. Table 3 shows the approximate maximum impact of the policy, the time it takes for the policy to reach its maximum impact, and to decay back to half of the maximum magnitude (the “half-life”).

**Table 3:** Event study summary.

Event	Days until peak impact	Peak impact (%)	Days until half-life
**State of emergency, travel restrictions, and initially detected case (March 11, 2020)**	30	-66	14
**Relaxed social restrictions (May 22, 2020)**	44	51	1
**All restrictions lifted (July 31, 2021)**	19	25	5
**State of emergency reinstated and tightened social restrictions (September 24, 2021)**	NA	NA	NA

For the initial event, when COVID-19 was first detected in the province, and the subsequent policies introduced following the case, we observe a 66% reduction in low-acuity ED visits after 30 days with a half-life of 14 days, meaning that it takes 14 days for visits to return to half of the magnitude of the maximum effect of the event after the maximum impact has occurred.

When social restrictions are relaxed, ED visits increased by 51% after 44 days with a half-life of 1 day. After all restrictions are lifted, we find a 25% increase in low-acuity ED visits after 19 days with half-life of 5 days. After the state of emergency is reinstated and social restrictions are tightened we do not observe any discernible change in the trends of ED visits.

## 4. Discussion

We find some evidence of policy changes influencing infection-control behaviour in a jurisdiction with a low prevalence of COVID-19 cases. The impact on infection control behaviour is especially pronounced when policies are enacted early. The decrease in low-acuity ED visits following from March 11, 2020 restrictions only begins to decay after 30 days; reaching a minimum of around 66% below original rates and returning to roughly 25% below original rates after 50 days. Other secular factors may play a role in estimating the policy’s impact. For example, the first recorded COVID-19 case in NB occurred March 11, 2020 and other policies were implemented progressively that week.

Relaxing social restrictions on May 22, 2020 has a gradually increasing effect on low-acuity visits to approximately 51% above original rates before dropping to close to original rates within 1 to 3 days. These results provide evidence that infection-control behaviours react to social-centric policies. In other words, the more individuals feel like they are behaving normally in their social circles, the more they are likely to revert to their usual pre-pandemic health care use, like going to the ED for low acuity problems.

Findings from policy changes later in the pandemic indicate little to no behavioural response. Lifting all restrictions and reinstating the state of emergency did little to change behaviour later in the pandemic, indicative of people adjusting or accepting the risk of COVID-19. This adjustment has been called “pandemic fatigue” and suggests policies lose effectiveness over time with more changes in restrictions. The maximum policy impact and persistence of the impact depend on what policies came before. We find a negative relationship between COVID-19 cases and the number of ED visits, which suggests people respond to COVID-19 risk in the community even as policies are relaxed. In other words, a reaction from one policy intervention is not generalizable to that same intervention at another time, which is sometimes called “the Lucas critique” by policy researchers [18].

Our results provide policymakers with information on what behavioural changes to expect from future policies. Results from May 22, 2020 provide some of the first evidence of the effects of relaxing restrictions, with there being no effect found from the relaxing of restrictions on non-essential businesses and a mild effect from restrictions on social gatherings and personal services. Our study finds no impacts of later policy changes where restrictions are completely removed or when a state of emergency is reinstated. This finding shows individuals may tire of restrictions and their volatility, and policies lose effectiveness over time.

If policymakers wish to influence infection-control behaviour, case numbers should continue to be measured and publicly reported. We do not observe any policies that were strong enough to influence ED visits towards the end of the period, implying the reaction to early policies are not predictive of later policies. Even when policies are ineffective, providing information on the number of cases in the region could still influence infection-control behaviour.

One of the main limitations of our analysis is that it is based on a small population located in one province of Canada. This constrains both the representativeness of our study and the number of policy changes we have available to analyze. Among the policies we analyze, there is potential for overlapping effects and earlier policies are not identical to later policies. Although we control for COVID-19 cases, the first cases may be more influential than later cases, meaning our static estimate of the role of COVID-19 case prevalence would be misleading for early cases, i.e., behavioural reactions to COVID-19 prevalence can change over time, just like behavioural reactions to policy changes. Another potential for overlapping effects comes from events influencing each other. To mitigate the risk of this, we choose events that are temporally distant from each other. The lack of pre-trends in many of our event studies indicate the effect of overlapping events is minimal. The use of remote visits as a substitute for ED visits is another factor that may confound our estimates. In New Brunswick visitors have the options to remotely meet with their physicians through telehealth or, starting in February 2020, virtually through evisitnb [32]. Although the presence of a substitute allows visitors to be flexible with their infection control behaviour, if visitors change their behaviour due to changes in their awareness of a substitute it would bias our estimates.

Our study provides a foundation for potential future research. Using data from a larger jurisdiction with more policy changes would improve generalizability of our findings and provide more information on differences in effectiveness between policies. Having identical policy changes that happen at different times in relation to the start of the pandemic would provide more insight into changes in the effectiveness of policy changes depending on the time they happen and in relation to the number of policies that have been administered. Additionally, the use of data on remote visits could address the potential use of remote visits as a substitute for in person ED visits, or the role of remote visits generating trips to the ED.

Our study provides estimates of the effects of restrictions on infection-control behavioural trends when restrictions are tightened and relaxed. Although it is based on a small population, the policies we analyze are universal and evidence from other countries will add to this body of research.

## Supporting information

S1 File Residuals regression by triage score (1–2). (PDF)
