# Supplementary material for: The effectiveness and decay of public health policy actions on infection-control behaviour in the general public: Evidence from a low-COVID prevalence jurisdiction
Source: PLoS One. 2023 Aug 30;18(8):e0283711. doi: 10.1371/journal.pone.0283711 (PMC10468032; doi:10.1371/journal.pone.0283711)
Supplement: S1 File — (PDF) [file pone.0283711.s001.pdf]

Table 1: Residuals Regression by Triage Score (1 — 2)

	log(triage123)	log(triage45)
	(1)	(2)
Tuesday	−0.077*** (0.018)	−0.070*** (0.008)
Wednesday	−0.092*** (0.018)	−0.092*** (0.008)
Thursday	−0.101*** (0.018)	−0.096*** (0.008)
Friday	−0.102*** (0.018)	−0.087*** (0.008)
Saturday	−0.152*** (0.018)	−0.135*** (0.008)
Sunday	−0.131*** (0.018)	−0.103*** (0.008)
February	0.066*** (0.024)	0.034*** (0.010)
March	0.056** (0.023)	0.039*** (0.010)
April	0.039* (0.023)	0.033*** (0.010)
May	0.040* (0.023)	0.025** (0.010)
June	−0.008 (0.023)	0.022** (0.010)
July	0.023 (0.023)	0.062*** (0.010)
August	0.004 (0.023)	0.030*** (0.010)
September	0.027 (0.023)	0.017* (0.010)
October	0.023 (0.023)	0.002 (0.010)
November	0.000 (0.023)	−0.050*** (0.010)
December	0.000 (0.023)	−0.042*** (0.010)
New Years Day	−0.009 (0.091)	−0.058 (0.038)
Family Day	−0.009 (0.092)	0.078** (0.039)
Good Friday	−0.069 (0.092)	0.053 (0.038)
Victoria Day	−0.076 (0.092)	0.026 (0.039)
Canada Day	−0.183** (0.091)	−0.085** (0.038)
New Brunswick Day	−0.101 (0.092)	0.071* (0.039)
Labour Day	−0.193** (0.092)	−0.047 (0.039)
Remembrence Day	−0.074 (0.092)	0.024 (0.039)
Christmas Day	−0.414*** (0.092)	−0.365*** (0.039)
Boxing Day	0.089 (0.092)	0.223*** (0.039)
EDFacility1	−1.345*** (0.023)	−0.404*** (0.010)
EDFacility2	0.340*** (0.015)	0.179*** (0.006)
EDFacility3	−3.096*** (0.039)	−1.408*** (0.015)
EDFacility4	−1.808*** (0.025)	−0.532*** (0.010)
EDFacility5	−0.132*** (0.018)	−0.137*** (0.007)
EDFacility6	0.589*** (0.015)	0.061*** (0.006)
EDFacility7	−0.986*** (0.018)	0.280*** (0.007)
EDFacility8	−2.315*** (0.024)	−0.376*** (0.010)
EDFacility9	−0.832*** (0.019)	0.051*** (0.008)
EDFacility10	3.972*** (0.022)	4.015*** (0.009)
Observations	12,964	13,112
R^2	0.714	0.598
Adjusted R^2	0.713	0.597
Residual Std. Error	4.866 (df = 12927)	2.050 (df = 13075)
F Statistic	894.620*** (df = 36; 12927)	539.576*** (df = 36; 13075)
Notes:	***Significant at the 1 percent level.	
	**Significant at the 5 percent level.	
	*Significant at the 10 percent level.	

Table 2: March 11 - Event Study

	log(triage45)	log(triage123)
	(1)	(2)
DkEventlower_bin	0.014 (0.075)	0.133 (0.096)
DkEvent-10	0.073 (0.105)	0.084 (0.135)
DkEvent-9	0.017 (0.105)	0.060 (0.135)

DkEvent-8	−0.097 (0.105)	0.018 (0.135)
DkEvent-7	0.044 (0.104)	0.230* (0.135)
DkEvent-6	0.043 (0.105)	0.099 (0.135)
DkEvent-5	0.121 (0.105)	0.076 (0.135)
DkEvent-4	−0.028 (0.105)	0.061 (0.136)
DkEvent-3	0.106 (0.105)	0.155 (0.135)
DkEvent-2	0.050 (0.105)	0.086 (0.135)
DkEvent-1	−0.130 (0.105)	−0.052 (0.135)
DkEvent1	0.037 (0.105)	−0.058 (0.135)
DkEvent2	0.028 (0.105)	−0.206 (0.135)
DkEvent3	−0.022 (0.105)	−0.035 (0.135)
DkEvent4	−0.090 (0.105)	−0.332** (0.135)
DkEvent5	−0.305*** (0.105)	−0.404*** (0.135)
DkEvent6	−0.480*** (0.105)	−0.624*** (0.136)
DkEvent7	−0.520*** (0.104)	−0.355*** (0.135)
DkEvent8	−0.545*** (0.105)	−0.451*** (0.135)
DkEvent9	−0.718*** (0.105)	−0.599*** (0.135)
DkEvent10	−0.684*** (0.105)	−0.532*** (0.135)
DkEvent11	−0.684*** (0.105)	−0.509*** (0.135)
DkEvent12	−0.624*** (0.105)	−0.751*** (0.135)
DkEvent13	−0.763*** (0.105)	−0.717*** (0.135)
DkEvent14	−0.787*** (0.104)	−0.483*** (0.135)
DkEvent15	−0.774*** (0.105)	−0.620*** (0.135)
DkEvent16	−0.804*** (0.105)	−0.569*** (0.135)
DkEvent17	−0.606*** (0.105)	−0.515*** (0.135)
DkEvent18	−0.708*** (0.105)	−0.500*** (0.137)
DkEvent19	−0.745*** (0.105)	−0.775*** (0.135)
DkEvent20	−0.886*** (0.105)	−0.831*** (0.136)
DkEvent21	−0.783*** (0.105)	−0.898*** (0.135)
DkEvent22	−0.945*** (0.105)	−0.653*** (0.135)
DkEvent23	−0.947*** (0.105)	−0.629*** (0.135)
DkEvent24	−0.682*** (0.105)	−0.755*** (0.135)
DkEvent25	−0.899*** (0.105)	−0.402*** (0.135)
DkEvent26	−0.951*** (0.105)	−0.689*** (0.135)
DkEvent27	−1.024*** (0.105)	−0.801*** (0.135)
DkEvent28	−0.837*** (0.104)	−0.558*** (0.135)

DkEvent29	−0.886*** (0.105)	−0.768*** (0.135)
DkEvent30	−1.065*** (0.105)	−0.683*** (0.135)
DkEvent31	−0.664*** (0.105)	−0.616*** (0.135)
DkEvent32	−0.776*** (0.105)	−0.659*** (0.135)
DkEvent33	−0.802*** (0.105)	−0.584*** (0.135)
DkEvent34	−0.676*** (0.105)	−0.508*** (0.135)
DkEvent35	−0.755*** (0.104)	−0.664*** (0.135)
DkEvent36	−0.813*** (0.105)	−0.553*** (0.135)
DkEvent37	−0.700*** (0.105)	−0.362*** (0.135)
DkEvent38	−0.707*** (0.105)	−0.632*** (0.135)
DkEvent39	−0.673*** (0.105)	−0.458*** (0.135)
DkEvent40	−0.695*** (0.105)	−0.546*** (0.135)
DkEvent41	−0.659*** (0.105)	−0.451*** (0.135)
DkEvent42	−0.823*** (0.104)	−0.401*** (0.135)
DkEvent43	−0.588*** (0.105)	−0.442*** (0.136)
DkEvent44	−0.346*** (0.105)	−0.392*** (0.135)
DkEvent45	−0.585*** (0.105)	−0.280** (0.135)
DkEvent46	−0.517*** (0.105)	−0.534*** (0.135)
DkEvent47	−0.469*** (0.105)	−0.526*** (0.135)
DkEvent48	−0.497*** (0.105)	−0.442*** (0.135)
DkEvent49	−0.428*** (0.104)	−0.529*** (0.135)
DkEvent50	−0.405*** (0.105)	−0.371*** (0.135)
DkEventupper_bin	−0.197*** (0.074)	−0.104 (0.096)
lag(DailyTotals, n = 1)	−0.002*** (0.000)	−0.001*** (0.000)
TotalVaccinated	0.000*** (0.000)	0.000*** (0.000)
EDFacility1	0.646*** (0.008)	1.782*** (0.011)
EDFacility2	−1.128*** (0.014)	−1.607*** (0.019)
EDFacility3	−0.211*** (0.011)	−0.376*** (0.014)
EDFacility4	0.206*** (0.009)	1.305*** (0.011)
EDFacility5	0.452*** (0.008)	2.022*** (0.011)
EDFacility6	0.625*** (0.009)	0.378*** (0.011)
EDFacility7	−0.017 (0.010)	−0.841*** (0.014)
EDFacility8	0.523*** (0.008)	2.135*** (0.010)
EDFacility9	0.495*** (0.009)	0.430*** (0.012)
EDFacility10	0.188*** (0.010)	0.232*** (0.012)
EDFacility11	0.305*** (0.009)	0.682*** (0.012)

as.factor(yr)2018	-0.041^{***} (0.005)	0.019^{***} (0.006)
as.factor(yr)2019	-0.116^{***} (0.005)	0.020^{***} (0.006)
as.factor(yr)2020	-0.186^{***} (0.010)	0.050^{***} (0.013)
as.factor(yr)2021	-0.246^{***} (0.014)	0.102^{***} (0.018)
as.factor(dow)monday	0.088^{***} (0.006)	0.089^{***} (0.008)
as.factor(dow)saturday	-0.060^{***} (0.006)	-0.060^{***} (0.008)
as.factor(dow)sunday	-0.043^{***} (0.006)	-0.050^{***} (0.008)
as.factor(dow)thursday	0.002 (0.006)	0.007 (0.008)
as.factor(dow)tuesday	0.019^{***} (0.006)	0.024^{***} (0.008)
as.factor(dow)wednesday	0.003 (0.006)	0.017^{**} (0.008)
as.factor(doy)01-02	0.197^{***} (0.042)	0.029 (0.054)
as.factor(doy)01-03	0.140^{***} (0.042)	0.098^{*} (0.054)
as.factor(doy)01-04	0.043 (0.042)	−0.028 (0.054)
as.factor(doy)01-05	0.088^{**} (0.042)	0.009 (0.054)
as.factor(doy)01-06	0.095^{**} (0.042)	−0.032 (0.054)
as.factor(doy)01-07	0.022 (0.042)	−0.005 (0.054)
as.factor(doy)01-08	−0.005 (0.042)	-0.119^{**} (0.054)
as.factor(doy)01-09	−0.017 (0.042)	−0.070 (0.054)
as.factor(doy)01-10	−0.001 (0.042)	−0.085 (0.054)
as.factor(doy)01-11	0.028 (0.042)	−0.069 (0.054)
as.factor(doy)01-12	−0.033 (0.042)	−0.066 (0.054)
as.factor(doy)01-13	0.017 (0.042)	-0.099^{*} (0.054)
as.factor(doy)01-14	−0.023 (0.042)	−0.081 (0.054)
as.factor(doy)01-15	0.018 (0.042)	−0.053 (0.054)
as.factor(doy)01-16	−0.039 (0.042)	−0.026 (0.054)
as.factor(doy)01-17	−0.023 (0.042)	-0.116^{**} (0.054)
as.factor(doy)01-18	0.017 (0.042)	−0.046 (0.054)
as.factor(doy)01-19	−0.015 (0.042)	−0.079 (0.054)
as.factor(doy)01-20	-0.096^{**} (0.042)	-0.201^{***} (0.054)
as.factor(doy)01-21	−0.035 (0.042)	−0.045 (0.054)
as.factor(doy)01-22	0.039 (0.042)	−0.035 (0.054)
as.factor(doy)01-23	−0.020 (0.042)	−0.086 (0.054)
as.factor(doy)01-24	-0.085^{**} (0.042)	−0.084 (0.054)
as.factor(doy)01-25	0.006 (0.042)	−0.018 (0.054)
as.factor(doy)01-26	0.069 (0.042)	−0.066 (0.054)
as.factor(doy)01-27	0.073^{*} (0.042)	0.043 (0.054)

as.factor(doy)01-28	0.076* (0.042)	0.011 (0.054)
as.factor(doy)01-29	0.076* (0.042)	0.004 (0.054)
as.factor(doy)01-30	0.003 (0.042)	0.012 (0.054)
as.factor(doy)01-31	0.032 (0.042)	−0.034 (0.054)
as.factor(doy)02-01	0.087** (0.042)	−0.024 (0.054)
as.factor(doy)02-02	−0.069* (0.042)	−0.132** (0.054)
as.factor(doy)02-03	0.078* (0.042)	0.003 (0.054)
as.factor(doy)02-04	0.071* (0.042)	0.030 (0.054)
as.factor(doy)02-05	0.078* (0.042)	0.086 (0.054)
as.factor(doy)02-06	0.055 (0.042)	0.037 (0.054)
as.factor(doy)02-07	−0.014 (0.042)	−0.046 (0.054)
as.factor(doy)02-08	−0.007 (0.042)	−0.040 (0.054)
as.factor(doy)02-09	0.077* (0.042)	−0.015 (0.054)
as.factor(doy)02-10	−0.012 (0.042)	−0.029 (0.054)
as.factor(doy)02-11	−0.011 (0.042)	−0.019 (0.054)
as.factor(doy)02-12	0.126*** (0.042)	0.039 (0.054)
as.factor(doy)02-13	−0.239*** (0.042)	−0.278*** (0.054)
as.factor(doy)02-14	0.015 (0.042)	0.047 (0.054)
as.factor(doy)02-15	0.081* (0.042)	0.000 (0.054)
as.factor(doy)02-16	−0.063 (0.042)	−0.068 (0.054)
as.factor(doy)02-17	0.077* (0.042)	0.043 (0.054)
as.factor(doy)02-18	0.126*** (0.042)	0.073 (0.054)
as.factor(doy)02-19	0.141*** (0.042)	0.068 (0.054)
as.factor(doy)02-20	0.132*** (0.042)	0.050 (0.054)
as.factor(doy)02-21	0.056 (0.042)	0.065 (0.054)
as.factor(doy)02-22	0.093** (0.042)	0.071 (0.054)
as.factor(doy)02-23	0.098** (0.042)	0.038 (0.054)
as.factor(doy)02-24	0.059 (0.042)	0.075 (0.054)
as.factor(doy)02-25	0.086** (0.042)	0.040 (0.054)
as.factor(doy)02-26	0.080* (0.042)	0.033 (0.054)
as.factor(doy)02-27	0.034 (0.042)	−0.049 (0.054)
as.factor(doy)02-28	0.036 (0.042)	0.078 (0.054)
as.factor(doy)02-29	0.079 (0.073)	0.019 (0.094)
as.factor(doy)03-01	0.064 (0.044)	−0.009 (0.057)
as.factor(doy)03-02	0.005 (0.045)	0.036 (0.057)
as.factor(doy)03-03	0.139*** (0.044)	0.052 (0.057)

as.factor(doy)03-04	0.015 (0.045)	−0.055 (0.057)
as.factor(doy)03-05	0.059 (0.044)	0.053 (0.057)
as.factor(doy)03-06	0.053 (0.044)	0.028 (0.057)
as.factor(doy)03-07	0.063 (0.044)	0.023 (0.057)
as.factor(doy)03-08	0.019 (0.044)	−0.089 (0.057)
as.factor(doy)03-09	0.087* (0.045)	0.070 (0.057)
as.factor(doy)03-10	0.073* (0.044)	0.092 (0.057)
as.factor(doy)03-11	0.044 (0.045)	0.055 (0.057)
as.factor(doy)03-12	0.103** (0.044)	0.103* (0.057)
as.factor(doy)03-13	0.091** (0.044)	0.026 (0.057)
as.factor(doy)03-14	−0.041 (0.044)	−0.042 (0.057)
as.factor(doy)03-15	0.021 (0.044)	0.062 (0.057)
as.factor(doy)03-16	0.078* (0.045)	0.082 (0.057)
as.factor(doy)03-17	0.052 (0.045)	0.122** (0.057)
as.factor(doy)03-18	0.096** (0.045)	0.041 (0.057)
as.factor(doy)03-19	0.079* (0.044)	0.044 (0.057)
as.factor(doy)03-20	0.051 (0.044)	0.010 (0.057)
as.factor(doy)03-21	0.111** (0.045)	−0.003 (0.057)
as.factor(doy)03-22	0.010 (0.044)	−0.047 (0.057)
as.factor(doy)03-23	0.075* (0.045)	0.016 (0.057)
as.factor(doy)03-24	0.108** (0.044)	0.038 (0.057)
as.factor(doy)03-25	0.097** (0.045)	0.041 (0.057)
as.factor(doy)03-26	0.114** (0.044)	0.029 (0.057)
as.factor(doy)03-27	0.044 (0.044)	−0.045 (0.057)
as.factor(doy)03-28	0.011 (0.044)	−0.010 (0.057)
as.factor(doy)03-29	0.063 (0.044)	0.071 (0.057)
as.factor(doy)03-30	0.075* (0.045)	0.003 (0.057)
as.factor(doy)03-31	0.108** (0.044)	0.056 (0.057)
as.factor(doy)04-01	0.080* (0.045)	0.034 (0.058)
as.factor(doy)04-02	0.067 (0.044)	0.033 (0.058)
as.factor(doy)04-03	0.112** (0.044)	−0.045 (0.057)
as.factor(doy)04-04	−0.003 (0.044)	−0.009 (0.057)
as.factor(doy)04-05	0.050 (0.044)	0.035 (0.057)
as.factor(doy)04-06	0.068 (0.045)	0.033 (0.057)
as.factor(doy)04-07	0.155*** (0.044)	0.047 (0.057)
as.factor(doy)04-08	−0.031 (0.045)	0.008 (0.057)

as.factor(doy)04-09	0.069 (0.044)	0.019 (0.057)
as.factor(doy)04-10	0.078* (0.045)	0.046 (0.057)
as.factor(doy)04-11	0.080* (0.045)	0.020 (0.057)
as.factor(doy)04-12	0.051 (0.045)	−0.016 (0.058)
as.factor(doy)04-13	0.058 (0.045)	0.032 (0.057)
as.factor(doy)04-14	0.070 (0.044)	−0.016 (0.057)
as.factor(doy)04-15	0.081* (0.045)	0.069 (0.057)
as.factor(doy)04-16	0.028 (0.044)	−0.006 (0.057)
as.factor(doy)04-17	0.062 (0.044)	−0.038 (0.057)
as.factor(doy)04-18	0.077* (0.044)	0.000 (0.057)
as.factor(doy)04-19	0.060 (0.044)	0.015 (0.057)
as.factor(doy)04-20	0.033 (0.045)	0.027 (0.057)
as.factor(doy)04-21	0.102** (0.044)	0.004 (0.057)
as.factor(doy)04-22	0.104** (0.045)	0.022 (0.057)
as.factor(doy)04-23	0.084* (0.044)	0.027 (0.057)
as.factor(doy)04-24	0.017 (0.044)	−0.018 (0.057)
as.factor(doy)04-25	0.032 (0.044)	−0.021 (0.057)
as.factor(doy)04-26	0.042 (0.044)	0.023 (0.057)
as.factor(doy)04-27	0.063 (0.045)	0.033 (0.057)
as.factor(doy)04-28	0.005 (0.044)	−0.017 (0.057)
as.factor(doy)04-29	0.100** (0.045)	0.052 (0.057)
as.factor(doy)04-30	0.075* (0.044)	−0.028 (0.057)
as.factor(doy)05-01	0.027 (0.042)	−0.055 (0.054)
as.factor(doy)05-02	0.023 (0.042)	−0.053 (0.054)
as.factor(doy)05-03	0.075* (0.042)	0.057 (0.054)
as.factor(doy)05-04	0.031 (0.042)	−0.010 (0.054)
as.factor(doy)05-05	0.044 (0.042)	−0.002 (0.054)
as.factor(doy)05-06	0.007 (0.042)	0.023 (0.054)
as.factor(doy)05-07	0.050 (0.042)	−0.008 (0.054)
as.factor(doy)05-08	0.004 (0.042)	−0.040 (0.054)
as.factor(doy)05-09	−0.082* (0.042)	−0.073 (0.054)
as.factor(doy)05-10	−0.001 (0.042)	0.007 (0.054)
as.factor(doy)05-11	0.002 (0.042)	−0.008 (0.054)
as.factor(doy)05-12	0.024 (0.042)	−0.078 (0.054)
as.factor(doy)05-13	−0.028 (0.042)	0.011 (0.054)
as.factor(doy)05-14	0.010 (0.042)	−0.078 (0.054)

as.factor(doy)05-15	0.003 (0.042)	−0.008 (0.054)
as.factor(doy)05-16	0.057 (0.042)	−0.038 (0.054)
as.factor(doy)05-17	0.011 (0.042)	−0.021 (0.054)
as.factor(doy)05-18	0.013 (0.042)	−0.006 (0.054)
as.factor(doy)05-19	0.105** (0.042)	−0.019 (0.054)
as.factor(doy)05-20	0.094** (0.042)	−0.004 (0.054)
as.factor(doy)05-21	0.063 (0.042)	0.007 (0.054)
as.factor(doy)05-22	0.042 (0.042)	−0.059 (0.054)
as.factor(doy)05-23	0.081* (0.042)	0.024 (0.054)
as.factor(doy)05-24	0.013 (0.042)	0.008 (0.054)
as.factor(doy)05-25	0.083** (0.042)	0.013 (0.054)
as.factor(doy)05-26	0.083** (0.042)	−0.017 (0.054)
as.factor(doy)05-27	0.039 (0.042)	0.032 (0.054)
as.factor(doy)05-28	0.081* (0.042)	−0.001 (0.054)
as.factor(doy)05-29	0.039 (0.042)	−0.004 (0.054)
as.factor(doy)05-30	0.021 (0.042)	−0.010 (0.054)
as.factor(doy)05-31	0.000 (0.042)	−0.022 (0.054)
as.factor(doy)06-01	0.016 (0.042)	−0.013 (0.054)
as.factor(doy)06-02	0.045 (0.042)	−0.048 (0.054)
as.factor(doy)06-03	0.066 (0.042)	0.037 (0.054)
as.factor(doy)06-04	0.094** (0.042)	−0.025 (0.054)
as.factor(doy)06-05	0.053 (0.042)	−0.004 (0.054)
as.factor(doy)06-06	0.090** (0.042)	0.007 (0.054)
as.factor(doy)06-07	0.019 (0.042)	0.003 (0.054)
as.factor(doy)06-08	0.022 (0.042)	0.008 (0.054)
as.factor(doy)06-09	0.047 (0.042)	−0.019 (0.054)
as.factor(doy)06-10	0.076* (0.042)	0.033 (0.054)
as.factor(doy)06-11	0.059 (0.042)	−0.063 (0.054)
as.factor(doy)06-12	0.039 (0.042)	−0.026 (0.054)
as.factor(doy)06-13	0.063 (0.042)	−0.018 (0.054)
as.factor(doy)06-14	0.044 (0.042)	−0.012 (0.054)
as.factor(doy)06-15	0.089** (0.042)	−0.022 (0.054)
as.factor(doy)06-16	0.046 (0.042)	−0.062 (0.054)
as.factor(doy)06-17	0.033 (0.042)	−0.075 (0.054)
as.factor(doy)06-18	0.066 (0.042)	−0.006 (0.054)
as.factor(doy)06-19	0.103** (0.042)	−0.060 (0.054)

as.factor(doy)06-20	0.038 (0.042)	−0.064 (0.054)
as.factor(doy)06-21	−0.022 (0.042)	−0.011 (0.054)
as.factor(doy)06-22	0.063 (0.042)	−0.040 (0.054)
as.factor(doy)06-23	0.095** (0.042)	−0.029 (0.054)
as.factor(doy)06-24	0.066 (0.042)	−0.056 (0.054)
as.factor(doy)06-25	0.068 (0.042)	−0.000 (0.054)
as.factor(doy)06-26	0.105** (0.042)	−0.034 (0.054)
as.factor(doy)06-27	0.048 (0.042)	−0.032 (0.054)
as.factor(doy)06-28	0.104** (0.042)	−0.060 (0.054)
as.factor(doy)06-29	0.049 (0.042)	−0.037 (0.054)
as.factor(doy)06-30	0.123*** (0.042)	−0.012 (0.054)
as.factor(doy)07-01	0.053 (0.042)	−0.125** (0.054)
as.factor(doy)07-02	0.150*** (0.042)	0.028 (0.054)
as.factor(doy)07-03	0.136*** (0.042)	0.028 (0.054)
as.factor(doy)07-04	0.089** (0.042)	−0.064 (0.054)
as.factor(doy)07-05	0.076* (0.042)	0.016 (0.054)
as.factor(doy)07-06	0.055 (0.042)	−0.033 (0.054)
as.factor(doy)07-07	0.117*** (0.042)	−0.023 (0.054)
as.factor(doy)07-08	0.087** (0.042)	0.023 (0.054)
as.factor(doy)07-09	0.050 (0.042)	0.038 (0.054)
as.factor(doy)07-10	0.104** (0.042)	0.034 (0.054)
as.factor(doy)07-11	0.038 (0.042)	−0.023 (0.054)
as.factor(doy)07-12	0.110*** (0.042)	0.013 (0.054)
as.factor(doy)07-13	0.124*** (0.042)	−0.019 (0.054)
as.factor(doy)07-14	0.128*** (0.042)	−0.017 (0.054)
as.factor(doy)07-15	0.120*** (0.042)	0.002 (0.054)
as.factor(doy)07-16	0.048 (0.042)	0.034 (0.054)
as.factor(doy)07-17	0.041 (0.042)	−0.019 (0.054)
as.factor(doy)07-18	0.063 (0.042)	−0.047 (0.054)
as.factor(doy)07-19	0.053 (0.042)	0.016 (0.054)
as.factor(doy)07-20	0.109*** (0.042)	−0.007 (0.054)
as.factor(doy)07-21	0.108** (0.042)	−0.041 (0.054)
as.factor(doy)07-22	0.151*** (0.042)	−0.003 (0.054)
as.factor(doy)07-23	0.166*** (0.042)	−0.028 (0.054)
as.factor(doy)07-24	0.116*** (0.042)	−0.021 (0.054)
as.factor(doy)07-25	0.110*** (0.042)	0.008 (0.054)

as.factor(doy)07-26	0.126*** (0.042)	0.011 (0.054)
as.factor(doy)07-27	0.112*** (0.042)	−0.012 (0.054)
as.factor(doy)07-28	0.142*** (0.042)	0.033 (0.054)
as.factor(doy)07-29	0.177*** (0.042)	−0.010 (0.054)
as.factor(doy)07-30	0.118*** (0.042)	−0.038 (0.054)
as.factor(doy)07-31	0.125*** (0.042)	−0.013 (0.054)
as.factor(doy)08-01	0.096** (0.042)	−0.094* (0.054)
as.factor(doy)08-02	0.098** (0.042)	−0.008 (0.054)
as.factor(doy)08-03	0.179*** (0.042)	−0.047 (0.054)
as.factor(doy)08-04	0.114*** (0.042)	0.067 (0.054)
as.factor(doy)08-05	0.131*** (0.042)	−0.016 (0.054)
as.factor(doy)08-06	0.166*** (0.042)	−0.001 (0.054)
as.factor(doy)08-07	0.142*** (0.042)	−0.013 (0.054)
as.factor(doy)08-08	0.160*** (0.042)	0.007 (0.054)
as.factor(doy)08-09	0.109*** (0.042)	0.009 (0.054)
as.factor(doy)08-10	0.087** (0.042)	−0.019 (0.054)
as.factor(doy)08-11	0.090** (0.042)	0.020 (0.054)
as.factor(doy)08-12	0.128*** (0.042)	0.012 (0.054)
as.factor(doy)08-13	0.127*** (0.042)	−0.034 (0.054)
as.factor(doy)08-14	0.078* (0.042)	−0.033 (0.054)
as.factor(doy)08-15	0.106** (0.042)	−0.097* (0.054)
as.factor(doy)08-16	0.097** (0.042)	0.011 (0.054)
as.factor(doy)08-17	0.082* (0.042)	−0.022 (0.054)
as.factor(doy)08-18	0.070* (0.042)	0.013 (0.054)
as.factor(doy)08-19	0.102** (0.042)	−0.008 (0.054)
as.factor(doy)08-20	0.071* (0.042)	−0.027 (0.054)
as.factor(doy)08-21	0.051 (0.042)	0.010 (0.054)
as.factor(doy)08-22	0.084** (0.042)	−0.049 (0.054)
as.factor(doy)08-23	0.110*** (0.042)	0.031 (0.054)
as.factor(doy)08-24	0.115*** (0.042)	−0.020 (0.054)
as.factor(doy)08-25	0.053 (0.042)	0.015 (0.054)
as.factor(doy)08-26	0.056 (0.042)	0.002 (0.054)
as.factor(doy)08-27	0.075* (0.042)	0.010 (0.054)
as.factor(doy)08-28	0.014 (0.042)	−0.051 (0.054)
as.factor(doy)08-29	0.028 (0.042)	−0.082 (0.054)
as.factor(doy)08-30	0.033 (0.042)	0.024 (0.054)

as.factor(doy)08-31	0.054 (0.042)	−0.016 (0.054)
as.factor(doy)09-01	0.053 (0.042)	−0.048 (0.054)
as.factor(doy)09-02	0.040 (0.042)	−0.081 (0.054)
as.factor(doy)09-03	0.030 (0.042)	−0.025 (0.054)
as.factor(doy)09-04	0.061 (0.042)	−0.024 (0.054)
as.factor(doy)09-05	0.035 (0.042)	−0.028 (0.054)
as.factor(doy)09-06	0.052 (0.042)	−0.020 (0.054)
as.factor(doy)09-07	0.008 (0.042)	−0.024 (0.054)
as.factor(doy)09-08	0.108** (0.042)	−0.035 (0.054)
as.factor(doy)09-09	0.038 (0.042)	0.020 (0.055)
as.factor(doy)09-10	0.022 (0.042)	−0.001 (0.054)
as.factor(doy)09-11	0.017 (0.042)	−0.002 (0.054)
as.factor(doy)09-12	0.052 (0.042)	0.003 (0.054)
as.factor(doy)09-13	0.080* (0.042)	0.112** (0.054)
as.factor(doy)09-14	0.054 (0.042)	−0.014 (0.054)
as.factor(doy)09-15	0.066 (0.042)	0.039 (0.054)
as.factor(doy)09-16	0.099** (0.042)	0.032 (0.054)
as.factor(doy)09-17	0.102** (0.042)	0.020 (0.054)
as.factor(doy)09-18	0.056 (0.042)	0.057 (0.054)
as.factor(doy)09-19	0.063 (0.042)	−0.027 (0.054)
as.factor(doy)09-20	0.083* (0.042)	0.003 (0.055)
as.factor(doy)09-21	0.068 (0.042)	0.024 (0.054)
as.factor(doy)09-22	0.124*** (0.042)	−0.030 (0.054)
as.factor(doy)09-23	0.061 (0.042)	0.055 (0.054)
as.factor(doy)09-24	0.054 (0.042)	0.069 (0.054)
as.factor(doy)09-25	0.033 (0.042)	0.027 (0.054)
as.factor(doy)09-26	0.092** (0.042)	0.027 (0.054)
as.factor(doy)09-27	0.069 (0.042)	−0.021 (0.054)
as.factor(doy)09-28	0.080* (0.042)	0.023 (0.054)
as.factor(doy)09-29	0.090** (0.042)	−0.009 (0.054)
as.factor(doy)09-30	0.057 (0.042)	−0.008 (0.054)
as.factor(doy)10-01	0.033 (0.042)	0.025 (0.054)
as.factor(doy)10-02	0.049 (0.042)	0.018 (0.054)
as.factor(doy)10-03	0.013 (0.042)	0.040 (0.054)
as.factor(doy)10-04	0.030 (0.042)	0.023 (0.054)
as.factor(doy)10-05	0.070* (0.042)	−0.034 (0.054)

as.factor(doy)10-06	0.043 (0.042)	0.031 (0.054)
as.factor(doy)10-07	0.061 (0.042)	0.024 (0.054)
as.factor(doy)10-08	0.034 (0.042)	−0.009 (0.054)
as.factor(doy)10-09	−0.005 (0.042)	0.001 (0.054)
as.factor(doy)10-10	0.040 (0.042)	0.031 (0.054)
as.factor(doy)10-11	0.008 (0.042)	−0.066 (0.054)
as.factor(doy)10-12	−0.000 (0.042)	−0.023 (0.054)
as.factor(doy)10-13	0.042 (0.042)	0.002 (0.054)
as.factor(doy)10-14	0.091** (0.042)	−0.071 (0.054)
as.factor(doy)10-15	0.089** (0.042)	0.025 (0.054)
as.factor(doy)10-16	0.045 (0.042)	−0.057 (0.054)
as.factor(doy)10-17	−0.078* (0.042)	−0.013 (0.054)
as.factor(doy)10-18	0.011 (0.042)	−0.014 (0.054)
as.factor(doy)10-19	−0.009 (0.042)	−0.025 (0.054)
as.factor(doy)10-20	−0.019 (0.042)	−0.040 (0.054)
as.factor(doy)10-21	0.017 (0.045)	0.013 (0.057)
as.factor(doy)10-22	0.061 (0.045)	0.032 (0.057)
as.factor(doy)10-23	0.021 (0.045)	−0.011 (0.057)
as.factor(doy)10-24	0.047 (0.045)	−0.036 (0.058)
as.factor(doy)10-25	−0.028 (0.045)	−0.029 (0.058)
as.factor(doy)10-26	0.009 (0.045)	−0.006 (0.058)
as.factor(doy)10-27	−0.016 (0.045)	−0.014 (0.057)
as.factor(doy)10-28	−0.039 (0.045)	−0.034 (0.057)
as.factor(doy)10-29	0.048 (0.045)	0.007 (0.057)
as.factor(doy)10-30	0.001 (0.045)	−0.047 (0.057)
as.factor(doy)10-31	−0.083* (0.045)	−0.109* (0.057)
as.factor(doy)11-01	−0.003 (0.045)	0.029 (0.057)
as.factor(doy)11-02	−0.001 (0.045)	−0.006 (0.057)
as.factor(doy)11-03	−0.012 (0.045)	−0.016 (0.057)
as.factor(doy)11-04	−0.012 (0.045)	−0.026 (0.057)
as.factor(doy)11-05	−0.011 (0.045)	−0.018 (0.057)
as.factor(doy)11-06	0.057 (0.045)	0.094 (0.057)
as.factor(doy)11-07	−0.076* (0.045)	−0.036 (0.057)
as.factor(doy)11-08	−0.071 (0.045)	−0.044 (0.057)
as.factor(doy)11-09	0.030 (0.045)	−0.074 (0.057)
as.factor(doy)11-10	0.049 (0.045)	−0.049 (0.057)

as.factor(doy)11-11	−0.012 (0.045)	−0.061 (0.057)
as.factor(doy)11-12	−0.018 (0.045)	0.002 (0.057)
as.factor(doy)11-13	−0.016 (0.045)	−0.018 (0.057)
as.factor(doy)11-14	−0.042 (0.045)	−0.015 (0.058)
as.factor(doy)11-15	−0.028 (0.045)	0.014 (0.058)
as.factor(doy)11-16	−0.068 (0.045)	−0.134** (0.058)
as.factor(doy)11-17	−0.014 (0.045)	0.010 (0.057)
as.factor(doy)11-18	−0.021 (0.045)	−0.024 (0.057)
as.factor(doy)11-19	−0.006 (0.045)	−0.015 (0.057)
as.factor(doy)11-20	−0.025 (0.045)	−0.065 (0.057)
as.factor(doy)11-21	−0.088** (0.045)	−0.061 (0.057)
as.factor(doy)11-22	−0.035 (0.045)	−0.052 (0.057)
as.factor(doy)11-23	−0.073* (0.045)	−0.060 (0.057)
as.factor(doy)11-24	−0.031 (0.045)	−0.036 (0.057)
as.factor(doy)11-25	−0.032 (0.045)	−0.050 (0.057)
as.factor(doy)11-26	0.002 (0.045)	−0.024 (0.057)
as.factor(doy)11-27	0.029 (0.045)	−0.057 (0.057)
as.factor(doy)11-28	−0.085* (0.045)	−0.077 (0.057)
as.factor(doy)11-29	−0.064 (0.045)	−0.052 (0.057)
as.factor(doy)11-30	−0.076* (0.045)	−0.092 (0.057)
as.factor(doy)12-01	−0.029 (0.045)	−0.068 (0.057)
as.factor(doy)12-02	−0.043 (0.045)	−0.054 (0.057)
as.factor(doy)12-03	−0.138*** (0.045)	−0.088 (0.057)
as.factor(doy)12-04	−0.040 (0.045)	0.016 (0.057)
as.factor(doy)12-05	−0.070 (0.045)	−0.062 (0.058)
as.factor(doy)12-06	−0.044 (0.045)	−0.062 (0.057)
as.factor(doy)12-07	−0.073* (0.045)	−0.051 (0.057)
as.factor(doy)12-08	−0.123*** (0.045)	−0.053 (0.057)
as.factor(doy)12-09	−0.091** (0.045)	−0.028 (0.057)
as.factor(doy)12-10	−0.054 (0.045)	−0.018 (0.057)
as.factor(doy)12-11	−0.052 (0.045)	0.055 (0.057)
as.factor(doy)12-12	−0.095** (0.045)	−0.044 (0.057)
as.factor(doy)12-13	−0.152*** (0.045)	−0.074 (0.057)
as.factor(doy)12-14	−0.088** (0.045)	−0.069 (0.057)
as.factor(doy)12-15	−0.071 (0.045)	−0.061 (0.057)
as.factor(doy)12-16	−0.055 (0.045)	−0.054 (0.057)

as.factor(doy)12-17	−0.037 (0.045)	−0.054 (0.057)
as.factor(doy)12-18	−0.059 (0.045)	−0.121** (0.057)
as.factor(doy)12-19	−0.068 (0.045)	−0.143** (0.058)
as.factor(doy)12-20	−0.050 (0.045)	−0.169*** (0.057)
as.factor(doy)12-21	−0.059 (0.045)	−0.031 (0.058)
as.factor(doy)12-22	0.027 (0.045)	−0.024 (0.057)
as.factor(doy)12-23	0.036 (0.045)	0.051 (0.057)
as.factor(doy)12-24	−0.138*** (0.045)	−0.161*** (0.057)
as.factor(doy)12-25	−0.398*** (0.045)	−0.382*** (0.058)
as.factor(doy)12-26	0.165*** (0.045)	0.041 (0.057)
as.factor(doy)12-27	0.151*** (0.045)	0.027 (0.057)
as.factor(doy)12-28	0.147*** (0.045)	0.044 (0.058)
as.factor(doy)12-29	0.166*** (0.045)	0.150*** (0.058)
as.factor(doy)12-30	0.190*** (0.045)	0.104* (0.057)
as.factor(doy)12-31	−0.002 (0.045)	−0.067 (0.057)
Constant	3.544*** (0.081)	2.356*** (0.104)
Observations	21,011	20,781
R^2	0.728	0.918
Adjusted R^2	0.722	0.916
Residual Std. Error	1.982 (df = 20560)	2.552 (df = 20330)
F Statistic	122.544*** (df = 450; 20560)	507.212*** (df = 450; 20330)
Notes:	***Significant at the 1 percent level.<br>**Significant at the 5 percent level.<br>*Significant at the 10 percent level.	

Table 3: May 22 - Event Study

	log(triage45)	log(triage123)
	(1)	(2)
DkEventlower_bin	0.115 (0.079)	0.027 (0.100)
DkEvent-10	−0.081 (0.111)	−0.097 (0.140)
DkEvent-9	0.050 (0.111)	−0.121 (0.140)
DkEvent-8	−0.034 (0.111)	−0.133 (0.140)
DkEvent-7	0.139 (0.111)	0.045 (0.140)
DkEvent-6	0.031 (0.111)	−0.010 (0.140)

DkEvent-5	0.018 (0.111)	0.036 (0.140)
DkEvent-4	0.118 (0.111)	−0.097 (0.140)
DkEvent-3	0.100 (0.111)	−0.011 (0.140)
DkEvent-2	−0.071 (0.111)	−0.024 (0.140)
DkEvent-1	0.106 (0.111)	−0.103 (0.140)
DkEvent1	0.054 (0.111)	−0.163 (0.140)
DkEvent2	0.105 (0.111)	0.000 (0.140)
DkEvent3	0.056 (0.111)	−0.048 (0.140)
DkEvent4	0.013 (0.111)	0.078 (0.140)
DkEvent5	0.095 (0.111)	−0.142 (0.140)
DkEvent6	0.158 (0.111)	-0.301^{**} (0.140)
DkEvent7	0.118 (0.111)	−0.068 (0.140)
DkEvent8	0.051 (0.111)	−0.020 (0.140)
DkEvent9	0.044 (0.111)	−0.031 (0.140)
DkEvent10	0.003 (0.111)	−0.089 (0.140)
DkEvent11	0.119 (0.111)	−0.089 (0.140)
DkEvent12	0.124 (0.111)	0.008 (0.140)
DkEvent13	0.047 (0.111)	−0.047 (0.140)
DkEvent14	0.188^{*} (0.111)	0.057 (0.140)
DkEvent15	0.251^{**} (0.111)	−0.028 (0.140)
DkEvent16	0.055 (0.111)	0.062 (0.142)
DkEvent17	0.232^{**} (0.111)	0.045 (0.140)
DkEvent18	0.033 (0.111)	0.139 (0.140)
DkEvent19	0.080 (0.111)	−0.004 (0.140)
DkEvent20	−0.025 (0.111)	−0.048 (0.140)
DkEvent21	0.195^{*} (0.111)	0.099 (0.140)
DkEvent22	0.070 (0.111)	0.108 (0.140)
DkEvent23	0.171 (0.111)	0.060 (0.140)
DkEvent24	0.181 (0.111)	0.053 (0.140)
DkEvent25	0.141 (0.111)	−0.035 (0.140)
DkEvent26	0.197^{*} (0.111)	0.145 (0.140)
DkEvent27	0.185^{*} (0.111)	0.038 (0.140)
DkEvent28	0.166 (0.111)	0.093 (0.140)
DkEvent29	0.299^{***} (0.111)	−0.044 (0.140)
DkEvent30	0.205^{*} (0.111)	0.133 (0.140)
DkEvent31	0.163 (0.111)	0.100 (0.140)

DkEvent32	0.331*** (0.111)	−0.004 (0.140)
DkEvent33	0.192* (0.111)	0.064 (0.140)
DkEvent34	0.270** (0.111)	0.210 (0.140)
DkEvent35	0.280** (0.111)	0.158 (0.141)
DkEvent36	0.226** (0.111)	0.382*** (0.140)
DkEvent37	0.349*** (0.111)	0.198 (0.140)
DkEvent38	0.298*** (0.111)	0.273* (0.140)
DkEvent39	0.376*** (0.111)	0.224 (0.140)
DkEvent40	0.318*** (0.111)	0.151 (0.140)
DkEvent41	0.332*** (0.111)	0.120 (0.140)
DkEvent42	0.341*** (0.111)	0.162 (0.140)
DkEvent43	0.245** (0.111)	0.110 (0.140)
DkEvent44	0.414*** (0.111)	−0.045 (0.140)
DkEvent45	0.200* (0.111)	0.194 (0.140)
DkEvent46	0.189* (0.111)	0.142 (0.140)
DkEvent47	0.098 (0.111)	0.025 (0.140)
DkEvent48	0.171 (0.111)	0.133 (0.140)
DkEvent49	0.081 (0.111)	0.081 (0.140)
DkEvent50	0.351*** (0.111)	0.151 (0.140)
DkEventupper_bin	0.229*** (0.079)	0.123 (0.100)
lag(DailyTotals, n = 1)	−0.002*** (0.000)	−0.002*** (0.000)
TotalVaccinated	0.000*** (0.000)	0.000*** (0.000)
EDFacility1	0.646*** (0.009)	1.782*** (0.011)
EDFacility2	−1.128*** (0.015)	−1.604*** (0.020)
EDFacility3	−0.211*** (0.011)	−0.376*** (0.014)
EDFacility4	0.206*** (0.009)	1.305*** (0.012)
EDFacility5	0.452*** (0.009)	2.022*** (0.011)
EDFacility6	0.625*** (0.009)	0.378*** (0.012)
EDFacility7	−0.017 (0.011)	−0.841*** (0.014)
EDFacility8	0.523*** (0.009)	2.135*** (0.011)
EDFacility9	0.495*** (0.010)	0.430*** (0.012)
EDFacility10	0.187*** (0.010)	0.232*** (0.013)
EDFacility11	0.305*** (0.010)	0.682*** (0.012)
as.factor(yr)2018	−0.041*** (0.005)	0.019*** (0.007)
as.factor(yr)2019	−0.116*** (0.005)	0.020*** (0.007)
as.factor(yr)2020	−0.477*** (0.008)	−0.248*** (0.010)

as.factor(yr)2021	−0.583*** (0.013)	−0.243*** (0.017)
as.factor(dow)monday	0.088*** (0.007)	0.085*** (0.008)
as.factor(dow)saturday	−0.060*** (0.007)	−0.060*** (0.008)
as.factor(dow)sunday	−0.043*** (0.007)	−0.050*** (0.008)
as.factor(dow)thursday	0.002 (0.007)	0.007 (0.008)
as.factor(dow)tuesday	0.016** (0.007)	0.020** (0.008)
as.factor(dow)wednesday	0.003 (0.007)	0.018** (0.008)
as.factor(doy)01-02	0.197*** (0.045)	0.029 (0.056)
as.factor(doy)01-03	0.140*** (0.045)	0.097* (0.056)
as.factor(doy)01-04	0.043 (0.045)	−0.029 (0.056)
as.factor(doy)01-05	0.090** (0.045)	0.009 (0.056)
as.factor(doy)01-06	0.096** (0.045)	−0.031 (0.056)
as.factor(doy)01-07	0.023 (0.045)	−0.004 (0.056)
as.factor(doy)01-08	−0.004 (0.044)	−0.119** (0.056)
as.factor(doy)01-09	−0.015 (0.045)	−0.069 (0.056)
as.factor(doy)01-10	−0.000 (0.045)	−0.086 (0.056)
as.factor(doy)01-11	0.028 (0.045)	−0.069 (0.056)
as.factor(doy)01-12	−0.032 (0.045)	−0.067 (0.056)
as.factor(doy)01-13	0.017 (0.045)	−0.098* (0.056)
as.factor(doy)01-14	−0.021 (0.045)	−0.080 (0.056)
as.factor(doy)01-15	0.020 (0.045)	−0.052 (0.056)
as.factor(doy)01-16	−0.037 (0.045)	−0.025 (0.056)
as.factor(doy)01-17	−0.021 (0.045)	−0.115** (0.056)
as.factor(doy)01-18	0.018 (0.045)	−0.046 (0.056)
as.factor(doy)01-19	−0.014 (0.045)	−0.078 (0.056)
as.factor(doy)01-20	−0.096** (0.045)	−0.201*** (0.056)
as.factor(doy)01-21	−0.033 (0.045)	−0.043 (0.056)
as.factor(doy)01-22	0.041 (0.045)	−0.033 (0.056)
as.factor(doy)01-23	−0.020 (0.045)	−0.086 (0.056)
as.factor(doy)01-24	−0.084* (0.045)	−0.084 (0.056)
as.factor(doy)01-25	0.007 (0.045)	−0.017 (0.056)
as.factor(doy)01-26	0.069 (0.045)	−0.066 (0.056)
as.factor(doy)01-27	0.073 (0.045)	0.043 (0.056)
as.factor(doy)01-28	0.078* (0.045)	0.013 (0.056)
as.factor(doy)01-29	0.077* (0.045)	0.005 (0.056)
as.factor(doy)01-30	0.003 (0.045)	0.013 (0.056)

as.factor(doy)01-31	0.033 (0.045)	−0.033 (0.056)
as.factor(doy)02-01	0.087* (0.045)	−0.026 (0.056)
as.factor(doy)02-02	−0.068 (0.045)	−0.132** (0.056)
as.factor(doy)02-03	0.078* (0.045)	0.002 (0.056)
as.factor(doy)02-04	0.071 (0.045)	0.030 (0.056)
as.factor(doy)02-05	0.078* (0.045)	0.087 (0.056)
as.factor(doy)02-06	0.055 (0.045)	0.036 (0.056)
as.factor(doy)02-07	−0.015 (0.045)	−0.048 (0.056)
as.factor(doy)02-08	−0.008 (0.045)	−0.042 (0.056)
as.factor(doy)02-09	0.077* (0.045)	−0.016 (0.056)
as.factor(doy)02-10	−0.013 (0.045)	−0.029 (0.056)
as.factor(doy)02-11	−0.011 (0.045)	−0.020 (0.056)
as.factor(doy)02-12	0.125*** (0.044)	0.038 (0.056)
as.factor(doy)02-13	−0.238*** (0.045)	−0.278*** (0.056)
as.factor(doy)02-14	0.015 (0.045)	0.045 (0.056)
as.factor(doy)02-15	0.080* (0.045)	−0.001 (0.056)
as.factor(doy)02-16	−0.064 (0.045)	−0.069 (0.056)
as.factor(doy)02-17	0.076* (0.045)	0.041 (0.056)
as.factor(doy)02-18	0.126*** (0.045)	0.072 (0.056)
as.factor(doy)02-19	0.140*** (0.044)	0.067 (0.056)
as.factor(doy)02-20	0.132*** (0.045)	0.049 (0.056)
as.factor(doy)02-21	0.055 (0.045)	0.063 (0.056)
as.factor(doy)02-22	0.091** (0.045)	0.069 (0.056)
as.factor(doy)02-23	0.097** (0.045)	0.036 (0.056)
as.factor(doy)02-24	0.057 (0.045)	0.073 (0.056)
as.factor(doy)02-25	0.085* (0.045)	0.039 (0.056)
as.factor(doy)02-26	0.079* (0.044)	0.032 (0.056)
as.factor(doy)02-27	0.033 (0.045)	−0.050 (0.056)
as.factor(doy)02-28	0.035 (0.045)	0.076 (0.056)
as.factor(doy)02-29	0.307*** (0.077)	0.253*** (0.097)
as.factor(doy)03-01	0.074* (0.045)	−0.021 (0.056)
as.factor(doy)03-02	0.005 (0.045)	0.021 (0.056)
as.factor(doy)03-03	0.116*** (0.045)	0.028 (0.056)
as.factor(doy)03-04	0.020 (0.045)	−0.037 (0.056)
as.factor(doy)03-05	0.064 (0.045)	0.046 (0.056)
as.factor(doy)03-06	0.074* (0.045)	0.015 (0.056)

as.factor(doy)03-07	0.053 (0.045)	0.005 (0.056)
as.factor(doy)03-08	0.036 (0.045)	−0.086 (0.056)
as.factor(doy)03-09	0.093** (0.045)	0.059 (0.056)
as.factor(doy)03-10	0.043 (0.045)	0.053 (0.056)
as.factor(doy)03-11	0.040 (0.045)	0.027 (0.056)
as.factor(doy)03-12	0.107** (0.045)	0.065 (0.056)
as.factor(doy)03-13	0.092** (0.045)	−0.042 (0.056)
as.factor(doy)03-14	−0.049 (0.045)	−0.077 (0.056)
as.factor(doy)03-15	−0.001 (0.045)	−0.033 (0.056)
as.factor(doy)03-16	0.013 (0.045)	−0.027 (0.056)
as.factor(doy)03-17	−0.048 (0.045)	−0.027 (0.056)
as.factor(doy)03-18	−0.013 (0.045)	−0.058 (0.056)
as.factor(doy)03-19	−0.034 (0.045)	−0.073 (0.056)
as.factor(doy)03-20	−0.096** (0.045)	−0.136** (0.056)
as.factor(doy)03-21	−0.030 (0.045)	−0.138** (0.056)
as.factor(doy)03-22	−0.131*** (0.045)	−0.177*** (0.056)
as.factor(doy)03-23	−0.053 (0.045)	−0.159*** (0.056)
as.factor(doy)03-24	−0.047 (0.045)	−0.134** (0.056)
as.factor(doy)03-25	−0.062 (0.045)	−0.081 (0.056)
as.factor(doy)03-26	−0.043 (0.045)	−0.121** (0.056)
as.factor(doy)03-27	−0.119*** (0.045)	−0.184*** (0.056)
as.factor(doy)03-28	−0.113** (0.045)	−0.141** (0.056)
as.factor(doy)03-29	−0.081* (0.045)	−0.052 (0.056)
as.factor(doy)03-30	−0.077* (0.045)	−0.177*** (0.056)
as.factor(doy)03-31	−0.073 (0.045)	−0.134** (0.056)
as.factor(doy)04-01	−0.082* (0.045)	−0.176*** (0.056)
as.factor(doy)04-02	−0.125*** (0.045)	−0.126** (0.056)
as.factor(doy)04-03	−0.080* (0.045)	−0.198*** (0.056)
as.factor(doy)04-04	−0.143*** (0.045)	−0.188*** (0.056)
as.factor(doy)04-05	−0.134*** (0.045)	−0.073 (0.056)
as.factor(doy)04-06	−0.127*** (0.045)	−0.133** (0.056)
as.factor(doy)04-07	−0.053 (0.045)	−0.140** (0.056)
as.factor(doy)04-08	−0.203*** (0.045)	−0.131** (0.056)
as.factor(doy)04-09	−0.112** (0.045)	−0.160*** (0.056)
as.factor(doy)04-10	−0.139*** (0.045)	−0.116** (0.056)
as.factor(doy)04-11	−0.058 (0.045)	−0.131** (0.056)

as.factor(doy)04-12	-0.109^{**} (0.045)	-0.178^{***} (0.056)
as.factor(doy)04-13	-0.107^{**} (0.045)	-0.112^{**} (0.056)
as.factor(doy)04-14	-0.069 (0.045)	-0.146^{***} (0.056)
as.factor(doy)04-15	-0.075^{*} (0.045)	-0.092^{*} (0.056)
as.factor(doy)04-16	-0.139^{***} (0.045)	-0.145^{***} (0.056)
as.factor(doy)04-17	-0.082^{*} (0.045)	-0.139^{**} (0.056)
as.factor(doy)04-18	-0.068 (0.045)	-0.155^{***} (0.056)
as.factor(doy)04-19	-0.080^{*} (0.045)	-0.105^{*} (0.056)
as.factor(doy)04-20	-0.111^{**} (0.045)	-0.110^{**} (0.056)
as.factor(doy)04-21	-0.035 (0.045)	-0.116^{**} (0.056)
as.factor(doy)04-22	-0.065 (0.045)	-0.086 (0.056)
as.factor(doy)04-23	-0.036 (0.045)	-0.086 (0.056)
as.factor(doy)04-24	-0.057 (0.045)	-0.125^{**} (0.056)
as.factor(doy)04-25	-0.090^{**} (0.045)	-0.106^{*} (0.056)
as.factor(doy)04-26	-0.066 (0.045)	-0.113^{**} (0.056)
as.factor(doy)04-27	-0.035 (0.045)	-0.099^{*} (0.056)
as.factor(doy)04-28	-0.099^{**} (0.045)	-0.135^{**} (0.056)
as.factor(doy)04-29	0.009 (0.045)	-0.083 (0.056)
as.factor(doy)04-30	-0.010 (0.045)	-0.130^{**} (0.056)
as.factor(doy)05-01	-0.017 (0.045)	-0.104^{*} (0.056)
as.factor(doy)05-02	-0.022 (0.045)	-0.104^{*} (0.056)
as.factor(doy)05-03	0.031 (0.045)	0.007 (0.056)
as.factor(doy)05-04	-0.013 (0.045)	-0.060 (0.056)
as.factor(doy)05-05	-0.000 (0.045)	-0.052 (0.056)
as.factor(doy)05-06	-0.037 (0.045)	-0.027 (0.056)
as.factor(doy)05-07	0.006 (0.045)	-0.058 (0.056)
as.factor(doy)05-08	-0.041 (0.045)	-0.089 (0.056)
as.factor(doy)05-09	-0.127^{***} (0.045)	-0.123^{**} (0.056)
as.factor(doy)05-10	-0.045 (0.045)	-0.044 (0.056)
as.factor(doy)05-11	-0.043 (0.045)	-0.058 (0.056)
as.factor(doy)05-12	0.018 (0.047)	-0.104^{*} (0.059)
as.factor(doy)05-13	-0.059 (0.047)	-0.010 (0.059)
as.factor(doy)05-14	-0.005 (0.047)	-0.096 (0.059)
as.factor(doy)05-15	-0.047 (0.047)	-0.062 (0.059)
as.factor(doy)05-16	0.029 (0.047)	-0.081 (0.059)
as.factor(doy)05-17	-0.014 (0.047)	-0.073 (0.059)

as.factor(doy)05-18	−0.032 (0.047)	−0.031 (0.059)
as.factor(doy)05-19	0.063 (0.047)	−0.062 (0.059)
as.factor(doy)05-20	0.085* (0.047)	−0.044 (0.059)
as.factor(doy)05-21	0.021 (0.047)	−0.017 (0.059)
as.factor(doy)05-22	0.019 (0.047)	−0.105* (0.059)
as.factor(doy)05-23	0.049 (0.047)	0.011 (0.059)
as.factor(doy)05-24	−0.030 (0.047)	−0.037 (0.059)
as.factor(doy)05-25	0.050 (0.047)	−0.022 (0.059)
as.factor(doy)05-26	0.058 (0.047)	−0.078 (0.059)
as.factor(doy)05-27	−0.003 (0.047)	0.014 (0.059)
as.factor(doy)05-28	0.028 (0.047)	0.014 (0.059)
as.factor(doy)05-29	−0.007 (0.047)	−0.035 (0.059)
as.factor(doy)05-30	−0.012 (0.047)	−0.052 (0.059)
as.factor(doy)05-31	−0.031 (0.047)	−0.062 (0.059)
as.factor(doy)06-01	−0.008 (0.047)	−0.041 (0.059)
as.factor(doy)06-02	−0.002 (0.047)	−0.076 (0.059)
as.factor(doy)06-03	0.019 (0.047)	−0.010 (0.059)
as.factor(doy)06-04	0.062 (0.047)	−0.062 (0.059)
as.factor(doy)06-05	−0.007 (0.047)	−0.061 (0.059)
as.factor(doy)06-06	0.017 (0.047)	−0.034 (0.059)
as.factor(doy)06-07	−0.016 (0.047)	−0.057 (0.059)
as.factor(doy)06-08	−0.048 (0.047)	−0.047 (0.059)
as.factor(doy)06-09	0.018 (0.047)	−0.093 (0.059)
as.factor(doy)06-10	0.036 (0.047)	−0.013 (0.059)
as.factor(doy)06-11	0.041 (0.047)	−0.099* (0.059)
as.factor(doy)06-12	−0.023 (0.047)	−0.092 (0.059)
as.factor(doy)06-13	0.025 (0.047)	−0.086 (0.059)
as.factor(doy)06-14	−0.014 (0.047)	−0.071 (0.059)
as.factor(doy)06-15	0.029 (0.047)	−0.079 (0.059)
as.factor(doy)06-16	−0.005 (0.048)	−0.102* (0.059)
as.factor(doy)06-17	−0.031 (0.047)	−0.151** (0.059)
as.factor(doy)06-18	0.005 (0.047)	−0.060 (0.059)
as.factor(doy)06-19	0.045 (0.047)	−0.125** (0.059)
as.factor(doy)06-20	−0.046 (0.047)	−0.103* (0.059)
as.factor(doy)06-21	−0.088* (0.047)	−0.086 (0.059)
as.factor(doy)06-22	0.006 (0.047)	−0.107* (0.059)

as.factor(doy)06-23	0.004 (0.047)	-0.076 (0.059)
as.factor(doy)06-24	0.003 (0.048)	-0.117^{*} (0.060)
as.factor(doy)06-25	-0.011 (0.047)	-0.090 (0.060)
as.factor(doy)06-26	0.024 (0.047)	-0.113^{*} (0.059)
as.factor(doy)06-27	-0.022 (0.047)	-0.157^{***} (0.059)
as.factor(doy)06-28	0.009 (0.047)	-0.148^{**} (0.059)
as.factor(doy)06-29	-0.035 (0.047)	-0.140^{**} (0.059)
as.factor(doy)06-30	0.021 (0.048)	-0.105^{*} (0.059)
as.factor(doy)07-01	-0.036 (0.047)	-0.204^{***} (0.059)
as.factor(doy)07-02	0.059 (0.047)	-0.044 (0.059)
as.factor(doy)07-03	0.042 (0.048)	-0.053 (0.060)
as.factor(doy)07-04	0.014 (0.048)	-0.136^{**} (0.060)
as.factor(doy)07-05	-0.033 (0.047)	-0.024 (0.060)
as.factor(doy)07-06	-0.011 (0.047)	-0.120^{**} (0.059)
as.factor(doy)07-07	0.053 (0.047)	-0.101^{*} (0.059)
as.factor(doy)07-08	0.041 (0.047)	-0.032 (0.059)
as.factor(doy)07-09	-0.011 (0.047)	-0.038 (0.059)
as.factor(doy)07-10	0.062 (0.047)	-0.031 (0.059)
as.factor(doy)07-11	-0.059 (0.047)	-0.103^{*} (0.059)
as.factor(doy)07-12	0.037 (0.045)	-0.061 (0.056)
as.factor(doy)07-13	0.052 (0.045)	-0.092^{*} (0.056)
as.factor(doy)07-14	0.056 (0.045)	-0.091 (0.056)
as.factor(doy)07-15	0.048 (0.045)	-0.072 (0.056)
as.factor(doy)07-16	-0.024 (0.045)	-0.039 (0.056)
as.factor(doy)07-17	-0.031 (0.045)	-0.092^{*} (0.056)
as.factor(doy)07-18	-0.010 (0.045)	-0.121^{**} (0.056)
as.factor(doy)07-19	-0.020 (0.045)	-0.059 (0.056)
as.factor(doy)07-20	0.037 (0.045)	-0.081 (0.056)
as.factor(doy)07-21	0.035 (0.045)	-0.115^{**} (0.056)
as.factor(doy)07-22	0.078^{*} (0.045)	-0.078 (0.056)
as.factor(doy)07-23	0.094^{**} (0.045)	-0.102^{*} (0.056)
as.factor(doy)07-24	0.043 (0.045)	-0.095^{*} (0.056)
as.factor(doy)07-25	0.037 (0.045)	-0.067 (0.056)
as.factor(doy)07-26	0.053 (0.045)	-0.064 (0.056)
as.factor(doy)07-27	0.039 (0.045)	-0.086 (0.056)
as.factor(doy)07-28	0.070 (0.045)	-0.042 (0.056)

as.factor(doy)07-29	0.104** (0.045)	−0.085 (0.056)
as.factor(doy)07-30	0.046 (0.045)	−0.111** (0.056)
as.factor(doy)07-31	0.052 (0.045)	−0.087 (0.056)
as.factor(doy)08-01	0.024 (0.045)	−0.168*** (0.056)
as.factor(doy)08-02	0.025 (0.045)	−0.083 (0.056)
as.factor(doy)08-03	0.107** (0.045)	−0.120** (0.056)
as.factor(doy)08-04	0.041 (0.045)	−0.008 (0.056)
as.factor(doy)08-05	0.059 (0.045)	−0.090 (0.056)
as.factor(doy)08-06	0.094** (0.045)	−0.075 (0.056)
as.factor(doy)08-07	0.069 (0.045)	−0.087 (0.056)
as.factor(doy)08-08	0.088** (0.045)	−0.067 (0.056)
as.factor(doy)08-09	0.036 (0.045)	−0.066 (0.056)
as.factor(doy)08-10	0.015 (0.045)	−0.093* (0.056)
as.factor(doy)08-11	0.018 (0.045)	−0.054 (0.056)
as.factor(doy)08-12	0.055 (0.045)	−0.063 (0.056)
as.factor(doy)08-13	0.056 (0.045)	−0.106* (0.056)
as.factor(doy)08-14	0.005 (0.045)	−0.107* (0.056)
as.factor(doy)08-15	0.034 (0.045)	−0.172*** (0.056)
as.factor(doy)08-16	0.027 (0.045)	−0.061 (0.056)
as.factor(doy)08-17	0.010 (0.045)	−0.095* (0.056)
as.factor(doy)08-18	−0.002 (0.045)	−0.061 (0.056)
as.factor(doy)08-19	0.031 (0.045)	−0.081 (0.056)
as.factor(doy)08-20	0.001 (0.045)	−0.099* (0.056)
as.factor(doy)08-21	−0.022 (0.045)	−0.064 (0.056)
as.factor(doy)08-22	0.010 (0.045)	−0.124** (0.056)
as.factor(doy)08-23	0.041 (0.045)	−0.040 (0.056)
as.factor(doy)08-24	0.043 (0.045)	−0.093* (0.056)
as.factor(doy)08-25	−0.020 (0.045)	−0.060 (0.056)
as.factor(doy)08-26	−0.017 (0.045)	−0.073 (0.056)
as.factor(doy)08-27	0.003 (0.045)	−0.063 (0.056)
as.factor(doy)08-28	−0.060 (0.045)	−0.125** (0.056)
as.factor(doy)08-29	−0.045 (0.045)	−0.158*** (0.056)
as.factor(doy)08-30	−0.038 (0.045)	−0.048 (0.056)
as.factor(doy)08-31	−0.019 (0.045)	−0.090 (0.056)
as.factor(doy)09-01	−0.020 (0.045)	−0.122** (0.056)
as.factor(doy)09-02	−0.033 (0.045)	−0.156*** (0.056)

as.factor(doy)09-03	−0.043 (0.045)	−0.099* (0.056)
as.factor(doy)09-04	−0.013 (0.045)	−0.098* (0.056)
as.factor(doy)09-05	−0.039 (0.045)	−0.104* (0.056)
as.factor(doy)09-06	−0.022 (0.045)	−0.095* (0.056)
as.factor(doy)09-07	−0.061 (0.045)	−0.095* (0.056)
as.factor(doy)09-08	0.036 (0.045)	−0.109* (0.056)
as.factor(doy)09-09	−0.035 (0.045)	−0.055 (0.056)
as.factor(doy)09-10	−0.049 (0.045)	−0.074 (0.056)
as.factor(doy)09-11	−0.056 (0.045)	−0.077 (0.056)
as.factor(doy)09-12	−0.022 (0.045)	−0.073 (0.056)
as.factor(doy)09-13	0.015 (0.045)	0.046 (0.056)
as.factor(doy)09-14	−0.015 (0.045)	−0.085 (0.056)
as.factor(doy)09-15	−0.002 (0.045)	−0.032 (0.056)
as.factor(doy)09-16	0.029 (0.045)	−0.040 (0.056)
as.factor(doy)09-17	0.034 (0.045)	−0.051 (0.056)
as.factor(doy)09-18	−0.018 (0.045)	−0.018 (0.056)
as.factor(doy)09-19	−0.011 (0.045)	−0.103* (0.056)
as.factor(doy)09-20	0.025 (0.045)	−0.058 (0.056)
as.factor(doy)09-21	0.001 (0.045)	−0.044 (0.056)
as.factor(doy)09-22	0.056 (0.045)	−0.100* (0.056)
as.factor(doy)09-23	−0.009 (0.045)	−0.017 (0.056)
as.factor(doy)09-24	−0.013 (0.045)	0.000 (0.056)
as.factor(doy)09-25	−0.036 (0.045)	−0.043 (0.056)
as.factor(doy)09-26	0.025 (0.045)	−0.042 (0.056)
as.factor(doy)09-27	0.002 (0.045)	−0.090 (0.056)
as.factor(doy)09-28	0.012 (0.045)	−0.046 (0.056)
as.factor(doy)09-29	0.023 (0.045)	−0.078 (0.056)
as.factor(doy)09-30	−0.009 (0.045)	−0.076 (0.056)
as.factor(doy)10-01	−0.034 (0.045)	−0.043 (0.056)
as.factor(doy)10-02	−0.014 (0.045)	−0.046 (0.056)
as.factor(doy)10-03	−0.053 (0.045)	−0.028 (0.056)
as.factor(doy)10-04	−0.038 (0.045)	−0.047 (0.056)
as.factor(doy)10-05	0.004 (0.045)	−0.102* (0.056)
as.factor(doy)10-06	−0.025 (0.045)	−0.039 (0.056)
as.factor(doy)10-07	−0.003 (0.045)	−0.042 (0.056)
as.factor(doy)10-08	−0.028 (0.045)	−0.074 (0.056)

as.factor(doy)10-09	−0.069 (0.045)	−0.065 (0.056)
as.factor(doy)10-10	−0.025 (0.045)	−0.037 (0.056)
as.factor(doy)10-11	−0.056 (0.045)	−0.134** (0.056)
as.factor(doy)10-12	−0.064 (0.045)	−0.089 (0.056)
as.factor(doy)10-13	−0.024 (0.045)	−0.066 (0.056)
as.factor(doy)10-14	0.028 (0.045)	−0.136** (0.056)
as.factor(doy)10-15	0.023 (0.045)	−0.043 (0.056)
as.factor(doy)10-16	−0.023 (0.045)	−0.127** (0.056)
as.factor(doy)10-17	−0.146*** (0.045)	−0.084 (0.056)
as.factor(doy)10-18	−0.059 (0.045)	−0.085 (0.056)
as.factor(doy)10-19	−0.079* (0.045)	−0.096* (0.056)
as.factor(doy)10-20	−0.088** (0.045)	−0.111** (0.056)
as.factor(doy)10-21	−0.053 (0.047)	−0.058 (0.059)
as.factor(doy)10-22	−0.008 (0.047)	−0.039 (0.059)
as.factor(doy)10-23	−0.049 (0.047)	−0.082 (0.059)
as.factor(doy)10-24	−0.023 (0.048)	−0.109* (0.060)
as.factor(doy)10-25	−0.098** (0.047)	−0.102* (0.060)
as.factor(doy)10-26	−0.062 (0.047)	−0.078 (0.059)
as.factor(doy)10-27	−0.085* (0.047)	−0.086 (0.059)
as.factor(doy)10-28	−0.109** (0.047)	−0.106* (0.059)
as.factor(doy)10-29	−0.021 (0.047)	−0.063 (0.059)
as.factor(doy)10-30	−0.068 (0.047)	−0.118** (0.059)
as.factor(doy)10-31	−0.153*** (0.047)	−0.182*** (0.059)
as.factor(doy)11-01	−0.073 (0.047)	−0.044 (0.059)
as.factor(doy)11-02	−0.071 (0.047)	−0.078 (0.059)
as.factor(doy)11-03	−0.082* (0.047)	−0.088 (0.059)
as.factor(doy)11-04	−0.082* (0.047)	−0.098* (0.059)
as.factor(doy)11-05	−0.080* (0.047)	−0.089 (0.059)
as.factor(doy)11-06	−0.012 (0.047)	0.023 (0.059)
as.factor(doy)11-07	−0.146*** (0.047)	−0.108* (0.059)
as.factor(doy)11-08	−0.141*** (0.047)	−0.117** (0.059)
as.factor(doy)11-09	−0.040 (0.047)	−0.146** (0.059)
as.factor(doy)11-10	−0.021 (0.047)	−0.121** (0.059)
as.factor(doy)11-11	−0.083* (0.047)	−0.133** (0.059)
as.factor(doy)11-12	−0.088* (0.047)	−0.069 (0.059)
as.factor(doy)11-13	−0.085* (0.047)	−0.089 (0.059)

as.factor(doy)11-14	−0.111** (0.047)	−0.087 (0.059)
as.factor(doy)11-15	−0.098** (0.048)	−0.059 (0.060)
as.factor(doy)11-16	−0.138*** (0.047)	−0.206*** (0.060)
as.factor(doy)11-17	−0.084* (0.047)	−0.062 (0.059)
as.factor(doy)11-18	−0.091* (0.047)	−0.095 (0.059)
as.factor(doy)11-19	−0.075 (0.047)	−0.085 (0.059)
as.factor(doy)11-20	−0.093** (0.047)	−0.136** (0.059)
as.factor(doy)11-21	−0.155*** (0.047)	−0.131** (0.059)
as.factor(doy)11-22	−0.105** (0.047)	−0.125** (0.059)
as.factor(doy)11-23	−0.142*** (0.047)	−0.131** (0.059)
as.factor(doy)11-24	−0.101** (0.047)	−0.107* (0.059)
as.factor(doy)11-25	−0.102** (0.047)	−0.122** (0.059)
as.factor(doy)11-26	−0.066 (0.047)	−0.094 (0.059)
as.factor(doy)11-27	−0.039 (0.047)	−0.127** (0.059)
as.factor(doy)11-28	−0.154*** (0.047)	−0.148** (0.059)
as.factor(doy)11-29	−0.133*** (0.047)	−0.124** (0.059)
as.factor(doy)11-30	−0.146*** (0.047)	−0.164*** (0.059)
as.factor(doy)12-01	−0.098** (0.047)	−0.139** (0.059)
as.factor(doy)12-02	−0.113** (0.047)	−0.126** (0.059)
as.factor(doy)12-03	−0.207*** (0.047)	−0.158*** (0.059)
as.factor(doy)12-04	−0.109** (0.047)	−0.054 (0.059)
as.factor(doy)12-05	−0.140*** (0.047)	−0.134** (0.059)
as.factor(doy)12-06	−0.114** (0.047)	−0.135** (0.059)
as.factor(doy)12-07	−0.144*** (0.047)	−0.123** (0.059)
as.factor(doy)12-08	−0.193*** (0.047)	−0.124** (0.059)
as.factor(doy)12-09	−0.162*** (0.047)	−0.100* (0.059)
as.factor(doy)12-10	−0.123*** (0.047)	−0.089 (0.059)
as.factor(doy)12-11	−0.121** (0.047)	−0.016 (0.059)
as.factor(doy)12-12	−0.164*** (0.047)	−0.116** (0.059)
as.factor(doy)12-13	−0.222*** (0.047)	−0.147** (0.059)
as.factor(doy)12-14	−0.158*** (0.047)	−0.141** (0.059)
as.factor(doy)12-15	−0.141*** (0.047)	−0.133** (0.059)
as.factor(doy)12-16	−0.124*** (0.047)	−0.125** (0.059)
as.factor(doy)12-17	−0.106** (0.047)	−0.125** (0.059)
as.factor(doy)12-18	−0.129*** (0.048)	−0.192*** (0.059)
as.factor(doy)12-19	−0.137*** (0.047)	−0.215*** (0.059)

as.factor(doy)12-20	−0.121** (0.047)	−0.243*** (0.059)
as.factor(doy)12-21	−0.129*** (0.047)	−0.103* (0.060)
as.factor(doy)12-22	−0.043 (0.047)	−0.096 (0.059)
as.factor(doy)12-23	−0.034 (0.047)	−0.021 (0.059)
as.factor(doy)12-24	−0.207*** (0.047)	−0.232*** (0.059)
as.factor(doy)12-25	−0.468*** (0.047)	−0.453*** (0.059)
as.factor(doy)12-26	0.095** (0.047)	−0.031 (0.059)
as.factor(doy)12-27	0.080* (0.048)	−0.047 (0.059)
as.factor(doy)12-28	0.076 (0.048)	−0.028 (0.060)
as.factor(doy)12-29	0.096** (0.048)	0.078 (0.060)
as.factor(doy)12-30	0.120** (0.047)	0.032 (0.059)
as.factor(doy)12-31	−0.071 (0.047)	−0.138** (0.059)
Constant	3.506*** (0.085)	2.526*** (0.108)
Observations	21,011	20,781
R^2	0.693	0.913
Adjusted R^2	0.687	0.911
Residual Std. Error	2.105 (df = 20560)	2.639 (df = 20330)
F Statistic	103.373*** (df = 450; 20560)	471.489*** (df = 450; 20330)
Notes:	***Significant at the 1 percent level.	
	**Significant at the 5 percent level.	
	*Significant at the 10 percent level.	

Table 4: January 5 - Event Study

	log(triage45)	log(triage123)
	(1)	(2)
DkEventlower_bin	0.545*** (0.079)	0.246** (0.098)
DkEvent-10	0.423*** (0.113)	0.228 (0.142)
DkEvent-9	0.481*** (0.113)	0.336** (0.142)
DkEvent-8	0.510*** (0.113)	0.222 (0.142)
DkEvent-7	0.491*** (0.113)	0.173 (0.141)
DkEvent-6	0.423*** (0.113)	0.244* (0.142)
DkEvent-5	0.506*** (0.113)	0.161 (0.142)
DkEvent-4	0.138 (0.111)	−0.012 (0.139)
DkEvent-3	−0.142 (0.111)	−0.235* (0.139)

DkEvent-2	−0.043 (0.111)	−0.002 (0.139)
DkEvent-1	0.189* (0.111)	0.038 (0.139)
DkEvent1	−0.014 (0.111)	−0.083 (0.139)
DkEvent2	0.033 (0.111)	−0.024 (0.139)
DkEvent3	0.159 (0.111)	0.211 (0.139)
DkEvent4	0.190* (0.111)	0.287** (0.139)
DkEvent5	−0.033 (0.111)	0.143 (0.139)
DkEvent6	−0.096 (0.111)	0.033 (0.139)
DkEvent7	0.205* (0.111)	0.057 (0.139)
DkEvent8	0.228** (0.111)	0.072 (0.139)
DkEvent9	0.135 (0.111)	−0.043 (0.139)
DkEvent10	0.063 (0.111)	−0.011 (0.139)
DkEvent11	−0.142 (0.111)	−0.108 (0.139)
DkEvent12	0.048 (0.111)	0.070 (0.139)
DkEvent13	−0.017 (0.111)	0.044 (0.139)
DkEvent14	0.017 (0.111)	0.024 (0.139)
DkEvent15	−0.090 (0.111)	0.066 (0.139)
DkEvent16	0.017 (0.111)	0.004 (0.139)
DkEvent17	−0.008 (0.111)	−0.062 (0.139)
DkEvent18	0.052 (0.111)	0.018 (0.139)
DkEvent19	0.012 (0.111)	−0.104 (0.139)
DkEvent20	0.050 (0.111)	0.026 (0.139)
DkEvent21	0.006 (0.111)	−0.168 (0.139)
DkEvent22	−0.047 (0.111)	−0.123 (0.139)
DkEvent23	0.001 (0.111)	−0.108 (0.139)
DkEvent24	−0.135 (0.111)	0.006 (0.139)
DkEvent25	−0.025 (0.111)	−0.052 (0.139)
DkEvent26	0.028 (0.111)	−0.105 (0.139)
DkEvent27	−0.046 (0.111)	−0.132 (0.139)
DkEvent28	−0.451*** (0.111)	−0.559*** (0.139)
DkEvent29	−0.098 (0.111)	−0.015 (0.139)
DkEvent30	−0.024 (0.111)	0.066 (0.139)
DkEvent31	−0.060 (0.111)	−0.018 (0.140)
DkEvent32	−0.017 (0.111)	−0.027 (0.139)
DkEvent33	−0.076 (0.111)	0.049 (0.140)
DkEvent34	−0.008 (0.111)	−0.221 (0.139)

DkEvent35	0.142 (0.111)	−0.136 (0.139)
DkEvent36	0.135 (0.111)	−0.157 (0.139)
DkEvent37	−0.281** (0.111)	−0.028 (0.139)
DkEvent38	−0.166 (0.111)	−0.105 (0.139)
DkEvent39	0.377*** (0.111)	0.477*** (0.139)
DkEvent40	−0.048 (0.111)	0.001 (0.139)
DkEvent41	−0.108 (0.111)	−0.119 (0.139)
DkEvent42	−0.380*** (0.111)	−0.371*** (0.139)
DkEvent43	−0.117 (0.111)	−0.027 (0.139)
DkEvent44	0.032 (0.111)	−0.048 (0.139)
DkEvent45	0.059 (0.111)	0.088 (0.139)
DkEvent46	−0.151 (0.111)	0.074 (0.139)
DkEvent47	−0.084 (0.111)	−0.051 (0.139)
DkEvent48	−0.012 (0.111)	0.014 (0.139)
DkEvent49	−0.086 (0.111)	−0.066 (0.139)
DkEvent50	−0.045 (0.111)	0.012 (0.139)
DkEventupper_bin	0.173** (0.079)	0.218** (0.099)
lag(DailyTotals, n = 1)	−0.002*** (0.000)	−0.001*** (0.000)
TotalVaccinated	0.000** (0.000)	−0.000 (0.000)
EDFacility1	0.646*** (0.009)	1.782*** (0.011)
EDFacility2	−1.128*** (0.015)	−1.604*** (0.020)
EDFacility3	−0.211*** (0.011)	−0.376*** (0.014)
EDFacility4	0.205*** (0.009)	1.305*** (0.012)
EDFacility5	0.452*** (0.009)	2.022*** (0.011)
EDFacility6	0.625*** (0.009)	0.378*** (0.012)
EDFacility7	−0.017 (0.011)	−0.841*** (0.014)
EDFacility8	0.522*** (0.009)	2.135*** (0.011)
EDFacility9	0.495*** (0.010)	0.430*** (0.012)
EDFacility10	0.188*** (0.010)	0.232*** (0.013)
EDFacility11	0.305*** (0.010)	0.682*** (0.012)
as.factor(yr)2018	−0.041*** (0.005)	0.019*** (0.007)
as.factor(yr)2019	−0.116*** (0.005)	0.020*** (0.007)
as.factor(yr)2020	−0.417*** (0.005)	−0.202*** (0.007)
as.factor(yr)2021		
as.factor(dow)monday	0.089*** (0.007)	0.088*** (0.008)
as.factor(dow)saturday	−0.059*** (0.007)	−0.061*** (0.008)

as.factor(dow)sunday	−0.040*** (0.007)	−0.049*** (0.008)
as.factor(dow)thursday	0.001 (0.007)	0.006 (0.008)
as.factor(dow)tuesday	0.019*** (0.007)	0.026*** (0.008)
as.factor(dow)wednesday	0.004 (0.007)	0.019** (0.008)
as.factor(doy)01-02	0.253*** (0.050)	0.073 (0.062)
as.factor(doy)01-03	0.176*** (0.050)	0.095 (0.062)
as.factor(doy)01-04	0.033 (0.050)	−0.039 (0.062)
as.factor(doy)01-05	0.115** (0.050)	0.005 (0.062)
as.factor(doy)01-06	0.125** (0.050)	−0.019 (0.062)
as.factor(doy)01-07	0.042 (0.050)	−0.003 (0.062)
as.factor(doy)01-08	−0.009 (0.050)	−0.166*** (0.062)
as.factor(doy)01-09	−0.027 (0.050)	−0.131** (0.062)
as.factor(doy)01-10	0.033 (0.050)	−0.118* (0.062)
as.factor(doy)01-11	0.075 (0.050)	−0.078 (0.062)
as.factor(doy)01-12	−0.046 (0.050)	−0.081 (0.062)
as.factor(doy)01-13	−0.001 (0.050)	−0.116* (0.062)
as.factor(doy)01-14	−0.023 (0.050)	−0.076 (0.062)
as.factor(doy)01-15	0.033 (0.050)	−0.055 (0.062)
as.factor(doy)01-16	0.017 (0.050)	−0.008 (0.062)
as.factor(doy)01-17	−0.006 (0.050)	−0.135** (0.062)
as.factor(doy)01-18	0.048 (0.050)	−0.058 (0.062)
as.factor(doy)01-19	0.009 (0.050)	−0.088 (0.062)
as.factor(doy)01-20	−0.051 (0.050)	−0.217*** (0.062)
as.factor(doy)01-21	−0.012 (0.050)	−0.049 (0.062)
as.factor(doy)01-22	0.067 (0.050)	−0.027 (0.062)
as.factor(doy)01-23	−0.003 (0.050)	−0.093 (0.062)
as.factor(doy)01-24	−0.059 (0.050)	−0.067 (0.062)
as.factor(doy)01-25	0.024 (0.050)	−0.025 (0.062)
as.factor(doy)01-26	0.096* (0.050)	−0.034 (0.063)
as.factor(doy)01-27	0.110** (0.050)	0.066 (0.062)
as.factor(doy)01-28	0.103** (0.050)	0.030 (0.062)
as.factor(doy)01-29	0.131*** (0.050)	0.000 (0.062)
as.factor(doy)01-30	0.036 (0.050)	0.020 (0.062)
as.factor(doy)01-31	0.054 (0.050)	−0.017 (0.062)
as.factor(doy)02-01	0.124** (0.050)	0.000 (0.062)
as.factor(doy)02-02	0.049 (0.050)	−0.023 (0.062)

as.factor(doy)02-03	0.124** (0.050)	0.004 (0.062)
as.factor(doy)02-04	0.103** (0.050)	0.014 (0.062)
as.factor(doy)02-05	0.118** (0.050)	0.088 (0.062)
as.factor(doy)02-06	0.087* (0.050)	0.039 (0.062)
as.factor(doy)02-07	0.029 (0.050)	−0.060 (0.062)
as.factor(doy)02-08	0.023 (0.050)	0.003 (0.062)
as.factor(doy)02-09	0.076 (0.050)	0.010 (0.062)
as.factor(doy)02-10	−0.012 (0.050)	0.000 (0.062)
as.factor(doy)02-11	0.073 (0.050)	−0.016 (0.062)
as.factor(doy)02-12	0.187*** (0.050)	0.057 (0.062)
as.factor(doy)02-13	−0.286*** (0.050)	−0.376*** (0.062)
as.factor(doy)02-14	0.053 (0.050)	0.044 (0.062)
as.factor(doy)02-15	0.131*** (0.050)	0.023 (0.062)
as.factor(doy)02-16	0.041 (0.050)	0.005 (0.062)
as.factor(doy)02-17	0.128** (0.050)	0.046 (0.062)
as.factor(doy)02-18	0.148*** (0.050)	0.080 (0.062)
as.factor(doy)02-19	0.157*** (0.050)	0.047 (0.062)
as.factor(doy)02-20	0.190*** (0.050)	0.033 (0.062)
as.factor(doy)02-21	0.101** (0.050)	0.072 (0.062)
as.factor(doy)02-22	0.123** (0.050)	0.067 (0.062)
as.factor(doy)02-23	0.143*** (0.050)	0.049 (0.062)
as.factor(doy)02-24	0.096* (0.050)	0.071 (0.062)
as.factor(doy)02-25	0.079* (0.047)	−0.005 (0.059)
as.factor(doy)02-26	0.073 (0.047)	−0.013 (0.059)
as.factor(doy)02-27	0.028 (0.047)	−0.095 (0.059)
as.factor(doy)02-28	0.029 (0.047)	0.032 (0.059)
as.factor(doy)02-29	0.272*** (0.079)	0.198** (0.098)
as.factor(doy)03-01	0.069 (0.047)	−0.064 (0.059)
as.factor(doy)03-02	−0.001 (0.047)	−0.023 (0.059)
as.factor(doy)03-03	0.110** (0.047)	−0.017 (0.059)
as.factor(doy)03-04	0.014 (0.047)	−0.081 (0.059)
as.factor(doy)03-05	0.058 (0.047)	0.000 (0.059)
as.factor(doy)03-06	0.068 (0.047)	−0.030 (0.059)
as.factor(doy)03-07	0.048 (0.047)	−0.039 (0.059)
as.factor(doy)03-08	0.031 (0.047)	−0.130** (0.059)
as.factor(doy)03-09	0.088* (0.047)	0.015 (0.059)

as.factor(doy)03-10	0.038 (0.047)	0.010 (0.059)
as.factor(doy)03-11	0.035 (0.047)	−0.016 (0.059)
as.factor(doy)03-12	0.101^{**} (0.047)	0.020 (0.059)
as.factor(doy)03-13	0.087^{*} (0.047)	−0.086 (0.059)
as.factor(doy)03-14	−0.055 (0.047)	-0.121^{**} (0.059)
as.factor(doy)03-15	−0.006 (0.047)	−0.076 (0.059)
as.factor(doy)03-16	0.008 (0.047)	−0.071 (0.059)
as.factor(doy)03-17	−0.053 (0.047)	−0.070 (0.059)
as.factor(doy)03-18	−0.018 (0.047)	-0.101^{*} (0.059)
as.factor(doy)03-19	−0.039 (0.047)	-0.118^{**} (0.059)
as.factor(doy)03-20	-0.101^{**} (0.047)	-0.180^{***} (0.059)
as.factor(doy)03-21	−0.035 (0.047)	-0.181^{***} (0.059)
as.factor(doy)03-22	-0.136^{***} (0.047)	-0.220^{***} (0.059)
as.factor(doy)03-23	−0.058 (0.047)	-0.203^{***} (0.059)
as.factor(doy)03-24	−0.053 (0.047)	-0.178^{***} (0.059)
as.factor(doy)03-25	−0.069 (0.047)	-0.128^{**} (0.059)
as.factor(doy)03-26	−0.050 (0.047)	-0.167^{***} (0.059)
as.factor(doy)03-27	-0.125^{***} (0.047)	-0.230^{***} (0.059)
as.factor(doy)03-28	-0.119^{**} (0.047)	-0.185^{***} (0.059)
as.factor(doy)03-29	-0.087^{*} (0.047)	−0.096 (0.059)
as.factor(doy)03-30	-0.082^{*} (0.047)	-0.221^{***} (0.059)
as.factor(doy)03-31	-0.078^{*} (0.047)	-0.178^{***} (0.059)
as.factor(doy)04-01	-0.088^{*} (0.047)	-0.220^{***} (0.059)
as.factor(doy)04-02	-0.130^{***} (0.047)	-0.171^{***} (0.059)
as.factor(doy)04-03	-0.085^{*} (0.047)	-0.241^{***} (0.059)
as.factor(doy)04-04	-0.148^{***} (0.047)	-0.231^{***} (0.059)
as.factor(doy)04-05	-0.138^{***} (0.047)	-0.115^{*} (0.059)
as.factor(doy)04-06	-0.130^{***} (0.047)	-0.174^{***} (0.059)
as.factor(doy)04-07	−0.058 (0.047)	-0.183^{***} (0.059)
as.factor(doy)04-08	-0.206^{***} (0.047)	-0.173^{***} (0.059)
as.factor(doy)04-09	-0.116^{**} (0.047)	-0.203^{***} (0.059)
as.factor(doy)04-10	-0.144^{***} (0.047)	-0.160^{***} (0.059)
as.factor(doy)04-11	−0.061 (0.047)	-0.173^{***} (0.059)
as.factor(doy)04-12	-0.112^{**} (0.047)	-0.219^{***} (0.059)
as.factor(doy)04-13	-0.109^{**} (0.047)	-0.152^{***} (0.059)
as.factor(doy)04-14	−0.073 (0.047)	-0.188^{***} (0.059)

as.factor(doy)04-15	-0.078 (0.047)	-0.132^{**} (0.059)
as.factor(doy)04-16	-0.141^{***} (0.047)	-0.186^{***} (0.059)
as.factor(doy)04-17	-0.084^{*} (0.047)	-0.180^{***} (0.059)
as.factor(doy)04-18	-0.071 (0.047)	-0.195^{***} (0.059)
as.factor(doy)04-19	-0.081^{*} (0.047)	-0.144^{**} (0.059)
as.factor(doy)04-20	-0.112^{**} (0.047)	-0.149^{**} (0.059)
as.factor(doy)04-21	-0.037 (0.047)	-0.155^{***} (0.059)
as.factor(doy)04-22	-0.067 (0.047)	-0.126^{**} (0.059)
as.factor(doy)04-23	-0.044 (0.047)	-0.134^{**} (0.059)
as.factor(doy)04-24	-0.058 (0.047)	-0.165^{***} (0.059)
as.factor(doy)04-25	-0.091^{*} (0.047)	-0.144^{**} (0.059)
as.factor(doy)04-26	-0.067 (0.047)	-0.151^{**} (0.059)
as.factor(doy)04-27	-0.037 (0.047)	-0.139^{**} (0.059)
as.factor(doy)04-28	-0.100^{**} (0.047)	-0.173^{***} (0.059)
as.factor(doy)04-29	0.008 (0.047)	-0.120^{**} (0.059)
as.factor(doy)04-30	-0.012 (0.047)	-0.170^{***} (0.059)
as.factor(doy)05-01	-0.018 (0.047)	-0.143^{**} (0.059)
as.factor(doy)05-02	-0.022 (0.047)	-0.141^{**} (0.059)
as.factor(doy)05-03	0.030 (0.047)	-0.031 (0.059)
as.factor(doy)05-04	-0.013 (0.047)	-0.097 (0.059)
as.factor(doy)05-05	-0.001 (0.047)	-0.090 (0.059)
as.factor(doy)05-06	-0.037 (0.047)	-0.063 (0.059)
as.factor(doy)05-07	0.007 (0.047)	-0.095 (0.059)
as.factor(doy)05-08	-0.040 (0.047)	-0.126^{**} (0.059)
as.factor(doy)05-09	-0.126^{***} (0.047)	-0.160^{***} (0.059)
as.factor(doy)05-10	-0.044 (0.047)	-0.080 (0.059)
as.factor(doy)05-11	-0.041 (0.047)	-0.094 (0.059)
as.factor(doy)05-12	-0.020 (0.047)	-0.164^{***} (0.059)
as.factor(doy)05-13	-0.072 (0.047)	-0.076 (0.059)
as.factor(doy)05-14	-0.033 (0.047)	-0.164^{***} (0.059)
as.factor(doy)05-15	-0.040 (0.047)	-0.094 (0.059)
as.factor(doy)05-16	0.014 (0.047)	-0.124^{**} (0.059)
as.factor(doy)05-17	-0.032 (0.047)	-0.107^{*} (0.059)
as.factor(doy)05-18	-0.029 (0.047)	-0.091 (0.059)
as.factor(doy)05-19	0.062 (0.047)	-0.105^{*} (0.059)
as.factor(doy)05-20	0.051 (0.047)	-0.088 (0.059)

as.factor(doy)05-21	0.021 (0.047)	−0.078 (0.059)
as.factor(doy)05-22	0.000 (0.047)	−0.143** (0.059)
as.factor(doy)05-23	0.039 (0.047)	−0.061 (0.059)
as.factor(doy)05-24	−0.029 (0.047)	−0.076 (0.059)
as.factor(doy)05-25	0.042 (0.047)	−0.070 (0.059)
as.factor(doy)05-26	0.041 (0.047)	−0.101* (0.059)
as.factor(doy)05-27	−0.002 (0.047)	−0.051 (0.059)
as.factor(doy)05-28	0.040 (0.047)	−0.085 (0.059)
as.factor(doy)05-29	−0.002 (0.047)	−0.087 (0.059)
as.factor(doy)05-30	−0.020 (0.047)	−0.093 (0.059)
as.factor(doy)05-31	−0.041 (0.047)	−0.105* (0.059)
as.factor(doy)06-01	−0.024 (0.047)	−0.095 (0.059)
as.factor(doy)06-02	0.004 (0.047)	−0.131** (0.059)
as.factor(doy)06-03	0.026 (0.047)	−0.045 (0.059)
as.factor(doy)06-04	0.054 (0.047)	−0.107* (0.059)
as.factor(doy)06-05	0.014 (0.047)	−0.085 (0.059)
as.factor(doy)06-06	0.051 (0.047)	−0.074 (0.059)
as.factor(doy)06-07	−0.021 (0.047)	−0.078 (0.059)
as.factor(doy)06-08	−0.018 (0.047)	−0.073 (0.059)
as.factor(doy)06-09	0.008 (0.047)	−0.100* (0.059)
as.factor(doy)06-10	0.037 (0.047)	−0.047 (0.059)
as.factor(doy)06-11	0.021 (0.047)	−0.142** (0.059)
as.factor(doy)06-12	0.000 (0.047)	−0.106* (0.059)
as.factor(doy)06-13	0.024 (0.047)	−0.097 (0.059)
as.factor(doy)06-14	0.006 (0.047)	−0.091 (0.059)
as.factor(doy)06-15	0.051 (0.047)	−0.101* (0.059)
as.factor(doy)06-16	0.008 (0.048)	−0.142** (0.059)
as.factor(doy)06-17	−0.005 (0.047)	−0.153*** (0.059)
as.factor(doy)06-18	0.029 (0.047)	−0.084 (0.059)
as.factor(doy)06-19	0.065 (0.047)	−0.138** (0.059)
as.factor(doy)06-20	0.001 (0.047)	−0.142** (0.059)
as.factor(doy)06-21	−0.059 (0.047)	−0.088 (0.059)
as.factor(doy)06-22	0.027 (0.047)	−0.117** (0.059)
as.factor(doy)06-23	0.058 (0.047)	−0.107* (0.059)
as.factor(doy)06-24	0.029 (0.047)	−0.132** (0.059)
as.factor(doy)06-25	0.032 (0.047)	−0.077 (0.059)

as.factor(doy)06-26	0.069 (0.047)	−0.111* (0.059)
as.factor(doy)06-27	0.013 (0.047)	−0.108* (0.059)
as.factor(doy)06-28	0.069 (0.047)	−0.135** (0.059)
as.factor(doy)06-29	0.014 (0.047)	−0.112* (0.059)
as.factor(doy)06-30	0.088* (0.048)	−0.087 (0.059)
as.factor(doy)07-01	0.018 (0.047)	−0.199*** (0.059)
as.factor(doy)07-02	0.116** (0.047)	−0.047 (0.059)
as.factor(doy)07-03	0.102** (0.047)	−0.046 (0.059)
as.factor(doy)07-04	0.055 (0.047)	−0.138** (0.059)
as.factor(doy)07-05	0.042 (0.047)	−0.058 (0.059)
as.factor(doy)07-06	0.022 (0.047)	−0.106* (0.059)
as.factor(doy)07-07	0.084* (0.047)	−0.097 (0.059)
as.factor(doy)07-08	0.053 (0.047)	−0.050 (0.059)
as.factor(doy)07-09	0.017 (0.047)	−0.035 (0.059)
as.factor(doy)07-10	0.072 (0.047)	−0.038 (0.059)
as.factor(doy)07-11	0.005 (0.047)	−0.095 (0.059)
as.factor(doy)07-12	0.077 (0.047)	−0.059 (0.059)
as.factor(doy)07-13	0.092* (0.047)	−0.090 (0.059)
as.factor(doy)07-14	0.096** (0.047)	−0.089 (0.059)
as.factor(doy)07-15	0.088* (0.047)	−0.069 (0.059)
as.factor(doy)07-16	0.017 (0.047)	−0.037 (0.059)
as.factor(doy)07-17	0.010 (0.047)	−0.089 (0.059)
as.factor(doy)07-18	0.031 (0.047)	−0.117** (0.059)
as.factor(doy)07-19	0.021 (0.047)	−0.055 (0.059)
as.factor(doy)07-20	0.078* (0.047)	−0.077 (0.059)
as.factor(doy)07-21	0.077 (0.047)	−0.111* (0.059)
as.factor(doy)07-22	0.120** (0.047)	−0.073 (0.059)
as.factor(doy)07-23	0.135*** (0.047)	−0.098* (0.059)
as.factor(doy)07-24	0.085* (0.047)	−0.090 (0.059)
as.factor(doy)07-25	0.079* (0.047)	−0.061 (0.059)
as.factor(doy)07-26	0.095** (0.047)	−0.058 (0.059)
as.factor(doy)07-27	0.082* (0.047)	−0.081 (0.059)
as.factor(doy)07-28	0.112** (0.047)	−0.037 (0.059)
as.factor(doy)07-29	0.146*** (0.047)	−0.079 (0.059)
as.factor(doy)07-30	0.088* (0.047)	−0.107* (0.059)
as.factor(doy)07-31	0.095** (0.047)	−0.081 (0.059)

as.factor(doy)08-01	0.066 (0.047)	−0.164*** (0.059)
as.factor(doy)08-02	0.068 (0.047)	−0.076 (0.059)
as.factor(doy)08-03	0.149*** (0.047)	−0.115* (0.059)
as.factor(doy)08-04	0.084* (0.047)	−0.002 (0.059)
as.factor(doy)08-05	0.101** (0.047)	−0.084 (0.059)
as.factor(doy)08-06	0.137*** (0.047)	−0.070 (0.059)
as.factor(doy)08-07	0.113** (0.047)	−0.081 (0.059)
as.factor(doy)08-08	0.130*** (0.047)	−0.063 (0.059)
as.factor(doy)08-09	0.080* (0.047)	−0.059 (0.059)
as.factor(doy)08-10	0.058 (0.047)	−0.086 (0.059)
as.factor(doy)08-11	0.060 (0.047)	−0.049 (0.059)
as.factor(doy)08-12	0.098** (0.047)	−0.056 (0.059)
as.factor(doy)08-13	0.098** (0.047)	−0.102* (0.059)
as.factor(doy)08-14	0.049 (0.047)	−0.101* (0.059)
as.factor(doy)08-15	0.077 (0.047)	−0.165*** (0.059)
as.factor(doy)08-16	0.067 (0.047)	−0.059 (0.059)
as.factor(doy)08-17	0.052 (0.047)	−0.090 (0.059)
as.factor(doy)08-18	0.040 (0.047)	−0.056 (0.059)
as.factor(doy)08-19	0.073 (0.047)	−0.076 (0.059)
as.factor(doy)08-20	0.042 (0.047)	−0.096 (0.059)
as.factor(doy)08-21	0.022 (0.047)	−0.057 (0.059)
as.factor(doy)08-22	0.055 (0.047)	−0.116** (0.059)
as.factor(doy)08-23	0.080* (0.047)	−0.039 (0.059)
as.factor(doy)08-24	0.086* (0.047)	−0.088 (0.059)
as.factor(doy)08-25	0.024 (0.047)	−0.053 (0.059)
as.factor(doy)08-26	0.027 (0.047)	−0.065 (0.059)
as.factor(doy)08-27	0.047 (0.047)	−0.057 (0.059)
as.factor(doy)08-28	−0.015 (0.047)	−0.118** (0.059)
as.factor(doy)08-29	−0.001 (0.047)	−0.149** (0.059)
as.factor(doy)08-30	0.003 (0.047)	−0.044 (0.059)
as.factor(doy)08-31	0.026 (0.047)	−0.082 (0.059)
as.factor(doy)09-01	0.024 (0.047)	−0.115* (0.059)
as.factor(doy)09-02	0.011 (0.047)	−0.148** (0.059)
as.factor(doy)09-03	0.001 (0.047)	−0.093 (0.059)
as.factor(doy)09-04	0.032 (0.047)	−0.090 (0.059)
as.factor(doy)09-05	0.007 (0.047)	−0.095 (0.059)

as.factor(doy)09-06	0.024 (0.047)	−0.086 (0.059)
as.factor(doy)09-07	−0.021 (0.047)	−0.093 (0.059)
as.factor(doy)09-08	0.079* (0.048)	−0.103* (0.059)
as.factor(doy)09-09	0.009 (0.047)	−0.046 (0.059)
as.factor(doy)09-10	−0.006 (0.047)	−0.068 (0.059)
as.factor(doy)09-11	−0.011 (0.047)	−0.068 (0.059)
as.factor(doy)09-12	0.023 (0.047)	−0.064 (0.059)
as.factor(doy)09-13	0.049 (0.047)	0.040 (0.059)
as.factor(doy)09-14	0.026 (0.047)	−0.082 (0.059)
as.factor(doy)09-15	0.037 (0.047)	−0.030 (0.059)
as.factor(doy)09-16	0.070 (0.047)	−0.036 (0.059)
as.factor(doy)09-17	0.073 (0.047)	−0.049 (0.059)
as.factor(doy)09-18	0.028 (0.047)	−0.009 (0.059)
as.factor(doy)09-19	0.035 (0.047)	−0.093 (0.059)
as.factor(doy)09-20	0.051 (0.048)	−0.073 (0.060)
as.factor(doy)09-21	0.039 (0.047)	−0.045 (0.059)
as.factor(doy)09-22	0.094** (0.047)	−0.101* (0.059)
as.factor(doy)09-23	0.031 (0.047)	−0.013 (0.059)
as.factor(doy)09-24	0.025 (0.047)	−0.001 (0.059)
as.factor(doy)09-25	0.004 (0.047)	−0.042 (0.059)
as.factor(doy)09-26	0.063 (0.047)	−0.043 (0.059)
as.factor(doy)09-27	0.040 (0.047)	−0.091 (0.059)
as.factor(doy)09-28	0.051 (0.047)	−0.045 (0.059)
as.factor(doy)09-29	0.060 (0.047)	−0.079 (0.059)
as.factor(doy)09-30	0.027 (0.047)	−0.078 (0.059)
as.factor(doy)10-01	0.004 (0.047)	−0.044 (0.059)
as.factor(doy)10-02	0.019 (0.047)	−0.054 (0.059)
as.factor(doy)10-03	−0.016 (0.047)	−0.030 (0.059)
as.factor(doy)10-04	0.002 (0.047)	−0.046 (0.059)
as.factor(doy)10-05	0.041 (0.047)	−0.103* (0.059)
as.factor(doy)10-06	0.014 (0.047)	−0.038 (0.059)
as.factor(doy)10-07	0.031 (0.047)	−0.047 (0.059)
as.factor(doy)10-08	0.005 (0.047)	−0.081 (0.059)
as.factor(doy)10-09	−0.034 (0.047)	−0.070 (0.059)
as.factor(doy)10-10	0.012 (0.047)	−0.039 (0.059)
as.factor(doy)10-11	−0.021 (0.047)	−0.137** (0.059)

as.factor(doy)10-12	−0.029 (0.047)	−0.094 (0.059)
as.factor(doy)10-13	0.013 (0.047)	−0.067 (0.059)
as.factor(doy)10-14	0.061 (0.047)	−0.142** (0.059)
as.factor(doy)10-15	0.061 (0.047)	−0.044 (0.059)
as.factor(doy)10-16	0.017 (0.047)	−0.125** (0.059)
as.factor(doy)10-17	−0.106** (0.047)	−0.081 (0.059)
as.factor(doy)10-18	−0.017 (0.047)	−0.081 (0.059)
as.factor(doy)10-19	−0.037 (0.047)	−0.092 (0.059)
as.factor(doy)10-20	−0.047 (0.047)	−0.109* (0.059)
as.factor(doy)10-21	−0.015 (0.050)	−0.058 (0.062)
as.factor(doy)10-22	0.030 (0.050)	−0.040 (0.062)
as.factor(doy)10-23	−0.010 (0.050)	−0.083 (0.062)
as.factor(doy)10-24	0.016 (0.050)	−0.108* (0.063)
as.factor(doy)10-25	−0.059 (0.050)	−0.101 (0.063)
as.factor(doy)10-26	−0.023 (0.050)	−0.076 (0.062)
as.factor(doy)10-27	−0.047 (0.050)	−0.086 (0.062)
as.factor(doy)10-28	−0.071 (0.050)	−0.105* (0.062)
as.factor(doy)10-29	0.017 (0.050)	−0.064 (0.062)
as.factor(doy)10-30	−0.030 (0.050)	−0.119* (0.062)
as.factor(doy)10-31	−0.114** (0.050)	−0.181*** (0.062)
as.factor(doy)11-01	−0.034 (0.050)	−0.043 (0.062)
as.factor(doy)11-02	−0.032 (0.050)	−0.076 (0.062)
as.factor(doy)11-03	−0.044 (0.050)	−0.088 (0.062)
as.factor(doy)11-04	−0.044 (0.050)	−0.097 (0.062)
as.factor(doy)11-05	−0.042 (0.050)	−0.090 (0.062)
as.factor(doy)11-06	0.026 (0.050)	0.022 (0.062)
as.factor(doy)11-07	−0.107** (0.050)	−0.108* (0.062)
as.factor(doy)11-08	−0.102** (0.050)	−0.116* (0.062)
as.factor(doy)11-09	−0.001 (0.050)	−0.145** (0.062)
as.factor(doy)11-10	0.017 (0.050)	−0.121* (0.062)
as.factor(doy)11-11	−0.044 (0.050)	−0.132** (0.062)
as.factor(doy)11-12	−0.049 (0.050)	−0.069 (0.062)
as.factor(doy)11-13	−0.047 (0.050)	−0.090 (0.062)
as.factor(doy)11-14	−0.073 (0.050)	−0.087 (0.062)
as.factor(doy)11-15	−0.059 (0.050)	−0.057 (0.063)
as.factor(doy)11-16	−0.099** (0.050)	−0.205*** (0.063)

as.factor(doy)11-17	-0.046 (0.050)	-0.062 (0.062)
as.factor(doy)11-18	-0.053 (0.050)	-0.096 (0.062)
as.factor(doy)11-19	-0.037 (0.050)	-0.087 (0.062)
as.factor(doy)11-20	-0.056 (0.050)	-0.138^{**} (0.062)
as.factor(doy)11-21	-0.119^{**} (0.050)	-0.135^{**} (0.062)
as.factor(doy)11-22	-0.067 (0.050)	-0.124^{**} (0.062)
as.factor(doy)11-23	-0.105^{**} (0.050)	-0.132^{**} (0.062)
as.factor(doy)11-24	-0.063 (0.050)	-0.108^{*} (0.062)
as.factor(doy)11-25	-0.064 (0.050)	-0.121^{*} (0.062)
as.factor(doy)11-26	-0.030 (0.050)	-0.096 (0.062)
as.factor(doy)11-27	-0.002 (0.050)	-0.130^{**} (0.062)
as.factor(doy)11-28	-0.116^{**} (0.050)	-0.148^{**} (0.062)
as.factor(doy)11-29	-0.096^{*} (0.050)	-0.124^{**} (0.062)
as.factor(doy)11-30	-0.107^{**} (0.050)	-0.163^{***} (0.062)
as.factor(doy)12-01	-0.061 (0.050)	-0.140^{**} (0.062)
as.factor(doy)12-02	-0.075 (0.050)	-0.126^{**} (0.062)
as.factor(doy)12-03	-0.169^{***} (0.050)	-0.160^{**} (0.062)
as.factor(doy)12-04	-0.071 (0.050)	-0.056 (0.062)
as.factor(doy)12-05	-0.101^{**} (0.050)	-0.134^{**} (0.062)
as.factor(doy)12-06	-0.075 (0.050)	-0.134^{**} (0.062)
as.factor(doy)12-07	-0.105^{**} (0.050)	-0.122^{*} (0.062)
as.factor(doy)12-08	-0.155^{***} (0.050)	-0.124^{**} (0.062)
as.factor(doy)12-09	-0.123^{**} (0.050)	-0.099 (0.062)
as.factor(doy)12-10	-0.085^{*} (0.050)	-0.090 (0.062)
as.factor(doy)12-11	-0.083^{*} (0.050)	-0.018 (0.062)
as.factor(doy)12-12	-0.126^{**} (0.050)	-0.116^{*} (0.062)
as.factor(doy)12-13	-0.183^{***} (0.050)	-0.146^{**} (0.062)
as.factor(doy)12-14	-0.119^{**} (0.050)	-0.140^{**} (0.062)
as.factor(doy)12-15	-0.103^{**} (0.050)	-0.133^{**} (0.062)
as.factor(doy)12-16	-0.087^{*} (0.050)	-0.126^{**} (0.062)
as.factor(doy)12-17	-0.068 (0.050)	-0.126^{**} (0.062)
as.factor(doy)12-18	-0.090^{*} (0.050)	-0.193^{***} (0.062)
as.factor(doy)12-19	-0.099^{**} (0.050)	-0.214^{***} (0.062)
as.factor(doy)12-20	-0.082 (0.050)	-0.241^{***} (0.062)
as.factor(doy)12-21	-0.091^{*} (0.050)	-0.101 (0.063)
as.factor(doy)12-22	-0.005 (0.050)	-0.095 (0.062)

as.factor(doy)12-23	0.004 (0.050)	−0.021 (0.062)
as.factor(doy)12-24	−0.169*** (0.050)	−0.233*** (0.062)
as.factor(doy)12-25	−0.429*** (0.050)	−0.454*** (0.062)
as.factor(doy)12-26	0.164*** (0.054)	−0.026 (0.067)
as.factor(doy)12-27	0.136** (0.054)	−0.068 (0.067)
as.factor(doy)12-28	0.124** (0.054)	−0.020 (0.068)
as.factor(doy)12-29	0.148*** (0.054)	0.097 (0.068)
as.factor(doy)12-30	0.189*** (0.054)	0.034 (0.067)
as.factor(doy)12-31	−0.023 (0.054)	−0.118* (0.067)
Constant	3.050*** (0.087)	2.318*** (0.109)
Observations	21,011	20,781
R^2	0.694	0.913
Adjusted R^2	0.688	0.911
Residual Std. Error	2.102 (df = 20561)	2.630 (df = 20331)
F Statistic	104.057*** (df = 449; 20561)	476.239*** (df = 449; 20331)

Notes: ***Significant at the 1 percent level.
**Significant at the 5 percent level.
*Significant at the 10 percent level.

Table 5: July 31 - Event Study

	log(triage45)	log(triage123)
	(1)	(2)
DkEventlower_bin	0.144* (0.080)	0.257** (0.101)
DkEvent-10	0.064 (0.112)	0.176 (0.140)
DkEvent-9	−0.074 (0.112)	0.161 (0.140)
DkEvent-8	0.076 (0.112)	0.045 (0.140)
DkEvent-7	0.006 (0.111)	0.121 (0.139)
DkEvent-6	−0.052 (0.112)	0.193 (0.140)
DkEvent-5	0.111 (0.112)	0.200 (0.140)
DkEvent-4	0.109 (0.112)	0.294** (0.140)
DkEvent-3	0.121 (0.112)	0.299** (0.140)
DkEvent-2	0.135 (0.112)	0.169 (0.140)
DkEvent-1	−0.003 (0.112)	0.087 (0.140)
DkEvent1	−0.039 (0.112)	0.322** (0.140)

DkEvent2	0.041 (0.112)	0.041 (0.140)
DkEvent3	0.165 (0.112)	0.262* (0.140)
DkEvent4	0.109 (0.112)	0.138 (0.140)
DkEvent5	0.024 (0.112)	0.183 (0.140)
DkEvent6	0.058 (0.112)	0.166 (0.140)
DkEvent7	−0.050 (0.111)	0.084 (0.139)
DkEvent8	−0.002 (0.112)	0.195 (0.140)
DkEvent9	0.063 (0.112)	0.165 (0.140)
DkEvent10	0.101 (0.112)	0.164 (0.140)
DkEvent11	0.135 (0.112)	0.290** (0.140)
DkEvent12	0.076 (0.112)	0.109 (0.140)
DkEvent13	0.092 (0.112)	0.077 (0.140)
DkEvent14	0.134 (0.112)	0.029 (0.141)
DkEvent15	0.138 (0.115)	0.176 (0.144)
DkEvent16	0.151 (0.112)	0.198 (0.141)
DkEvent17	0.080 (0.112)	0.188 (0.140)
DkEvent18	0.160 (0.112)	0.174 (0.140)
DkEvent19	0.269** (0.112)	0.326** (0.140)
DkEvent20	0.072 (0.112)	0.244* (0.140)
DkEvent21	0.097 (0.111)	0.047 (0.139)
DkEvent22	0.064 (0.112)	0.095 (0.140)
DkEvent23	0.190* (0.113)	0.361** (0.142)
DkEvent24	0.124 (0.112)	0.174 (0.140)
DkEvent25	0.012 (0.112)	0.262* (0.140)
DkEvent26	0.107 (0.112)	0.199 (0.140)
DkEvent27	0.185* (0.112)	−0.052 (0.140)
DkEvent28	−0.016 (0.112)	−0.074 (0.141)
DkEvent29	−0.015 (0.113)	0.151 (0.141)
DkEvent30	−0.050 (0.112)	0.296** (0.141)
DkEvent31	0.022 (0.112)	0.200 (0.140)
DkEvent32	−0.051 (0.112)	0.082 (0.140)
DkEvent33	−0.148 (0.112)	0.240* (0.140)
DkEvent34	−0.035 (0.112)	0.065 (0.140)
DkEvent35	−0.112 (0.111)	0.029 (0.140)
DkEvent36	−0.197* (0.112)	0.018 (0.140)
DkEvent37	−0.013 (0.112)	0.040 (0.140)

DkEvent38	0.177 (0.113)	0.348** (0.142)
DkEvent39	−0.054 (0.112)	0.202 (0.140)
DkEvent40	0.086 (0.112)	0.195 (0.140)
DkEvent41	−0.078 (0.112)	0.182 (0.140)
DkEvent42	−0.002 (0.111)	0.120 (0.139)
DkEvent43	−0.107 (0.112)	0.124 (0.140)
DkEvent44	0.294** (0.119)	0.217 (0.149)
DkEvent45	0.077 (0.113)	0.171 (0.142)
DkEvent46	0.176 (0.114)	0.089 (0.142)
DkEvent47	0.061 (0.113)	0.187 (0.142)
DkEvent48	−0.128 (0.114)	0.124 (0.142)
DkEvent49	0.015 (0.111)	0.212 (0.139)
DkEvent50	−0.034 (0.112)	0.028 (0.140)
DkEventupper_bin	−0.002 (0.086)	0.155 (0.108)
lag(DailyTotals, n = 1)	−0.002*** (0.000)	−0.002*** (0.000)
TotalVaccinated	0.000*** (0.000)	0.000*** (0.000)
EDFacility1	0.646*** (0.009)	1.782*** (0.011)
EDFacility2	−1.128*** (0.015)	−1.604*** (0.020)
EDFacility3	−0.211*** (0.011)	−0.376*** (0.014)
EDFacility4	0.205*** (0.009)	1.305*** (0.012)
EDFacility5	0.452*** (0.009)	2.022*** (0.011)
EDFacility6	0.625*** (0.009)	0.378*** (0.012)
EDFacility7	−0.017 (0.011)	−0.841*** (0.014)
EDFacility8	0.522*** (0.009)	2.135*** (0.011)
EDFacility9	0.495*** (0.010)	0.430*** (0.012)
EDFacility10	0.188*** (0.010)	0.232*** (0.013)
EDFacility11	0.305*** (0.010)	0.682*** (0.012)
as.factor(yr)2018	−0.041*** (0.005)	0.019*** (0.007)
as.factor(yr)2019	−0.116*** (0.005)	0.020*** (0.007)
as.factor(yr)2020	−0.418*** (0.005)	−0.201*** (0.007)
as.factor(yr)2021	−0.471*** (0.009)	−0.147*** (0.012)
as.factor(dow)monday	0.085*** (0.007)	0.082*** (0.008)
as.factor(dow)saturday	−0.059*** (0.007)	−0.058*** (0.008)
as.factor(dow)sunday	−0.040*** (0.007)	−0.052*** (0.008)
as.factor(dow)thursday	−0.001 (0.007)	0.002 (0.008)
as.factor(dow)tuesday	0.012* (0.007)	0.014* (0.008)

as.factor(dow)wednesday	−0.000 (0.007)	0.013 (0.008)
as.factor(doy)01-02	0.197*** (0.045)	0.029 (0.056)
as.factor(doy)01-03	0.139*** (0.045)	0.097* (0.056)
as.factor(doy)01-04	0.042 (0.045)	−0.029 (0.056)
as.factor(doy)01-05	0.086* (0.045)	0.007 (0.056)
as.factor(doy)01-06	0.092** (0.045)	−0.033 (0.056)
as.factor(doy)01-07	0.020 (0.045)	−0.005 (0.056)
as.factor(doy)01-08	−0.006 (0.045)	−0.119** (0.056)
as.factor(doy)01-09	−0.017 (0.045)	−0.069 (0.056)
as.factor(doy)01-10	−0.002 (0.045)	−0.086 (0.056)
as.factor(doy)01-11	0.026 (0.045)	−0.070 (0.056)
as.factor(doy)01-12	−0.035 (0.045)	−0.069 (0.056)
as.factor(doy)01-13	0.015 (0.045)	−0.100* (0.056)
as.factor(doy)01-14	−0.024 (0.045)	−0.081 (0.056)
as.factor(doy)01-15	0.017 (0.045)	−0.053 (0.056)
as.factor(doy)01-16	−0.039 (0.045)	−0.025 (0.056)
as.factor(doy)01-17	−0.025 (0.045)	−0.116** (0.056)
as.factor(doy)01-18	0.015 (0.045)	−0.047 (0.056)
as.factor(doy)01-19	−0.018 (0.045)	−0.080 (0.056)
as.factor(doy)01-20	−0.099** (0.045)	−0.203*** (0.056)
as.factor(doy)01-21	−0.037 (0.045)	−0.044 (0.056)
as.factor(doy)01-22	0.037 (0.045)	−0.035 (0.056)
as.factor(doy)01-23	−0.021 (0.045)	−0.086 (0.056)
as.factor(doy)01-24	−0.086* (0.045)	−0.085 (0.056)
as.factor(doy)01-25	0.004 (0.045)	−0.018 (0.056)
as.factor(doy)01-26	0.067 (0.045)	−0.067 (0.056)
as.factor(doy)01-27	0.070 (0.045)	0.041 (0.056)
as.factor(doy)01-28	0.075* (0.045)	0.011 (0.056)
as.factor(doy)01-29	0.075* (0.045)	0.005 (0.056)
as.factor(doy)01-30	0.003 (0.045)	0.013 (0.056)
as.factor(doy)01-31	0.031 (0.045)	−0.034 (0.056)
as.factor(doy)02-01	0.086* (0.045)	−0.026 (0.056)
as.factor(doy)02-02	−0.071 (0.045)	−0.134** (0.056)
as.factor(doy)02-03	0.075* (0.045)	0.001 (0.056)
as.factor(doy)02-04	0.069 (0.045)	0.029 (0.056)
as.factor(doy)02-05	0.077* (0.045)	0.086 (0.056)

as.factor(doy)02-06	0.055 (0.045)	0.036 (0.056)
as.factor(doy)02-07	−0.015 (0.045)	−0.048 (0.056)
as.factor(doy)02-08	−0.008 (0.045)	−0.042 (0.056)
as.factor(doy)02-09	0.075* (0.045)	−0.017 (0.056)
as.factor(doy)02-10	−0.015 (0.045)	−0.031 (0.056)
as.factor(doy)02-11	−0.012 (0.045)	−0.020 (0.056)
as.factor(doy)02-12	0.125*** (0.045)	0.038 (0.056)
as.factor(doy)02-13	−0.239*** (0.045)	−0.278*** (0.056)
as.factor(doy)02-14	0.014 (0.045)	0.045 (0.056)
as.factor(doy)02-15	0.080* (0.045)	−0.002 (0.056)
as.factor(doy)02-16	−0.065 (0.045)	−0.071 (0.056)
as.factor(doy)02-17	0.074* (0.045)	0.040 (0.056)
as.factor(doy)02-18	0.125*** (0.045)	0.072 (0.056)
as.factor(doy)02-19	0.140*** (0.045)	0.067 (0.056)
as.factor(doy)02-20	0.132*** (0.045)	0.050 (0.056)
as.factor(doy)02-21	0.055 (0.045)	0.063 (0.056)
as.factor(doy)02-22	0.091** (0.045)	0.069 (0.056)
as.factor(doy)02-23	0.096** (0.045)	0.035 (0.056)
as.factor(doy)02-24	0.056 (0.045)	0.071 (0.056)
as.factor(doy)02-25	0.084* (0.045)	0.039 (0.056)
as.factor(doy)02-26	0.079* (0.045)	0.032 (0.056)
as.factor(doy)02-27	0.034 (0.045)	−0.050 (0.056)
as.factor(doy)02-28	0.035 (0.045)	0.077 (0.056)
as.factor(doy)02-29	0.258*** (0.077)	0.212** (0.097)
as.factor(doy)03-01	0.073 (0.045)	−0.021 (0.056)
as.factor(doy)03-02	0.004 (0.045)	0.020 (0.056)
as.factor(doy)03-03	0.115*** (0.045)	0.026 (0.056)
as.factor(doy)03-04	0.018 (0.045)	−0.037 (0.056)
as.factor(doy)03-05	0.063 (0.045)	0.045 (0.056)
as.factor(doy)03-06	0.073 (0.045)	0.015 (0.056)
as.factor(doy)03-07	0.053 (0.045)	0.005 (0.056)
as.factor(doy)03-08	0.035 (0.045)	−0.087 (0.056)
as.factor(doy)03-09	0.093** (0.045)	0.058 (0.056)
as.factor(doy)03-10	0.043 (0.045)	0.052 (0.056)
as.factor(doy)03-11	0.039 (0.045)	0.026 (0.056)
as.factor(doy)03-12	0.106** (0.045)	0.064 (0.056)

as.factor(doy)03-13	0.092** (0.045)	−0.043 (0.056)
as.factor(doy)03-14	−0.050 (0.045)	−0.077 (0.056)
as.factor(doy)03-15	−0.003 (0.045)	−0.033 (0.056)
as.factor(doy)03-16	0.013 (0.045)	−0.028 (0.056)
as.factor(doy)03-17	−0.049 (0.045)	−0.028 (0.056)
as.factor(doy)03-18	−0.014 (0.045)	−0.059 (0.056)
as.factor(doy)03-19	−0.035 (0.045)	−0.074 (0.056)
as.factor(doy)03-20	−0.097** (0.045)	−0.137** (0.056)
as.factor(doy)03-21	−0.031 (0.045)	−0.138** (0.056)
as.factor(doy)03-22	−0.133*** (0.045)	−0.177*** (0.056)
as.factor(doy)03-23	−0.054 (0.045)	−0.161*** (0.056)
as.factor(doy)03-24	−0.049 (0.045)	−0.136** (0.056)
as.factor(doy)03-25	−0.066 (0.045)	−0.083 (0.056)
as.factor(doy)03-26	−0.046 (0.045)	−0.122** (0.056)
as.factor(doy)03-27	−0.122*** (0.045)	−0.186*** (0.056)
as.factor(doy)03-28	−0.116*** (0.045)	−0.142** (0.056)
as.factor(doy)03-29	−0.084* (0.045)	−0.053 (0.056)
as.factor(doy)03-30	−0.079* (0.045)	−0.179*** (0.056)
as.factor(doy)03-31	−0.076* (0.045)	−0.136** (0.056)
as.factor(doy)04-01	−0.085* (0.045)	−0.178*** (0.056)
as.factor(doy)04-02	−0.128*** (0.045)	−0.128** (0.056)
as.factor(doy)04-03	−0.083* (0.045)	−0.200*** (0.056)
as.factor(doy)04-04	−0.146*** (0.045)	−0.189*** (0.056)
as.factor(doy)04-05	−0.136*** (0.045)	−0.074 (0.056)
as.factor(doy)04-06	−0.128*** (0.045)	−0.135** (0.056)
as.factor(doy)04-07	−0.057 (0.045)	−0.142** (0.056)
as.factor(doy)04-08	−0.206*** (0.045)	−0.133** (0.056)
as.factor(doy)04-09	−0.114** (0.045)	−0.162*** (0.056)
as.factor(doy)04-10	−0.142*** (0.045)	−0.119** (0.056)
as.factor(doy)04-11	−0.060 (0.045)	−0.133** (0.056)
as.factor(doy)04-12	−0.112** (0.045)	−0.180*** (0.056)
as.factor(doy)04-13	−0.109** (0.045)	−0.114** (0.056)
as.factor(doy)04-14	−0.073 (0.045)	−0.149*** (0.056)
as.factor(doy)04-15	−0.078* (0.045)	−0.095* (0.056)
as.factor(doy)04-16	−0.142*** (0.045)	−0.147*** (0.056)
as.factor(doy)04-17	−0.085* (0.045)	−0.142** (0.056)

as.factor(doy)04-18	−0.072 (0.045)	−0.157*** (0.056)
as.factor(doy)04-19	−0.083* (0.045)	−0.107* (0.056)
as.factor(doy)04-20	−0.113** (0.045)	−0.113** (0.056)
as.factor(doy)04-21	−0.038 (0.045)	−0.119** (0.056)
as.factor(doy)04-22	−0.069 (0.045)	−0.090 (0.056)
as.factor(doy)04-23	−0.038 (0.045)	−0.086 (0.056)
as.factor(doy)04-24	−0.060 (0.045)	−0.129** (0.056)
as.factor(doy)04-25	−0.093** (0.045)	−0.108* (0.056)
as.factor(doy)04-26	−0.070 (0.045)	−0.116** (0.056)
as.factor(doy)04-27	−0.039 (0.045)	−0.103* (0.056)
as.factor(doy)04-28	−0.103** (0.045)	−0.138** (0.056)
as.factor(doy)04-29	0.005 (0.045)	−0.086 (0.056)
as.factor(doy)04-30	−0.015 (0.045)	−0.134** (0.056)
as.factor(doy)05-01	−0.021 (0.045)	−0.108* (0.056)
as.factor(doy)05-02	−0.025 (0.045)	−0.107* (0.056)
as.factor(doy)05-03	0.026 (0.045)	0.004 (0.056)
as.factor(doy)05-04	−0.016 (0.045)	−0.063 (0.056)
as.factor(doy)05-05	−0.005 (0.045)	−0.056 (0.056)
as.factor(doy)05-06	−0.042 (0.045)	−0.030 (0.056)
as.factor(doy)05-07	0.002 (0.045)	−0.061 (0.056)
as.factor(doy)05-08	−0.044 (0.045)	−0.093* (0.056)
as.factor(doy)05-09	−0.131*** (0.045)	−0.127** (0.056)
as.factor(doy)05-10	−0.050 (0.045)	−0.047 (0.056)
as.factor(doy)05-11	−0.046 (0.045)	−0.062 (0.056)
as.factor(doy)05-12	−0.026 (0.045)	−0.133** (0.056)
as.factor(doy)05-13	−0.078* (0.045)	−0.044 (0.056)
as.factor(doy)05-14	−0.039 (0.045)	−0.132** (0.056)
as.factor(doy)05-15	−0.046 (0.045)	−0.063 (0.056)
as.factor(doy)05-16	0.007 (0.045)	−0.093* (0.056)
as.factor(doy)05-17	−0.039 (0.045)	−0.076 (0.056)
as.factor(doy)05-18	−0.036 (0.045)	−0.061 (0.056)
as.factor(doy)05-19	0.054 (0.045)	−0.075 (0.056)
as.factor(doy)05-20	0.043 (0.045)	−0.059 (0.056)
as.factor(doy)05-21	0.013 (0.045)	−0.048 (0.056)
as.factor(doy)05-22	−0.008 (0.045)	−0.115** (0.056)
as.factor(doy)05-23	0.030 (0.045)	−0.032 (0.056)

as.factor(doy)05-24	−0.039 (0.045)	−0.048 (0.056)
as.factor(doy)05-25	0.032 (0.045)	−0.042 (0.056)
as.factor(doy)05-26	0.031 (0.045)	−0.074 (0.056)
as.factor(doy)05-27	−0.013 (0.045)	−0.025 (0.056)
as.factor(doy)05-28	0.030 (0.045)	−0.057 (0.056)
as.factor(doy)05-29	−0.012 (0.045)	−0.060 (0.056)
as.factor(doy)05-30	−0.031 (0.045)	−0.067 (0.056)
as.factor(doy)05-31	−0.052 (0.045)	−0.079 (0.056)
as.factor(doy)06-01	−0.035 (0.045)	−0.070 (0.056)
as.factor(doy)06-02	−0.008 (0.045)	-0.106^{*} (0.056)
as.factor(doy)06-03	0.013 (0.045)	−0.020 (0.056)
as.factor(doy)06-04	0.042 (0.045)	−0.082 (0.056)
as.factor(doy)06-05	0.001 (0.045)	−0.061 (0.056)
as.factor(doy)06-06	0.038 (0.045)	−0.051 (0.056)
as.factor(doy)06-07	−0.035 (0.045)	−0.056 (0.056)
as.factor(doy)06-08	−0.031 (0.045)	−0.050 (0.056)
as.factor(doy)06-09	−0.006 (0.045)	−0.078 (0.056)
as.factor(doy)06-10	0.022 (0.045)	−0.026 (0.056)
as.factor(doy)06-11	0.006 (0.045)	-0.121^{**} (0.056)
as.factor(doy)06-12	−0.014 (0.045)	−0.085 (0.056)
as.factor(doy)06-13	0.009 (0.045)	−0.077 (0.056)
as.factor(doy)06-14	−0.010 (0.045)	−0.071 (0.056)
as.factor(doy)06-15	0.035 (0.045)	−0.081 (0.056)
as.factor(doy)06-16	−0.009 (0.045)	-0.123^{**} (0.056)
as.factor(doy)06-17	−0.022 (0.045)	-0.135^{**} (0.056)
as.factor(doy)06-18	0.012 (0.045)	−0.065 (0.056)
as.factor(doy)06-19	0.048 (0.045)	-0.120^{**} (0.056)
as.factor(doy)06-20	−0.016 (0.045)	-0.124^{**} (0.056)
as.factor(doy)06-21	-0.078^{*} (0.045)	−0.072 (0.056)
as.factor(doy)06-22	0.009 (0.045)	-0.100^{*} (0.056)
as.factor(doy)06-23	0.039 (0.045)	−0.091 (0.056)
as.factor(doy)06-24	0.010 (0.045)	-0.117^{**} (0.056)
as.factor(doy)06-25	0.012 (0.045)	−0.061 (0.056)
as.factor(doy)06-26	0.049 (0.045)	-0.096^{*} (0.056)
as.factor(doy)06-27	−0.008 (0.045)	-0.094^{*} (0.056)
as.factor(doy)06-28	0.047 (0.045)	-0.122^{**} (0.056)

as.factor(doy)06-29	-0.007 (0.045)	-0.099^{*} (0.056)
as.factor(doy)06-30	0.065 (0.045)	-0.075 (0.056)
as.factor(doy)07-01	-0.005 (0.045)	-0.189^{***} (0.056)
as.factor(doy)07-02	0.093^{**} (0.045)	-0.034 (0.056)
as.factor(doy)07-03	0.079^{*} (0.045)	-0.035 (0.056)
as.factor(doy)07-04	0.031 (0.045)	-0.128^{**} (0.056)
as.factor(doy)07-05	0.018 (0.045)	-0.048 (0.056)
as.factor(doy)07-06	-0.002 (0.045)	-0.096^{*} (0.056)
as.factor(doy)07-07	0.058 (0.045)	-0.088 (0.056)
as.factor(doy)07-08	0.027 (0.045)	-0.042 (0.056)
as.factor(doy)07-09	-0.009 (0.045)	-0.026 (0.056)
as.factor(doy)07-10	0.046 (0.045)	-0.030 (0.056)
as.factor(doy)07-11	-0.021 (0.045)	-0.088 (0.056)
as.factor(doy)07-12	0.050 (0.045)	-0.052 (0.056)
as.factor(doy)07-13	0.065 (0.045)	-0.084 (0.056)
as.factor(doy)07-14	0.068 (0.045)	-0.083 (0.056)
as.factor(doy)07-15	0.059 (0.045)	-0.064 (0.056)
as.factor(doy)07-16	-0.012 (0.045)	-0.031 (0.056)
as.factor(doy)07-17	-0.019 (0.045)	-0.084 (0.056)
as.factor(doy)07-18	0.002 (0.045)	-0.113^{**} (0.056)
as.factor(doy)07-19	-0.008 (0.045)	-0.050 (0.056)
as.factor(doy)07-20	0.049 (0.045)	-0.073 (0.056)
as.factor(doy)07-21	0.063 (0.047)	-0.092 (0.059)
as.factor(doy)07-22	0.134^{***} (0.047)	-0.051 (0.059)
as.factor(doy)07-23	0.119^{**} (0.047)	-0.052 (0.060)
as.factor(doy)07-24	0.083^{*} (0.047)	-0.061 (0.059)
as.factor(doy)07-25	0.088^{*} (0.047)	-0.046 (0.059)
as.factor(doy)07-26	0.071 (0.047)	-0.045 (0.059)
as.factor(doy)07-27	0.058 (0.047)	-0.087 (0.059)
as.factor(doy)07-28	0.085^{*} (0.047)	-0.044 (0.059)
as.factor(doy)07-29	0.116^{**} (0.047)	-0.060 (0.059)
as.factor(doy)07-30	0.086^{*} (0.047)	-0.070 (0.059)
as.factor(doy)07-31	0.093^{*} (0.047)	-0.028 (0.059)
as.factor(doy)08-01	0.070 (0.047)	-0.174^{***} (0.059)
as.factor(doy)08-02	0.056 (0.047)	-0.032 (0.059)
as.factor(doy)08-03	0.113^{**} (0.047)	-0.115^{*} (0.059)

as.factor(doy)08-04	0.059 (0.047)	0.022 (0.059)
as.factor(doy)08-05	0.092* (0.047)	−0.068 (0.059)
as.factor(doy)08-06	0.122** (0.047)	−0.050 (0.059)
as.factor(doy)08-07	0.120** (0.047)	−0.046 (0.059)
as.factor(doy)08-08	0.127*** (0.047)	−0.048 (0.059)
as.factor(doy)08-09	0.063 (0.047)	−0.040 (0.059)
as.factor(doy)08-10	0.034 (0.047)	−0.067 (0.059)
as.factor(doy)08-11	0.028 (0.047)	−0.055 (0.059)
as.factor(doy)08-12	0.078* (0.047)	−0.027 (0.059)
as.factor(doy)08-13	0.075 (0.047)	−0.064 (0.059)
as.factor(doy)08-14	0.019 (0.047)	−0.056 (0.059)
as.factor(doy)08-15	0.047 (0.047)	−0.148** (0.059)
as.factor(doy)08-16	0.032 (0.047)	−0.044 (0.059)
as.factor(doy)08-17	0.032 (0.047)	−0.075 (0.059)
as.factor(doy)08-18	0.003 (0.047)	−0.039 (0.059)
as.factor(doy)08-19	0.014 (0.047)	−0.089 (0.059)
as.factor(doy)08-20	0.023 (0.047)	−0.090 (0.059)
as.factor(doy)08-21	−0.002 (0.047)	−0.015 (0.059)
as.factor(doy)08-22	0.037 (0.047)	−0.084 (0.059)
as.factor(doy)08-23	0.036 (0.047)	−0.056 (0.059)
as.factor(doy)08-24	0.056 (0.047)	−0.071 (0.059)
as.factor(doy)08-25	0.015 (0.047)	−0.055 (0.059)
as.factor(doy)08-26	−0.001 (0.047)	−0.055 (0.059)
as.factor(doy)08-27	0.004 (0.047)	0.005 (0.059)
as.factor(doy)08-28	−0.017 (0.047)	−0.054 (0.059)
as.factor(doy)08-29	−0.003 (0.047)	−0.130** (0.059)
as.factor(doy)08-30	0.007 (0.047)	−0.051 (0.060)
as.factor(doy)08-31	0.016 (0.047)	−0.072 (0.059)
as.factor(doy)09-01	0.028 (0.047)	−0.082 (0.059)
as.factor(doy)09-02	0.034 (0.047)	−0.146** (0.059)
as.factor(doy)09-03	0.002 (0.047)	−0.054 (0.059)
as.factor(doy)09-04	0.049 (0.047)	−0.046 (0.059)
as.factor(doy)09-05	0.040 (0.047)	−0.049 (0.059)
as.factor(doy)09-06	0.020 (0.047)	−0.045 (0.059)
as.factor(doy)09-07	−0.062 (0.047)	−0.109* (0.059)
as.factor(doy)09-08	0.084* (0.048)	−0.093 (0.059)

as.factor(doy)09-09	−0.015 (0.047)	−0.036 (0.060)
as.factor(doy)09-10	0.003 (0.047)	−0.053 (0.059)
as.factor(doy)09-11	−0.016 (0.047)	−0.044 (0.059)
as.factor(doy)09-12	0.039 (0.048)	−0.039 (0.059)
as.factor(doy)09-13	−0.017 (0.047)	0.056 (0.059)
as.factor(doy)09-14	0.004 (0.047)	−0.064 (0.059)
as.factor(doy)09-15	−0.005 (0.048)	0.005 (0.059)
as.factor(doy)09-16	0.050 (0.047)	−0.021 (0.060)
as.factor(doy)09-17	0.092* (0.047)	−0.020 (0.059)
as.factor(doy)09-18	0.019 (0.047)	−0.003 (0.059)
as.factor(doy)09-19	0.035 (0.047)	−0.050 (0.059)
as.factor(doy)09-20	0.043 (0.045)	−0.038 (0.057)
as.factor(doy)09-21	0.032 (0.045)	−0.021 (0.056)
as.factor(doy)09-22	0.087* (0.045)	−0.077 (0.056)
as.factor(doy)09-23	0.024 (0.045)	0.008 (0.056)
as.factor(doy)09-24	0.018 (0.045)	0.024 (0.056)
as.factor(doy)09-25	−0.003 (0.045)	−0.019 (0.056)
as.factor(doy)09-26	0.056 (0.045)	−0.018 (0.056)
as.factor(doy)09-27	0.031 (0.045)	−0.066 (0.056)
as.factor(doy)09-28	0.044 (0.045)	−0.023 (0.056)
as.factor(doy)09-29	0.052 (0.045)	−0.055 (0.056)
as.factor(doy)09-30	0.019 (0.045)	−0.054 (0.056)
as.factor(doy)10-01	−0.004 (0.045)	−0.019 (0.056)
as.factor(doy)10-02	0.011 (0.045)	−0.025 (0.056)
as.factor(doy)10-03	−0.025 (0.045)	−0.005 (0.056)
as.factor(doy)10-04	−0.007 (0.045)	−0.024 (0.056)
as.factor(doy)10-05	0.033 (0.045)	−0.079 (0.056)
as.factor(doy)10-06	0.005 (0.045)	−0.016 (0.056)
as.factor(doy)10-07	0.022 (0.045)	−0.021 (0.056)
as.factor(doy)10-08	−0.004 (0.045)	−0.052 (0.056)
as.factor(doy)10-09	−0.043 (0.045)	−0.043 (0.056)
as.factor(doy)10-10	0.003 (0.045)	−0.015 (0.056)
as.factor(doy)10-11	−0.030 (0.045)	−0.111** (0.056)
as.factor(doy)10-12	−0.038 (0.045)	−0.068 (0.056)
as.factor(doy)10-13	0.004 (0.045)	−0.044 (0.056)
as.factor(doy)10-14	0.051 (0.045)	−0.115** (0.056)

as.factor(doy)10-15	0.052 (0.045)	−0.020 (0.056)
as.factor(doy)10-16	0.008 (0.045)	−0.104* (0.056)
as.factor(doy)10-17	−0.115** (0.045)	−0.060 (0.056)
as.factor(doy)10-18	−0.027 (0.045)	−0.061 (0.056)
as.factor(doy)10-19	−0.046 (0.045)	−0.073 (0.056)
as.factor(doy)10-20	−0.057 (0.045)	−0.088 (0.056)
as.factor(doy)10-21	−0.029 (0.047)	−0.038 (0.059)
as.factor(doy)10-22	0.017 (0.047)	−0.016 (0.059)
as.factor(doy)10-23	−0.022 (0.047)	−0.060 (0.059)
as.factor(doy)10-24	0.003 (0.048)	−0.086 (0.060)
as.factor(doy)10-25	−0.073 (0.047)	−0.080 (0.060)
as.factor(doy)10-26	−0.037 (0.047)	−0.057 (0.060)
as.factor(doy)10-27	−0.062 (0.047)	−0.066 (0.059)
as.factor(doy)10-28	−0.085* (0.047)	−0.085 (0.059)
as.factor(doy)10-29	0.004 (0.047)	−0.041 (0.059)
as.factor(doy)10-30	−0.042 (0.047)	−0.096 (0.059)
as.factor(doy)10-31	−0.126*** (0.047)	−0.159*** (0.059)
as.factor(doy)11-01	−0.048 (0.047)	−0.023 (0.059)
as.factor(doy)11-02	−0.046 (0.047)	−0.058 (0.059)
as.factor(doy)11-03	−0.058 (0.047)	−0.068 (0.059)
as.factor(doy)11-04	−0.058 (0.047)	−0.077 (0.059)
as.factor(doy)11-05	−0.055 (0.047)	−0.066 (0.059)
as.factor(doy)11-06	0.014 (0.047)	0.045 (0.059)
as.factor(doy)11-07	−0.120** (0.047)	−0.086 (0.059)
as.factor(doy)11-08	−0.116** (0.047)	−0.095 (0.059)
as.factor(doy)11-09	−0.015 (0.047)	−0.126** (0.059)
as.factor(doy)11-10	0.003 (0.047)	−0.101* (0.059)
as.factor(doy)11-11	−0.058 (0.047)	−0.113* (0.059)
as.factor(doy)11-12	−0.062 (0.047)	−0.046 (0.059)
as.factor(doy)11-13	−0.059 (0.047)	−0.067 (0.059)
as.factor(doy)11-14	−0.086* (0.047)	−0.065 (0.060)
as.factor(doy)11-15	−0.073 (0.048)	−0.037 (0.060)
as.factor(doy)11-16	−0.114** (0.047)	−0.185*** (0.060)
as.factor(doy)11-17	−0.060 (0.047)	−0.042 (0.059)
as.factor(doy)11-18	−0.067 (0.047)	−0.075 (0.059)
as.factor(doy)11-19	−0.050 (0.047)	−0.063 (0.059)

as.factor(doy)11-20	-0.068 (0.047)	-0.114^{*} (0.059)
as.factor(doy)11-21	-0.132^{***} (0.048)	-0.110^{*} (0.060)
as.factor(doy)11-22	-0.081^{*} (0.047)	-0.103^{*} (0.059)
as.factor(doy)11-23	-0.119^{**} (0.047)	-0.111^{*} (0.059)
as.factor(doy)11-24	-0.077 (0.047)	-0.087 (0.059)
as.factor(doy)11-25	-0.078 (0.047)	-0.101^{*} (0.059)
as.factor(doy)11-26	-0.042 (0.047)	-0.072 (0.059)
as.factor(doy)11-27	-0.014 (0.047)	-0.105^{*} (0.059)
as.factor(doy)11-28	-0.128^{***} (0.047)	-0.126^{**} (0.059)
as.factor(doy)11-29	-0.110^{**} (0.047)	-0.103^{*} (0.059)
as.factor(doy)11-30	-0.122^{**} (0.047)	-0.144^{**} (0.059)
as.factor(doy)12-01	-0.075 (0.047)	-0.120^{**} (0.059)
as.factor(doy)12-02	-0.089^{*} (0.047)	-0.105^{*} (0.059)
as.factor(doy)12-03	-0.182^{***} (0.047)	-0.136^{**} (0.059)
as.factor(doy)12-04	-0.083^{*} (0.047)	-0.032 (0.059)
as.factor(doy)12-05	-0.114^{**} (0.047)	-0.112^{*} (0.060)
as.factor(doy)12-06	-0.090^{*} (0.047)	-0.114^{*} (0.059)
as.factor(doy)12-07	-0.119^{**} (0.047)	-0.103^{*} (0.059)
as.factor(doy)12-08	-0.169^{***} (0.047)	-0.104^{*} (0.059)
as.factor(doy)12-09	-0.137^{***} (0.047)	-0.079 (0.059)
as.factor(doy)12-10	-0.098^{**} (0.047)	-0.066 (0.059)
as.factor(doy)12-11	-0.095^{**} (0.047)	0.006 (0.059)
as.factor(doy)12-12	-0.138^{***} (0.047)	-0.094 (0.059)
as.factor(doy)12-13	-0.198^{***} (0.047)	-0.125^{**} (0.059)
as.factor(doy)12-14	-0.133^{***} (0.047)	-0.121^{**} (0.059)
as.factor(doy)12-15	-0.117^{**} (0.047)	-0.113^{*} (0.059)
as.factor(doy)12-16	-0.101^{**} (0.047)	-0.105^{*} (0.059)
as.factor(doy)12-17	-0.081^{*} (0.047)	-0.102^{*} (0.059)
as.factor(doy)12-18	-0.102^{**} (0.048)	-0.170^{***} (0.059)
as.factor(doy)12-19	-0.111^{**} (0.047)	-0.192^{***} (0.060)
as.factor(doy)12-20	-0.096^{**} (0.047)	-0.221^{***} (0.059)
as.factor(doy)12-21	-0.105^{**} (0.047)	-0.082 (0.060)
as.factor(doy)12-22	-0.019 (0.047)	-0.075 (0.059)
as.factor(doy)12-23	-0.010 (0.047)	-0.000 (0.059)
as.factor(doy)12-24	-0.182^{***} (0.047)	-0.209^{***} (0.059)
as.factor(doy)12-25	-0.442^{***} (0.047)	-0.431^{***} (0.060)

as.factor(doy)12-26	0.121** (0.047)	−0.008 (0.059)
as.factor(doy)12-27	0.105** (0.048)	−0.025 (0.059)
as.factor(doy)12-28	0.101** (0.048)	−0.007 (0.060)
as.factor(doy)12-29	0.120** (0.048)	0.098 (0.060)
as.factor(doy)12-30	0.144*** (0.047)	0.053 (0.059)
as.factor(doy)12-31	−0.046 (0.047)	−0.116* (0.059)
Constant	3.467*** (0.087)	2.291*** (0.109)
Observations	21,011	20,781
R^2	0.691	0.912
Adjusted R^2	0.684	0.910
Residual Std. Error	2.113 (df = 20560)	2.645 (df = 20330)
F Statistic	102.259*** (df = 450; 20560)	469.308*** (df = 450; 20330)

Notes: ***Significant at the 1 percent level.
**Significant at the 5 percent level.
*Significant at the 10 percent level.

Table 6: September 24 - Event Study

	log(triage45)	log(triage123)
	(1)	(2)
DkEventlower_bin	0.107 (0.083)	−0.082 (0.104)
DkEvent-10	0.120 (0.112)	−0.079 (0.140)
DkEvent-9	0.224** (0.112)	−0.153 (0.140)
DkEvent-8	0.108 (0.112)	−0.059 (0.140)
DkEvent-7	−0.080 (0.112)	−0.115 (0.140)
DkEvent-6	0.045 (0.115)	−0.056 (0.144)
DkEvent-5	−0.003 (0.115)	−0.245* (0.144)
DkEvent-4	0.433*** (0.119)	0.230 (0.149)
DkEvent-3	0.190* (0.112)	−0.009 (0.140)
DkEvent-2	0.167 (0.112)	0.171 (0.140)
DkEvent-1	0.180 (0.112)	−0.008 (0.140)
DkEvent1	0.009 (0.112)	−0.187 (0.140)
DkEvent2	0.034 (0.112)	−0.110 (0.140)
DkEvent3	0.084 (0.112)	−0.049 (0.140)
DkEvent4	−0.054 (0.112)	−0.064 (0.140)

DkEvent5	0.026 (0.112)	0.098 (0.140)
DkEvent6	0.154 (0.112)	−0.247* (0.141)
DkEvent7	0.050 (0.112)	−0.071 (0.140)
DkEvent8	0.026 (0.114)	0.012 (0.142)
DkEvent9	0.105 (0.112)	−0.051 (0.141)
DkEvent10	0.046 (0.112)	−0.123 (0.140)
DkEvent11	0.068 (0.112)	0.019 (0.140)
DkEvent12	0.108 (0.112)	−0.058 (0.140)
DkEvent13	0.027 (0.112)	0.034 (0.141)
DkEvent14	0.032 (0.113)	−0.028 (0.142)
DkEvent15	−0.066 (0.112)	−0.145 (0.140)
DkEvent16	−0.040 (0.112)	−0.352** (0.140)
DkEvent17	−0.040 (0.112)	−0.173 (0.140)
DkEvent18	0.242** (0.112)	0.027 (0.141)
DkEvent19	0.086 (0.112)	−0.101 (0.140)
DkEvent20	0.116 (0.113)	0.081 (0.142)
DkEvent21	−0.022 (0.112)	−0.080 (0.140)
DkEvent22	−0.097 (0.112)	−0.360** (0.140)
DkEvent23	−0.094 (0.112)	−0.146 (0.140)
DkEvent24	0.037 (0.112)	−0.106 (0.140)
DkEvent25	−0.036 (0.112)	−0.105 (0.140)
DkEvent26	0.066 (0.112)	−0.243* (0.140)
lag(DailyTotals, n = 1)	−0.002*** (0.000)	−0.002*** (0.000)
TotalVaccinated	0.000*** (0.000)	0.000*** (0.000)
EDFacility1	0.646*** (0.009)	1.783*** (0.011)
EDFacility2	−1.128*** (0.015)	−1.604*** (0.020)
EDFacility3	−0.211*** (0.011)	−0.376*** (0.014)
EDFacility4	0.205*** (0.009)	1.305*** (0.012)
EDFacility5	0.452*** (0.009)	2.022*** (0.011)
EDFacility6	0.625*** (0.009)	0.378*** (0.012)
EDFacility7	−0.017 (0.011)	−0.841*** (0.014)
EDFacility8	0.522*** (0.009)	2.135*** (0.011)
EDFacility9	0.495*** (0.010)	0.430*** (0.012)
EDFacility10	0.188*** (0.010)	0.232*** (0.013)
EDFacility11	0.305*** (0.010)	0.682*** (0.012)
as.factor(yr)2018	−0.041*** (0.005)	0.019*** (0.007)

as.factor(yr)2019	−0.116*** (0.005)	0.020*** (0.007)
as.factor(yr)2020	−0.418*** (0.005)	−0.200*** (0.007)
as.factor(yr)2021	−0.455*** (0.009)	−0.129*** (0.011)
as.factor(dow)monday	0.085*** (0.007)	0.085*** (0.008)
as.factor(dow)saturday	−0.059*** (0.007)	−0.059*** (0.008)
as.factor(dow)sunday	−0.042*** (0.007)	−0.048*** (0.008)
as.factor(dow)thursday	−0.002 (0.007)	0.004 (0.008)
as.factor(dow)tuesday	0.014** (0.007)	0.018** (0.008)
as.factor(dow)wednesday	−0.000 (0.007)	0.015* (0.008)
as.factor(doy)01-02	0.197*** (0.045)	0.030 (0.056)
as.factor(doy)01-03	0.140*** (0.045)	0.098* (0.056)
as.factor(doy)01-04	0.043 (0.045)	−0.027 (0.056)
as.factor(doy)01-05	0.088** (0.045)	0.011 (0.056)
as.factor(doy)01-06	0.094** (0.045)	−0.029 (0.056)
as.factor(doy)01-07	0.022 (0.045)	−0.002 (0.056)
as.factor(doy)01-08	−0.004 (0.045)	−0.118** (0.056)
as.factor(doy)01-09	−0.015 (0.045)	−0.066 (0.056)
as.factor(doy)01-10	−0.001 (0.045)	−0.085 (0.056)
as.factor(doy)01-11	0.028 (0.045)	−0.067 (0.056)
as.factor(doy)01-12	−0.033 (0.045)	−0.066 (0.056)
as.factor(doy)01-13	0.016 (0.045)	−0.098* (0.056)
as.factor(doy)01-14	−0.023 (0.045)	−0.079 (0.056)
as.factor(doy)01-15	0.019 (0.045)	−0.050 (0.056)
as.factor(doy)01-16	−0.037 (0.045)	−0.022 (0.056)
as.factor(doy)01-17	−0.022 (0.045)	−0.112** (0.056)
as.factor(doy)01-18	0.017 (0.045)	−0.043 (0.056)
as.factor(doy)01-19	−0.015 (0.045)	−0.077 (0.056)
as.factor(doy)01-20	−0.097** (0.045)	−0.200*** (0.056)
as.factor(doy)01-21	−0.035 (0.045)	−0.041 (0.056)
as.factor(doy)01-22	0.039 (0.045)	−0.032 (0.056)
as.factor(doy)01-23	−0.020 (0.045)	−0.084 (0.056)
as.factor(doy)01-24	−0.084* (0.045)	−0.082 (0.056)
as.factor(doy)01-25	0.006 (0.045)	−0.014 (0.056)
as.factor(doy)01-26	0.068 (0.045)	−0.065 (0.056)
as.factor(doy)01-27	0.072 (0.045)	0.043 (0.056)
as.factor(doy)01-28	0.077* (0.045)	0.014 (0.056)

as.factor(doy)01-29	0.076* (0.045)	0.007 (0.056)
as.factor(doy)01-30	0.004 (0.045)	0.014 (0.056)
as.factor(doy)01-31	0.033 (0.045)	−0.031 (0.056)
as.factor(doy)02-01	0.087* (0.045)	−0.024 (0.056)
as.factor(doy)02-02	−0.070 (0.045)	−0.130** (0.056)
as.factor(doy)02-03	0.076* (0.045)	0.003 (0.056)
as.factor(doy)02-04	0.070 (0.045)	0.031 (0.056)
as.factor(doy)02-05	0.078* (0.045)	0.087 (0.056)
as.factor(doy)02-06	0.056 (0.045)	0.038 (0.056)
as.factor(doy)02-07	−0.014 (0.045)	−0.047 (0.056)
as.factor(doy)02-08	−0.008 (0.045)	−0.041 (0.056)
as.factor(doy)02-09	0.076* (0.045)	−0.015 (0.056)
as.factor(doy)02-10	−0.014 (0.045)	−0.029 (0.056)
as.factor(doy)02-11	−0.012 (0.045)	−0.020 (0.056)
as.factor(doy)02-12	0.125*** (0.045)	0.039 (0.056)
as.factor(doy)02-13	−0.238*** (0.045)	−0.276*** (0.056)
as.factor(doy)02-14	0.015 (0.045)	0.046 (0.056)
as.factor(doy)02-15	0.080* (0.045)	−0.001 (0.056)
as.factor(doy)02-16	−0.065 (0.045)	−0.069 (0.056)
as.factor(doy)02-17	0.075* (0.045)	0.041 (0.056)
as.factor(doy)02-18	0.126*** (0.045)	0.072 (0.056)
as.factor(doy)02-19	0.140*** (0.045)	0.068 (0.056)
as.factor(doy)02-20	0.132*** (0.045)	0.051 (0.056)
as.factor(doy)02-21	0.056 (0.045)	0.064 (0.056)
as.factor(doy)02-22	0.092** (0.045)	0.070 (0.056)
as.factor(doy)02-23	0.097** (0.045)	0.036 (0.056)
as.factor(doy)02-24	0.057 (0.045)	0.073 (0.056)
as.factor(doy)02-25	0.085* (0.045)	0.040 (0.056)
as.factor(doy)02-26	0.079* (0.045)	0.032 (0.056)
as.factor(doy)02-27	0.034 (0.045)	−0.049 (0.056)
as.factor(doy)02-28	0.035 (0.045)	0.077 (0.056)
as.factor(doy)02-29	0.260*** (0.078)	0.217** (0.097)
as.factor(doy)03-01	0.074* (0.045)	−0.021 (0.056)
as.factor(doy)03-02	0.005 (0.045)	0.022 (0.056)
as.factor(doy)03-03	0.116*** (0.045)	0.028 (0.056)
as.factor(doy)03-04	0.020 (0.045)	−0.036 (0.056)

as.factor(doy)03-05	0.064 (0.045)	0.046 (0.056)
as.factor(doy)03-06	0.074* (0.045)	0.016 (0.056)
as.factor(doy)03-07	0.053 (0.045)	0.006 (0.056)
as.factor(doy)03-08	0.036 (0.045)	−0.085 (0.056)
as.factor(doy)03-09	0.093** (0.045)	0.060 (0.056)
as.factor(doy)03-10	0.043 (0.045)	0.053 (0.056)
as.factor(doy)03-11	0.040 (0.045)	0.027 (0.056)
as.factor(doy)03-12	0.107** (0.045)	0.065 (0.056)
as.factor(doy)03-13	0.092** (0.045)	−0.042 (0.056)
as.factor(doy)03-14	−0.049 (0.045)	−0.076 (0.056)
as.factor(doy)03-15	−0.001 (0.045)	−0.032 (0.056)
as.factor(doy)03-16	0.013 (0.045)	−0.026 (0.056)
as.factor(doy)03-17	−0.048 (0.045)	−0.027 (0.056)
as.factor(doy)03-18	−0.012 (0.045)	−0.058 (0.056)
as.factor(doy)03-19	−0.034 (0.045)	−0.073 (0.056)
as.factor(doy)03-20	−0.096** (0.045)	−0.135** (0.056)
as.factor(doy)03-21	−0.030 (0.045)	−0.136** (0.056)
as.factor(doy)03-22	−0.131*** (0.045)	−0.176*** (0.056)
as.factor(doy)03-23	−0.053 (0.045)	−0.158*** (0.056)
as.factor(doy)03-24	−0.047 (0.045)	−0.133** (0.056)
as.factor(doy)03-25	−0.063 (0.045)	−0.079 (0.056)
as.factor(doy)03-26	−0.044 (0.045)	−0.120** (0.056)
as.factor(doy)03-27	−0.119*** (0.045)	−0.182*** (0.056)
as.factor(doy)03-28	−0.113** (0.045)	−0.139** (0.056)
as.factor(doy)03-29	−0.081* (0.045)	−0.049 (0.056)
as.factor(doy)03-30	−0.077* (0.045)	−0.175*** (0.056)
as.factor(doy)03-31	−0.073 (0.045)	−0.133** (0.056)
as.factor(doy)04-01	−0.082* (0.045)	−0.174*** (0.056)
as.factor(doy)04-02	−0.125*** (0.045)	−0.124** (0.056)
as.factor(doy)04-03	−0.080* (0.045)	−0.196*** (0.056)
as.factor(doy)04-04	−0.143*** (0.045)	−0.186*** (0.056)
as.factor(doy)04-05	−0.133*** (0.045)	−0.071 (0.056)
as.factor(doy)04-06	−0.126*** (0.045)	−0.132** (0.056)
as.factor(doy)04-07	−0.054 (0.045)	−0.138** (0.056)
as.factor(doy)04-08	−0.203*** (0.045)	−0.130** (0.056)
as.factor(doy)04-09	−0.112** (0.045)	−0.159*** (0.056)

as.factor(doy)04-10	−0.139*** (0.045)	−0.114** (0.056)
as.factor(doy)04-11	−0.057 (0.045)	−0.129** (0.056)
as.factor(doy)04-12	−0.109** (0.045)	−0.176*** (0.056)
as.factor(doy)04-13	−0.107** (0.045)	−0.110** (0.056)
as.factor(doy)04-14	−0.070 (0.045)	−0.145*** (0.056)
as.factor(doy)04-15	−0.075* (0.045)	−0.091 (0.056)
as.factor(doy)04-16	−0.139*** (0.045)	−0.144** (0.056)
as.factor(doy)04-17	−0.082* (0.045)	−0.137** (0.056)
as.factor(doy)04-18	−0.068 (0.045)	−0.153*** (0.056)
as.factor(doy)04-19	−0.079* (0.045)	−0.103* (0.056)
as.factor(doy)04-20	−0.111** (0.045)	−0.109* (0.056)
as.factor(doy)04-21	−0.035 (0.045)	−0.115** (0.056)
as.factor(doy)04-22	−0.065 (0.045)	−0.084 (0.056)
as.factor(doy)04-23	−0.037 (0.045)	−0.085 (0.056)
as.factor(doy)04-24	−0.057 (0.045)	−0.124** (0.056)
as.factor(doy)04-25	−0.089** (0.045)	−0.104* (0.056)
as.factor(doy)04-26	−0.066 (0.045)	−0.112** (0.056)
as.factor(doy)04-27	−0.035 (0.045)	−0.096* (0.056)
as.factor(doy)04-28	−0.099** (0.045)	−0.133** (0.056)
as.factor(doy)04-29	0.009 (0.045)	−0.081 (0.056)
as.factor(doy)04-30	−0.010 (0.045)	−0.128** (0.056)
as.factor(doy)05-01	−0.016 (0.045)	−0.102* (0.056)
as.factor(doy)05-02	−0.021 (0.045)	−0.102* (0.056)
as.factor(doy)05-03	0.031 (0.045)	0.009 (0.056)
as.factor(doy)05-04	−0.012 (0.045)	−0.058 (0.056)
as.factor(doy)05-05	−0.000 (0.045)	−0.050 (0.056)
as.factor(doy)05-06	−0.037 (0.045)	−0.025 (0.056)
as.factor(doy)05-07	0.007 (0.045)	−0.056 (0.056)
as.factor(doy)05-08	−0.040 (0.045)	−0.087 (0.056)
as.factor(doy)05-09	−0.126*** (0.045)	−0.122** (0.056)
as.factor(doy)05-10	−0.044 (0.045)	−0.041 (0.056)
as.factor(doy)05-11	−0.041 (0.045)	−0.057 (0.056)
as.factor(doy)05-12	−0.021 (0.045)	−0.127** (0.056)
as.factor(doy)05-13	−0.072 (0.045)	−0.037 (0.056)
as.factor(doy)05-14	−0.034 (0.045)	−0.127** (0.056)
as.factor(doy)05-15	−0.041 (0.045)	−0.056 (0.056)

as.factor(doy)05-16	0.013 (0.045)	−0.087 (0.056)
as.factor(doy)05-17	−0.033 (0.045)	−0.070 (0.056)
as.factor(doy)05-18	−0.030 (0.045)	−0.054 (0.056)
as.factor(doy)05-19	0.060 (0.045)	−0.069 (0.056)
as.factor(doy)05-20	0.049 (0.045)	−0.053 (0.056)
as.factor(doy)05-21	0.020 (0.045)	−0.040 (0.056)
as.factor(doy)05-22	−0.002 (0.045)	−0.108* (0.056)
as.factor(doy)05-23	0.037 (0.045)	−0.024 (0.056)
as.factor(doy)05-24	−0.031 (0.045)	−0.040 (0.056)
as.factor(doy)05-25	0.039 (0.045)	−0.035 (0.056)
as.factor(doy)05-26	0.038 (0.045)	−0.066 (0.056)
as.factor(doy)05-27	−0.006 (0.045)	−0.017 (0.056)
as.factor(doy)05-28	0.037 (0.045)	−0.049 (0.056)
as.factor(doy)05-29	−0.005 (0.045)	−0.052 (0.056)
as.factor(doy)05-30	−0.023 (0.045)	−0.059 (0.056)
as.factor(doy)05-31	−0.044 (0.045)	−0.071 (0.056)
as.factor(doy)06-01	−0.028 (0.045)	−0.062 (0.056)
as.factor(doy)06-02	−0.000 (0.045)	−0.097* (0.056)
as.factor(doy)06-03	0.022 (0.045)	−0.011 (0.056)
as.factor(doy)06-04	0.050 (0.045)	−0.074 (0.056)
as.factor(doy)06-05	0.009 (0.045)	−0.052 (0.056)
as.factor(doy)06-06	0.046 (0.045)	−0.043 (0.056)
as.factor(doy)06-07	−0.026 (0.045)	−0.048 (0.056)
as.factor(doy)06-08	−0.023 (0.045)	−0.041 (0.056)
as.factor(doy)06-09	0.002 (0.045)	−0.068 (0.056)
as.factor(doy)06-10	0.031 (0.045)	−0.016 (0.056)
as.factor(doy)06-11	0.014 (0.045)	−0.112** (0.056)
as.factor(doy)06-12	−0.006 (0.045)	−0.075 (0.056)
as.factor(doy)06-13	0.018 (0.045)	−0.067 (0.056)
as.factor(doy)06-14	−0.001 (0.045)	−0.062 (0.056)
as.factor(doy)06-15	0.044 (0.045)	−0.071 (0.056)
as.factor(doy)06-16	0.001 (0.045)	−0.112** (0.056)
as.factor(doy)06-17	−0.012 (0.045)	−0.125** (0.056)
as.factor(doy)06-18	0.021 (0.045)	−0.056 (0.056)
as.factor(doy)06-19	0.057 (0.045)	−0.109* (0.056)
as.factor(doy)06-20	−0.007 (0.045)	−0.114** (0.056)

as.factor(doy)06-21	−0.068 (0.045)	−0.062 (0.056)
as.factor(doy)06-22	0.018 (0.045)	−0.090 (0.056)
as.factor(doy)06-23	0.049 (0.045)	−0.080 (0.056)
as.factor(doy)06-24	0.020 (0.045)	−0.106* (0.056)
as.factor(doy)06-25	0.023 (0.045)	−0.051 (0.056)
as.factor(doy)06-26	0.059 (0.045)	−0.085 (0.056)
as.factor(doy)06-27	0.003 (0.045)	−0.083 (0.056)
as.factor(doy)06-28	0.058 (0.045)	−0.111** (0.056)
as.factor(doy)06-29	0.004 (0.045)	−0.087 (0.056)
as.factor(doy)06-30	0.076* (0.045)	−0.063 (0.056)
as.factor(doy)07-01	0.007 (0.045)	−0.177*** (0.056)
as.factor(doy)07-02	0.105** (0.045)	−0.022 (0.056)
as.factor(doy)07-03	0.090** (0.045)	−0.023 (0.056)
as.factor(doy)07-04	0.044 (0.045)	−0.116** (0.056)
as.factor(doy)07-05	0.030 (0.045)	−0.036 (0.056)
as.factor(doy)07-06	0.009 (0.045)	−0.084 (0.056)
as.factor(doy)07-07	0.071 (0.045)	−0.075 (0.056)
as.factor(doy)07-08	0.040 (0.045)	−0.029 (0.056)
as.factor(doy)07-09	0.004 (0.045)	−0.013 (0.056)
as.factor(doy)07-10	0.058 (0.045)	−0.017 (0.056)
as.factor(doy)07-11	−0.008 (0.045)	−0.075 (0.056)
as.factor(doy)07-12	0.063 (0.045)	−0.039 (0.056)
as.factor(doy)07-13	0.078* (0.045)	−0.070 (0.056)
as.factor(doy)07-14	0.081* (0.045)	−0.069 (0.056)
as.factor(doy)07-15	0.073 (0.045)	−0.049 (0.056)
as.factor(doy)07-16	0.002 (0.045)	−0.017 (0.056)
as.factor(doy)07-17	−0.005 (0.045)	−0.070 (0.056)
as.factor(doy)07-18	0.016 (0.045)	−0.098* (0.056)
as.factor(doy)07-19	0.006 (0.045)	−0.036 (0.056)
as.factor(doy)07-20	0.063 (0.045)	−0.058 (0.056)
as.factor(doy)07-21	0.061 (0.045)	−0.093* (0.056)
as.factor(doy)07-22	0.105** (0.045)	−0.055 (0.056)
as.factor(doy)07-23	0.120*** (0.045)	−0.079 (0.056)
as.factor(doy)07-24	0.069 (0.045)	−0.072 (0.056)
as.factor(doy)07-25	0.063 (0.045)	−0.044 (0.056)
as.factor(doy)07-26	0.079* (0.045)	−0.041 (0.056)

as.factor(doy)07-27	0.065 (0.045)	−0.063 (0.056)
as.factor(doy)07-28	0.095** (0.045)	−0.019 (0.056)
as.factor(doy)07-29	0.130*** (0.045)	−0.062 (0.056)
as.factor(doy)07-30	0.072 (0.045)	−0.088 (0.056)
as.factor(doy)07-31	0.078* (0.045)	−0.064 (0.056)
as.factor(doy)08-01	0.050 (0.045)	−0.144** (0.056)
as.factor(doy)08-02	0.051 (0.045)	−0.059 (0.056)
as.factor(doy)08-03	0.133*** (0.045)	−0.097* (0.056)
as.factor(doy)08-04	0.067 (0.045)	0.015 (0.056)
as.factor(doy)08-05	0.085* (0.045)	−0.066 (0.056)
as.factor(doy)08-06	0.120*** (0.045)	−0.052 (0.056)
as.factor(doy)08-07	0.096** (0.045)	−0.064 (0.056)
as.factor(doy)08-08	0.114** (0.045)	−0.043 (0.056)
as.factor(doy)08-09	0.062 (0.045)	−0.042 (0.056)
as.factor(doy)08-10	0.041 (0.045)	−0.069 (0.056)
as.factor(doy)08-11	0.043 (0.045)	−0.031 (0.056)
as.factor(doy)08-12	0.081* (0.045)	−0.039 (0.056)
as.factor(doy)08-13	0.082* (0.045)	−0.082 (0.056)
as.factor(doy)08-14	0.032 (0.045)	−0.084 (0.056)
as.factor(doy)08-15	0.060 (0.045)	−0.148*** (0.056)
as.factor(doy)08-16	0.052 (0.045)	−0.036 (0.056)
as.factor(doy)08-17	0.036 (0.045)	−0.071 (0.056)
as.factor(doy)08-18	0.023 (0.045)	−0.037 (0.056)
as.factor(doy)08-19	0.056 (0.045)	−0.057 (0.056)
as.factor(doy)08-20	0.026 (0.045)	−0.074 (0.056)
as.factor(doy)08-21	0.005 (0.045)	−0.040 (0.056)
as.factor(doy)08-22	0.037 (0.045)	−0.101* (0.056)
as.factor(doy)08-23	0.065 (0.045)	−0.013 (0.056)
as.factor(doy)08-24	0.069 (0.045)	−0.069 (0.056)
as.factor(doy)08-25	0.006 (0.045)	−0.036 (0.056)
as.factor(doy)08-26	0.009 (0.045)	−0.049 (0.056)
as.factor(doy)08-27	0.029 (0.045)	−0.039 (0.056)
as.factor(doy)08-28	−0.033 (0.045)	−0.102* (0.056)
as.factor(doy)08-29	−0.019 (0.045)	−0.134** (0.056)
as.factor(doy)08-30	−0.013 (0.045)	−0.022 (0.056)
as.factor(doy)08-31	0.008 (0.045)	−0.066 (0.056)

as.factor(doy)09-01	0.006 (0.045)	−0.099* (0.056)
as.factor(doy)09-02	−0.007 (0.045)	−0.132** (0.056)
as.factor(doy)09-03	−0.017 (0.045)	−0.075 (0.056)
as.factor(doy)09-04	0.014 (0.045)	−0.075 (0.056)
as.factor(doy)09-05	−0.012 (0.045)	−0.080 (0.056)
as.factor(doy)09-06	0.005 (0.045)	−0.072 (0.056)
as.factor(doy)09-07	−0.037 (0.045)	−0.069 (0.056)
as.factor(doy)09-08	0.061 (0.045)	−0.085 (0.056)
as.factor(doy)09-09	−0.009 (0.045)	−0.030 (0.056)
as.factor(doy)09-10	−0.023 (0.045)	−0.049 (0.056)
as.factor(doy)09-11	−0.029 (0.045)	−0.054 (0.056)
as.factor(doy)09-12	0.005 (0.045)	−0.049 (0.056)
as.factor(doy)09-13	0.037 (0.046)	0.077 (0.057)
as.factor(doy)09-14	0.007 (0.047)	−0.059 (0.059)
as.factor(doy)09-15	−0.003 (0.048)	0.009 (0.059)
as.factor(doy)09-16	0.054 (0.047)	−0.018 (0.060)
as.factor(doy)09-17	0.095** (0.047)	−0.017 (0.059)
as.factor(doy)09-18	0.021 (0.047)	0.000 (0.059)
as.factor(doy)09-19	0.038 (0.047)	−0.046 (0.059)
as.factor(doy)09-20	−0.022 (0.047)	−0.085 (0.059)
as.factor(doy)09-21	0.008 (0.047)	−0.030 (0.059)
as.factor(doy)09-22	0.067 (0.047)	−0.123** (0.059)
as.factor(doy)09-23	0.000 (0.047)	−0.005 (0.059)
as.factor(doy)09-24	0.032 (0.047)	0.012 (0.059)
as.factor(doy)09-25	0.008 (0.047)	0.006 (0.059)
as.factor(doy)09-26	0.063 (0.047)	−0.008 (0.059)
as.factor(doy)09-27	0.030 (0.047)	−0.067 (0.059)
as.factor(doy)09-28	0.068 (0.047)	−0.022 (0.059)
as.factor(doy)09-29	0.061 (0.047)	−0.085 (0.059)
as.factor(doy)09-30	0.004 (0.047)	−0.014 (0.059)
as.factor(doy)10-01	0.001 (0.047)	−0.016 (0.059)
as.factor(doy)10-02	0.023 (0.047)	−0.033 (0.059)
as.factor(doy)10-03	−0.030 (0.047)	−0.004 (0.059)
as.factor(doy)10-04	−0.002 (0.047)	−0.011 (0.059)
as.factor(doy)10-05	0.034 (0.047)	−0.092 (0.059)
as.factor(doy)10-06	−0.002 (0.047)	−0.016 (0.059)

as.factor(doy)10-07	0.034 (0.047)	−0.033 (0.060)
as.factor(doy)10-08	0.008 (0.047)	−0.052 (0.059)
as.factor(doy)10-09	−0.013 (0.047)	−0.021 (0.059)
as.factor(doy)10-10	0.027 (0.048)	0.046 (0.059)
as.factor(doy)10-11	−0.005 (0.047)	−0.084 (0.060)
as.factor(doy)10-12	−0.070 (0.047)	−0.080 (0.060)
as.factor(doy)10-13	0.002 (0.047)	−0.033 (0.059)
as.factor(doy)10-14	0.047 (0.047)	-0.137^{**} (0.059)
as.factor(doy)10-15	0.071 (0.047)	−0.014 (0.059)
as.factor(doy)10-16	0.042 (0.047)	−0.043 (0.059)
as.factor(doy)10-17	-0.082^{*} (0.047)	−0.043 (0.059)
as.factor(doy)10-18	−0.020 (0.047)	−0.053 (0.059)
as.factor(doy)10-19	−0.026 (0.047)	−0.064 (0.059)
as.factor(doy)10-20	−0.056 (0.047)	−0.052 (0.059)
as.factor(doy)10-21	−0.025 (0.047)	−0.033 (0.059)
as.factor(doy)10-22	0.020 (0.047)	−0.014 (0.059)
as.factor(doy)10-23	−0.020 (0.047)	−0.056 (0.059)
as.factor(doy)10-24	0.006 (0.048)	−0.082 (0.060)
as.factor(doy)10-25	−0.070 (0.047)	−0.076 (0.060)
as.factor(doy)10-26	−0.034 (0.047)	−0.052 (0.060)
as.factor(doy)10-27	−0.058 (0.047)	−0.061 (0.059)
as.factor(doy)10-28	-0.082^{*} (0.047)	−0.081 (0.059)
as.factor(doy)10-29	0.008 (0.047)	−0.038 (0.059)
as.factor(doy)10-30	−0.039 (0.047)	−0.092 (0.059)
as.factor(doy)10-31	-0.124^{***} (0.047)	-0.155^{***} (0.059)
as.factor(doy)11-01	−0.045 (0.047)	−0.019 (0.059)
as.factor(doy)11-02	−0.043 (0.047)	−0.053 (0.059)
as.factor(doy)11-03	−0.055 (0.047)	−0.064 (0.059)
as.factor(doy)11-04	−0.055 (0.047)	−0.073 (0.059)
as.factor(doy)11-05	−0.052 (0.047)	−0.064 (0.059)
as.factor(doy)11-06	0.017 (0.047)	0.049 (0.059)
as.factor(doy)11-07	-0.117^{**} (0.047)	−0.082 (0.059)
as.factor(doy)11-08	-0.113^{**} (0.047)	−0.092 (0.059)
as.factor(doy)11-09	−0.012 (0.047)	-0.121^{**} (0.059)
as.factor(doy)11-10	0.006 (0.047)	−0.097 (0.059)
as.factor(doy)11-11	−0.055 (0.047)	-0.109^{*} (0.059)

as.factor(doy)11-12	−0.059 (0.047)	−0.044 (0.059)
as.factor(doy)11-13	−0.056 (0.047)	−0.064 (0.059)
as.factor(doy)11-14	−0.083* (0.047)	−0.060 (0.060)
as.factor(doy)11-15	−0.070 (0.048)	−0.033 (0.060)
as.factor(doy)11-16	−0.110** (0.047)	−0.179*** (0.060)
as.factor(doy)11-17	−0.057 (0.047)	−0.037 (0.059)
as.factor(doy)11-18	−0.063 (0.047)	−0.070 (0.059)
as.factor(doy)11-19	−0.047 (0.047)	−0.060 (0.059)
as.factor(doy)11-20	−0.065 (0.047)	−0.109* (0.059)
as.factor(doy)11-21	−0.127*** (0.048)	−0.103* (0.060)
as.factor(doy)11-22	−0.077 (0.047)	−0.098* (0.059)
as.factor(doy)11-23	−0.115** (0.048)	−0.105* (0.059)
as.factor(doy)11-24	−0.074 (0.047)	−0.082 (0.059)
as.factor(doy)11-25	−0.074 (0.047)	−0.097 (0.059)
as.factor(doy)11-26	−0.038 (0.047)	−0.068 (0.059)
as.factor(doy)11-27	−0.011 (0.047)	−0.100* (0.059)
as.factor(doy)11-28	−0.125*** (0.047)	−0.122** (0.059)
as.factor(doy)11-29	−0.106** (0.047)	−0.097 (0.059)
as.factor(doy)11-30	−0.118** (0.047)	−0.138** (0.059)
as.factor(doy)12-01	−0.072 (0.047)	−0.115* (0.059)
as.factor(doy)12-02	−0.085* (0.047)	−0.101* (0.059)
as.factor(doy)12-03	−0.178*** (0.047)	−0.133** (0.059)
as.factor(doy)12-04	−0.080* (0.047)	−0.028 (0.059)
as.factor(doy)12-05	−0.111** (0.047)	−0.108* (0.060)
as.factor(doy)12-06	−0.086* (0.047)	−0.109* (0.059)
as.factor(doy)12-07	−0.116** (0.047)	−0.098* (0.059)
as.factor(doy)12-08	−0.166*** (0.047)	−0.099* (0.059)
as.factor(doy)12-09	−0.134*** (0.047)	−0.076 (0.059)
as.factor(doy)12-10	−0.094** (0.047)	−0.063 (0.059)
as.factor(doy)12-11	−0.092* (0.047)	0.010 (0.059)
as.factor(doy)12-12	−0.136*** (0.047)	−0.090 (0.059)
as.factor(doy)12-13	−0.194*** (0.047)	−0.121** (0.059)
as.factor(doy)12-14	−0.130*** (0.047)	−0.116* (0.059)
as.factor(doy)12-15	−0.114** (0.047)	−0.109* (0.059)
as.factor(doy)12-16	−0.097** (0.047)	−0.100* (0.059)
as.factor(doy)12-17	−0.078 (0.047)	−0.099* (0.059)

as.factor(doy)12-18	-0.099^{**} (0.048)	-0.167^{***} (0.059)
as.factor(doy)12-19	-0.108^{**} (0.047)	-0.189^{***} (0.060)
as.factor(doy)12-20	-0.093^{*} (0.047)	-0.217^{***} (0.059)
as.factor(doy)12-21	-0.102^{**} (0.047)	-0.077 (0.060)
as.factor(doy)12-22	-0.016 (0.047)	-0.071 (0.059)
as.factor(doy)12-23	-0.007 (0.047)	0.004 (0.059)
as.factor(doy)12-24	-0.179^{***} (0.047)	-0.206^{***} (0.059)
as.factor(doy)12-25	-0.439^{***} (0.047)	-0.428^{***} (0.060)
as.factor(doy)12-26	0.124^{***} (0.047)	-0.004 (0.059)
as.factor(doy)12-27	0.109^{**} (0.048)	-0.021 (0.059)
as.factor(doy)12-28	0.105^{**} (0.048)	-0.003 (0.060)
as.factor(doy)12-29	0.123^{**} (0.048)	0.102^{*} (0.060)
as.factor(doy)12-30	0.147^{***} (0.047)	0.057 (0.059)
as.factor(doy)12-31	-0.042 (0.047)	-0.113^{*} (0.059)
Constant	3.501^{***} (0.089)	2.623^{***} (0.111)
Observations	21,011	20,781
R^2	0.690	0.912
Adjusted R^2	0.684	0.910
Residual Std. Error	2.115 (df = 20585)	2.644 (df = 20355)
F Statistic	107.981^{***} (df = 425; 20585)	496.974^{***} (df = 425; 20355)

Notes: ***Significant at the 1 percent level.
**Significant at the 5 percent level.
*Significant at the 10 percent level.
